# Whole-genome sequencing and comparative genomics reveal candidate genes associated with quality traits in *Dioscorea alata*

**DOI:** 10.1186/s12864-024-10135-2

**Published:** 2024-03-06

**Authors:** Ana Paula Zotta Mota, Komivi Dossa, Mathieu Lechaudel, Denis Cornet, Pierre Mournet, Sylvain Santoni, David Lopez, Hana Chaïr

**Affiliations:** 1 UMR AGAP, CIRAD, 34398 Montpellier, France; 2 AGAP, Univ Montpellier, CIRAD, INRAe, Montpellier SupAgro, Montpellier, France; 3 Université Côte d’Azur, Institut Sophia Agrobiotech, INRAE, CNRS, Sophia Antipolis, PACA 06903 France; 4 CIRAD, UMR AGAP Institut, 97170 Petit Bourg, Guadeloupe France; 5 UMR Qualisud, CIRAD, F97130 Capesterre-Belle-Eau, Guadeloupe France; 6 QualiSud, Université Montpellier, Institut Agro, CIRAD, Avignon Université, Université de La Réunion, 34398 Montpellier, France

**Keywords:** Comparative genomics, Texture, Pectin, Starch, Flavonoids, *Dioscorea alata*

## Abstract

**Background:**

Quality traits are essential determinants of consumer preferences. *Dioscorea alata* (Greater Yam), is a starchy tuber crop in tropical regions. However, a comprehensive understanding of the genetic basis underlying yam tuber quality remains elusive. To address this knowledge gap, we employed population genomics and candidate gene association approaches to unravel the genetic factors influencing the quality attributes of boiled yam.

**Methods and Results:**

Comparative genomics analysis of 45 plant species revealed numerous novel genes absent in the existing *D. alata* gene annotation. This approach, adding 48% more genes, significantly enhanced the functional annotation of three crucial metabolic pathways associated with boiled yam quality traits: pentose and glucuronate interconversions, starch and sucrose metabolism, and flavonoid biosynthesis. In addition, the whole-genome sequencing of 127 genotypes identified 27 genes under selection and 22 genes linked to texture, starch content, and color through a candidate gene association analysis. Notably, five genes involved in starch content and cell wall composition, including 1,3-beta Glucan synthase, β-amylase, and Pectin methyl esterase, were common to both approaches and their expression levels were assessed by transcriptomic data.

**Conclusions:**

The analysis of the whole-genome of 127 genotypes of *D. alata* and the study of three specific pathways allowed the identification of important genes for tuber quality. Our findings provide insights into the genetic basis of yam quality traits and will help the enhancement of yam tuber quality through breeding programs.

**Supplementary Information:**

The online version contains supplementary material available at 10.1186/s12864-024-10135-2.

## Background

The releasing of varieties with improved quality (organoleptic and nutritional) is a target in the breeding process, and failure to achieve it can lead to the rejection of improved varieties. However, food quality is a complex attribute that reflects the preferences of all actors in the value chain: producers, processors, retailers and consumers. In recent years, there has been an increasing number of studies on the identification of genomic regions associated with quality traits, focusing on economically important crops such as rice, tomato and citrus, etc. [1–3]. Underutilized crops such as tropical root and tuber crops have been largely neglected. Nevertheless, root and tuber crops are among the most important crops for subsistence and commercial purposes after cereals.

Yams, of the Dioscoreaceae family, are important edible tuber crops, mainly in developing countries. The most widespread species is greater yam (*Dioscorea alata* L.), cultivated primarily for its starchy tubers in tropical regions [4]. Greater yam is unknown in the wild and its wild relatives have yet to be identified. Our demographic analysis supported an early divergence of Greater yam between mainland Asia and Oceania, probably followed by two centres of domestication [5]. It is a dioecious and autopolyploid species (2n = 2x = 40, 3x = 60 and 4x = 80) [6]. The recent publication of the genetic map [7] followed by the release of the reference genome of *D. alata* [8] have paved the way to work on the genetic architecture of the traits of interest. Genomic regions associated with anthracnose resistance, tuber oxidative browning, tuber morphological attributes, and dry matter have been revealed [8–10]. The publication of the genome assembly of other species of *Dioscoreae* also advanced the research for traits (quality traits and stress resistance) [11–14]. Although it is well known that tuber quality is important for varieties adoption in root and tuber crops [15], its genetic bases have not yet been comprehensively investigated.

Greater yam is consumed in various forms, mainly boiled, pounded, fried, or baked [16]. The quality of boiled yam, as characterised by sensory testing with consumers, is related to white colour, crumbly, sticky to the fingers, non-fibrous and easy to chew texture, sweet taste and good smell [16]. The choice of colour and the consistency of the tubers after cooking are essential characteristics that have been previously selected in other crops, such as cassava and potato [17]. Texture, which determines the ability of the raw material to soften after cooking while maintaining its firmness, is a complex trait. The main factors influencing texture are starch content and its distribution in the tuber, cell wall structure and composition, and level of degradation of the middle lamella of the cell wall [18]. The cell wall of most plants is composed of 90–95% polysaccharides, divided into cellulose, hemicellulose and pectin, and only 5–10% protein. Cell wall polysaccharides and starch content are principally associated with two metabolic pathways: pentose and glucuronate interconversions, and starch and sucrose metabolism [19]. The colour of the tubers is one of the most important varietal rejection factors by consumers. The colour is partly determined by genes in the flavonoid biosynthetic pathway, and recent studies also showed the oxidative browning of tubers which are associated with tuber colour [8, 20, 21]. Despite their importance for the main quality attributes of boiled yam, the flavonoid pathway has been very little studied.

Over the last decade, hundreds of genomes have been released from crop species (e.g. *Glycine max* [22], *Oryza sativa* [23], to orphan-crops (e.g. *Dioscorea rotundata* [11], *Manihot esculenta* [24]). Likewise, much effort has been put on improving the genome annotation in order to extract biological knowledge from genomic sequences [25]. However, annotating a genome is time and resources consuming. Automatic pipelines can produce inaccurate genome annotation and their results often require manual curation [26]. In non-model species, this can be especially true, since several genes lack a precise functional annotation, relying on public databases and gene similarity only. The use of comparative analysis allows to find homologous and orthologous genes from different species and within the species of interest [27], and predict more precisely their functions. Using the annotation produced with non-manual curated methods (similarity-based), together with comparative genomics methods can increase the number of genes with a functional annotation, and also help to better assign the functional annotation of other genes.

In this study, we re-sequenced the whole genome of 127 genotypes with different quality traits from across greater yam geographical distribution. We focused our work on the three metabolic pathways involved in texture and in starch content (pentose and glucuronate interconversions, starch and sucrose metabolism), and in colour (flavonoid biosynthesis). Using comparative genomics, we were able to improve the annotation of a number of genes, including those in the three major metabolic pathways targeted, thereby enriching the annotation of the *D. alata* genome. With a population genomics approach, we identified genes under selection. To assess the functional importance of candidate genes, we performed a phenotype-genotype association analysis using texture, colour and starch content related traits, and characterised the expression profiles of target genes using transcriptomic analysis. Overall, we generated a consistent and valuable amount of genomic resources for future research on important agronomic traits in yam.

## Methods

### Plant material

**Fig. 1:**
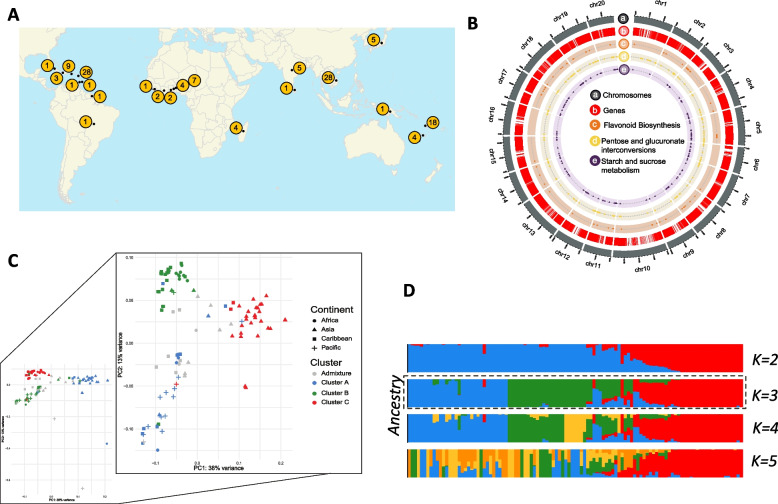
Dataset and population structure of *Dioscorea alata*. **A** Geographical origin of the 125 *Dioscorea alata* accessions (numbers correspond to number of accessions). **B** Circos plot of the 20 chromosomes of *D. alata.* From outer to the inner circle: chromosomes, gene density, localization of genes from pentose and glucuronate interconversions, genes from starch and sucrose metabolism and genes from flavonoid biosynthesis. **C** Principal component analysis of the 107 diploid genotypes of *D. alata* and detailed PCA without outliers. Colour codes correspond to the genetic clusters identified in the ADMIXTURE analysis for K = 3 and minimum ancestry threshold of 75%. **D** Population structure of the *D. alata* genotypes identified with ADMIXTURE analysis setting K = 2–5 number of clusters

This study used 125 genotypes from *Dioscorea alata* and two outgroup genotypes, belonging to *D. rotundata* and *D. trifida* species, collected in 19 different countries. Genotypes were selected to cover a maximum of worldwide diversity (Fig. 1; Supplemental Table 1) based on previous studies [5]. These genotypes were used to find genomic polymorphism, compared to the *D. alata* reference genome. From the whole set used in this study, a total of 45 *D. alata* accessions harbouring a high genetic and phenotypic diversity were planted from 2018 to 2020 in May each year and harvested at full maturity in the following year between January and March at each of three different locations in Guadeloupe, Godet (16°20′ N, 61°30′ 0.10 m above sea level (masl)), Roujol (16°10′ 56′′ N, 61° 35′ 24′′ W, 10 masl), and Duclos ((16°120′ N, 61°39′ O, 125 masl) [7]. The mean temperature for these locations was 25.8ºC, 27.1ºC, and 24.5ºC, respectively. For each genotype we used 10 seedlings in three biological replicates. These seedlings were planted 30 cm apart from each other in three different ridges 65 m long. The plant material was collected from Centre de Ressources Biologiques – Plantes Tropicales (CRB-PT), International Institute of Tropical Agriculture (IITA) and Plant Resources Center (PRC) genebanks, and named according to their accession numbers, as previously described in Sharif et al. [5].

### Phenotyping for quality traits

Tuber colour was analysed by computerized image analysis techniques (Supplemental Note 1). Images were collected on three tubers per genotype (*n* = 45 genotypes) and location (*n* = 3) during the 2018 cropping season. Acquired images were analysed using the Rvision library [28] with R programming language (R Core Team, 2021). We tested different colour indexes to characterise purple yam: whiteness index (WI), yellow index (YI), Colour Index of Red Grapes (CIRG), and HUE index. Finally, the Hue index was found to be discriminant within the studied diversity panel. Brown index (BI) was also calculated according to [29].

Starch content was predicted using near infrared spectroscopy (NIRS). Two replicates of yam flour samples were scanned with a FOSS-NIRSystems model 6500 scanning monochromator (FOSS-NIRSystems, Silver Spring, MD, USA) equipped with an autocup. The spectroscopic procedures and data recording were conducted with ISIscan™ software (FOSS, Hillerød, Denmark). The model was calibrated using 2016 and 2017 data and validated on an external independent 2018 dataset. (Supplemental Note 2).

The texture was measured on steam-cooked yam by penetrometry using the TAX-TPlus texture analyzer (Stable Micro Systems, Ltd., Surrey, UK). Yam tubers harvested at Godet 2019 were used for this analysis. Three tubers per variety were sampled and divided into three equal sections (proximal, distal, central) and prepared as described in Supplemental Note 3. Each part was used to produce three cubes at the central section. Each cube was steam-cooked up to 15 min, followed by a cooling time of 7 min, corresponding to a cube temperature of 45 °C. Total area was calculated from the puncture test, while four texture profile analyzer parameters were computed from the force–time curve: hardness (N), cohesiveness, gumminess, and springiness (Supplemental Note 3).

### Whole-genome sequencing

The genomic DNA from 127 genotypes of *Dioscorea spp*. was extracted from leaves according to a protocol using mixed alkyl trimethylammonium bromide (MATAB) buffer and NucleoMag Plant Kit (Macherey–Nagel, Germany) already described by [30]. Sequencing libraries were prepared as described in Dossa et al. [21]. Paired-end high throughput sequencing (2 × 150 bp) was performed on an Illumina NovaSeq 6000 instrument on GeT-PlaGe platform, (Toulouse, France) and Genewiz company (Leipzig, Germany).

### Variant discovery and filtering

The whole-genome sequences of 127 Dioscorea spp. genotypes were firstly quality checked using FastQC Version 0.11.7 [31] then mapped to the *D. alata* reference genome v.2 with a size of 479.5 Mb [8], using BWA-MEM version 0.7.15 [32]. The quality of the mapping was analysed with Qualimap version 2.2.2 [33]. The output from mapping of individual genotypes was used for variant discovery using GATK 4.1.6.0 [34].

The duplicated reads were marked for further analysis using MarkDuplicatedSpark and used as input for variant calling individually with HaplotypeCaller in the GVCF mode. The GVCF files were consolidated with GenomicDBImport per chromosome. The consolidated VCF file for all the 127 genotypes was produced by GenotypeGVCF. A database with the genome and predicted proteome of *D. alata* was created using SnpEff software version 5.0e [35]. Using this database, all the polymorphisms were annotated for their type, and effect in the nucleotide sequence.

The filtering and statistical analysis of polymorphism was performed using VCFtools version 0.1.16 [36]. For the filtering, the following parameters were used: –max-missing 0.5 –minQ 30 –minDP 10 –maxDP 200. For gene candidate purposes, we selected only the polymorphisms annotated with a high impact on the amino acid sequences.

### Genetic population analyses

Among all the 127 genotypes of *Dioscorea* spp*.* with different ploidy levels, 107 diploid genotypes (Supplemental Table 1) were selected for further population genetics analyses. We explored the population structure using the original filters of vcftools described above, in addition to: –maf 0.01 –max-alleles 2 –min-alleles 2 –remove-indels –max-missing 0.5 –thin 10 –ld-window 50 –min-r2 0.1. The final VCF file with 12,761,120 SNPs obtained from this analysis was used for population structure analysis using ADMIXTURE version 1.23 [37]. We tested the replicates from *K* = *1* to *15*, and the most probable *K* was determined by the smallest cross-validation error. We then limited the ancestry threshold to 75%, where genotypes with a value lower than this were considered in the admixted group. After Bayesian clustering analysis, populations were redefined according to the results obtained. For the Principal Component Analysis, we used plink2 (www.cog-genomics.org/plink/2.0/) to produce an eigen file and we used an in-house R script for the graph. To identify genes under selection, *Fst* analysis was conducted using vcftools. We used the clusters defined by the admixture analysis, and compared each cluster against all the other clusters, using vcftools with an *Fst*-window-size of 50 kb and a *Fst*-window-step of 10 kb. Candidates were defined as those corresponding to the top 5% most important values of the *Fst* theoretical distribution. Due to the non-normal distribution of *Fst* values, candidate univariate distributions (Weibull, normal, lognormal, and gamma) were fitted to the *Fst* cumulative distribution function using the fitdist function [38]. The Weibull distribution was determined to best fit the observed data based on the respective AIC values. The quantile function of the Weibull distribution was used to identify a *Fst* cutoff value for each comparison, which was subsequently employed to select candidates. Afterward, we searched for the genes on these regions using BEDTools intersect version 2.29 [39]. The gene ontology enrichment analysis of the genes from outlier regions was performed with KOBAS-i webserver [40] in order to find out their biological significance.

### Comparative genomics and orthology analyses

For comparative genomics analyses 45 species from diverse plant lineages were selected (Supplemental Table 2). The species used in this analysis were chosen based on the completeness of their genomes, phylogenetic distribution along the tree of viridiplantae and their use as plant-models. These plants belong to the monocotyledons and dicotyledons, encompassing 17 different clades. Five genomes belonging to Dioscoreales were included (*Dioscorea alata, D. rotundata, D. dumetorum, D. zingiberensis, Trichopus zeylanicus*). The completeness score of each plant proteome was verified by BUSCO [41] using the Viridiplantae dataset of BUSCO. We chose a threshold of 70% of completeness to include public proteomes. For the inference of orthologous groups, OrthoFinder software v2.4.0 [42] was used with the default parameters. Gene presence and absence of species in orthogroups was assessed by UpSetR [43], using a binary matrix as input.

### Metabolic pathways selection and gene retrieval from *D. alata*

We downloaded the complete list of enzyme codes (EC) from the pathway of pentose and glucuronate interconversions (map00040), starch and sucrose metabolism (map00500) and flavonoid biosynthesis (map00941) from KEGG database (https://www.genome.jp). These EC numbers were used to retrieve the genes annotated in the GFF file of *D. alata* (https://phytozome-next.jgi.doe.gov/info/Dalata_v2_1)*.* The genes annotated with these EC numbers were further searched in the orthogroups produced by our comparative genomics study, and all the homologous genes from *D. alata* were retrieved. The VCF file was reduced to the coding region (including introns, 5’UTR and 3’UTR) of the selected genes of each pathway.

### Candidate gene association analysis

Three VCF files, one for each target metabolic pathway genes, containing non-synonymous SNPs were extracted from the whole VCF file and further filtered for minor allele frequency ≥ 0.05, missing rate < 20% using TASSEL5.0 [44]. The associations between the polymorphisms from each metabolic pathway candidate genes and the corresponding phenotypic traits were tested using TASSEL5.0 based on the General Linear Model. Only significant variants with *P* ≤ 0.001 were retained. The association analysis was conducted with phenotypic data from each location independently. The effect of alleles at significant SNPs was assessed by comparing phenotyping data for allelic groups. A Student's t-test was used to compare the groups of haplotypes (*P* < 0.05) in the R4.0.23 software with the “ggpubr” packages and “rstatix”.

### Transcriptome analysis of six *D. alata* genotypes

RNA was extracted from six of the *D. alata* most diverse genotypes (CRB96, Roujol49, Roujol75, Roujol62, CRB47, and Roujol9), based on the extensive phenotypic characterization provided by [9]. Three biological replicates for each genotype were used. For each genotype, a 50 mg sample cut from the middle of freshly harvested tuber was ground in liquid nitrogen and total cellular RNA was extracted using a Sigma-Aldrich (St. Louis, MO) Spectrum™ Plant Total RNA kit with a DNAse treatment. This was followed by quantification by the Invitrogen (Carlsbad, CA) Quant-iT™ RiboGreen® RNA Reagent based on the manufacturer's protocol and verification of RNA quality by 5200 Fragment Analyzer™ System (Agilent, Santa Clara, CA) profile.

Synthesis of cDNA and construction of libraries were done with Illumina, Inc. TruSeq RNA Sample Preparation v2 Kit (San Diego, CA). Fragment size of selected cDNA were between 200 and 400pb. The 18 libraries were indexed, mixed and sequenced using one lane of an Illumina HiSeq 3000 sequencer with the 2 × 150 cycles, paired-end, indexed protocol (Genewiz facility, Liepzig, Germany).

The quality of the raw reads from the 18 libraries were assessed by FastQC version 0.11.7 [31]. The low-quality sequences and the Illumina adapters were trimmed by Trimmomatic version 0.39 [26]. Trimmed reads were quantified using Kallisto version 0.46.1 [45] using the reference genome of *D. alata* [8]. For the expression values we used the -log2 of the average value of transcripts per million over the three biological replicates. Expression values were used to determine the effect of the alleles on each genotype. A functional annotation was performed using the conserved domains of PFAM version 35 and a gene ontology annotation using Pfam2go (http://current.geneontology.org/ontology/external2go/pfam2go).

All the bioinformatics analyses were performed with the support of MESO@LR-Platform at University of Montpellier, CIRAD UMR-AGAP HPC (South Green Platform) and IFB core cluster.

## Results

### Patterns of genome-wide variation, population structure and signatures of selection

We generated the whole-genome sequences of 127 genotypes of *Dioscorea* spp. from diverse origins (Africa, Caribbean, Pacific and Asia) (Fig. 1A) using short-read sequencing with a mean coverage of 37X and an average quality of 40 (Supplemental Fig. 1, Supplemental Table 1). The polymorphism analyses in *D. alata* genotypes yielded a total of 63 M SNPs after filtering (Supplemental Fig. 2A). Most of the SNPs were found in the intergenic regions (48.11%), followed by downstream and upstream gene regions (19%), intronic regions (7.8%) and exonic regions (2.8%) (Fig. 1B, Supplemental Fig. 2B). For population genomics analyses, only diploid genotypes (107) were used (Supplemental Table 1), since diploid genotypes represent the overall diversity of the species as demonstrated previously [5]. Three major groups were defined by the phylogenetic tree (Supplemental Fig. 3A) and largely supported by the principal component analysis (PCA). A first group consisting mainly of Asian genotypes, a second group which includes most Pacific genotypes, and a final group of African and Caribbean genotypes (Fig. 1C), which agrees with our previous study, performed with the genotype-by-sequencing analysis on a larger *D. alata* sample [5]. The Admixture analysis separated the genotypes into four clusters (Fig. 1D). However, as the fourth group had only six genotypes, a robust statistical analysis could not be conducted. Consequently, we conducted our further analysis with *K* = *3*. The cluster A was mainly composed by Pacific representatives and, to a lesser extent, African and Caribbean genotypes, while the cluster B was composed mostly by representatives from Africa and Caribbean, and finally the cluster C had mostly representatives from Asia (26 out of 30) (Fig. 1D, Supplemental Fig. 3B), which is in accordance with the PCA results. Whole sample nucleotide diversity was low (π = 0.407e^−4^), but π values were significantly different between clusters, with the highest values obtained for the cluster A and C (π = 0.399e^−4^ and 0.396e^−4^, respectively) and the lowest for the cluster B (π = 0.33e^−4^).

**Fig. 2:**
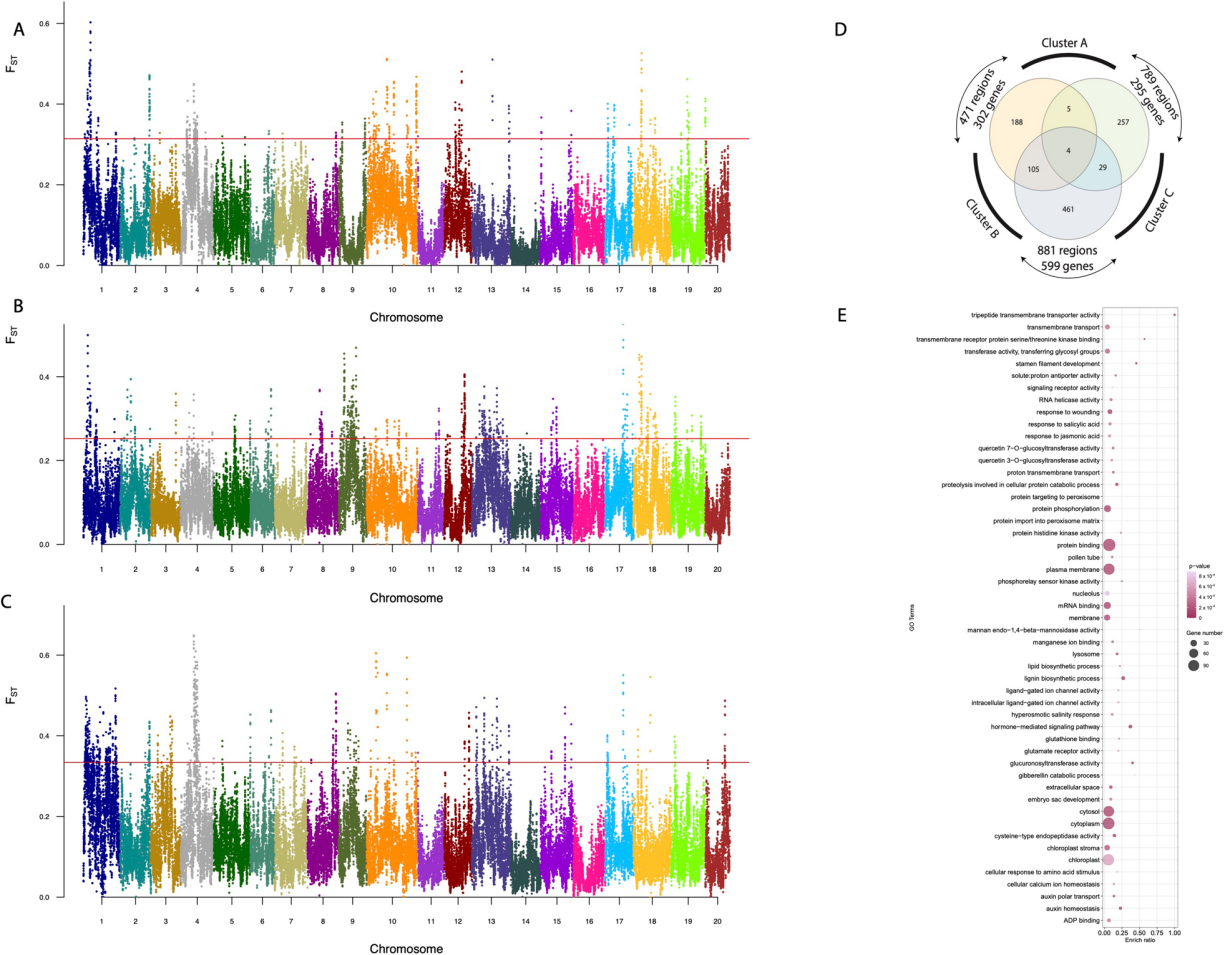
Selection signature found with Fst-outlier test. Fst was calculated between pairs of clusters: (**A**) A *versus* B, (**B**) A *versus* C, (**C**) B *versus* C. Each point represents a 50 Kb window and each colour represents one chromosome. The 5% highest Fst values (red line) were considered outliers. **D** Venn diagram of the common outlier regions across the three pairwise analyses. **E** The gene ontology enrichment of genes found in outlier regions against the whole set of genes of *D. alata*

**Fig. 3:**
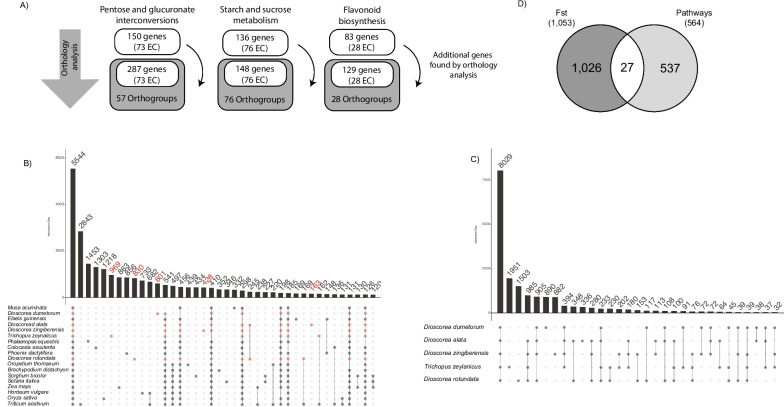
Orthology analysis of 45 plant species. **A** Number of genes found in the keyword analysis per pathway and their associated EC number. Number of genes found in the orthology analysis, number of EC numbers and number of orthologous groups found (dark grey). **B** UpSetR of the comparison of all the monocotyledon plants used in orthology analysis. Each dot represents a connection, and the bar represents the number of orthologous groups found for each association. The red dots show the species from the Dioscoreales order, and the red numbers represent the exclusive associations of Dioscoreales. **C** UpSetR of the five Dioscoreales species. **D** Intersection between the genes found by the *Fst-outlier* analysis and those involved in the three target pathways

To evaluate the distribution of genetic variance among the three clusters, we calculated the fixation index values (*Fst*) for each pair of clusters by windows of 50 kb and a step-window size of 10 kb. *Fst* values ranged from 7.85e-06 to 0.64, and we found in total 2,141 outlier regions *i.e.,* above the cut-off defined for each comparison using a Weibull distribution analysis (Fig. 2A, B and C, Supplemental Fig. 4). The comparison of Cluster B *vs* C showed the highest values of *Fst* and the highest number of outlier regions (881, Fig. 2C and Supplemental Fig. 4), followed by the comparison of A *vs* C (789, Fig. 2B and Supplemental Fig. 4) then A *vs* B (471, Fig. 2A and Supplemental Fig. 4). These outlier regions harboured a total of 1,053 genes after eliminating repeated genes among the three comparisons. We found 11 outlier regions in common among the three genetic clusters, due to their distant evolution, this could be the result of an early purifying selection in *D. alata*. Only four genes were present in these 11 common outlier regions (Fig. 2D): two closely located genes on chromosome 1, both annotated as non-specific serine/threonine kinase (EC:2.7.11.1), and two closely located genes on chromosome 4, both annotated as proteasome endopeptidase complexes (EC:3.4.25.1). Functional analysis of the 1,053 genes based on Gene Ontology (GO) terms showed that various functional pathways were enriched. The most enriched GO terms were transmembrane transport, protein phosphorylation, response to wounding, hormone signalling and lignin biosynthetic process (Fig. 2E).

**Fig. 4:**
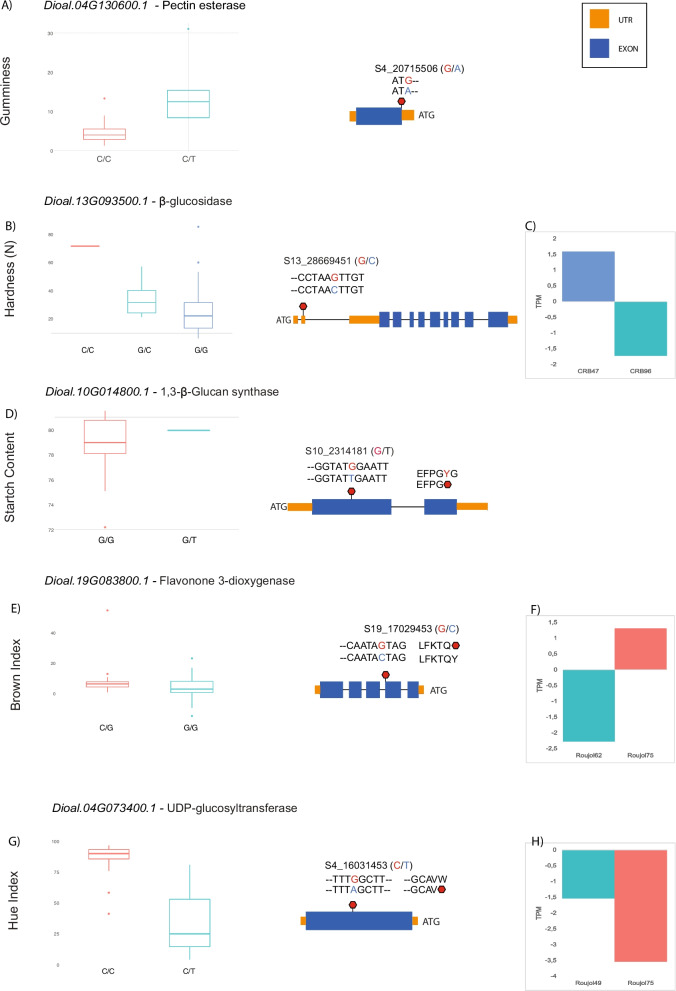
Selected genes associated with tuber-quality phenotypes, expression profile, and allelic effect on colour and texture traits. **A** Allele effect of S4_20715506 on gumminess and gene structure of Dioal.04G130600; **B**: Allele effect of S13_28669451 on Hardness and gene structure of Dioal.13G093500; **C**: Transcripts per million of two contrasting genotypes for Dioal.13G093500; **D**: Allele effect of S10_2314181 on Starch content and gene structure of Dioal.10G014800; **E**: Allele effect of S19_17029453 and gene structure of Dioal.19G083800; **F**: Transcripts per million of two contrasting genotypes of Dioal.19G083800. G: Allele effect of S4_16031453 on Hue Index; H: TPM of Dioal.04G073400 for two contrasting genotypes. Means were separated by two-tailed *t*-test at 0.05 probability. The bar color for the TPM graph correspond to the alleles on the allele effect graph

### Search of genes involved in pathways associated with tuber quality using combined strategies

To better understand the genetic basis of *D. alata* tuber quality, we focused on the genes described to be involved in one of the three pathways targeted (pentose and glucuronate interconversions, starch and sucrose metabolism, and flavonoid biosynthesis). To obtain the most complete set of genes from these pathways, we used two complementary strategies: a keyword search and a comparative genomics approach. For the first strategy, we searched for genes in *D. alata* reference genome [8] with enzyme commission numbers (EC number) corresponding to each one of the three pathways selected. In total, we could retrieve 322 genes from these pathways encompassing 177 EC numbers (Fig. 3A; Supplemental Table 2). The pentose and glucuronate interconversions pathway included most genes (150) and EC numbers (73), followed by starch and sucrose metabolism (136 genes, 76 EC numbers) and flavonoid biosynthesis metabolism (83 genes, 28 EC numbers). To complement this set of annotated genes, a comparative genomics approach was employed using 45 diverse plant genomes [27] (Supplemental Table 3; Supplemental Fig. 5, and 6). Groups of orthologous predicted proteins (OG) from different plants were used to infer the function of proteins that were not annotated by other methods. Since the clustering of proteins in OG does not rely on their predicted function but rather on their protein sequence, this method can uncover genes missed by keyword search. The orthology analysis resulted in a total of 58,916 OG from 1,706,645 proteins (Supplemental Data 1). Out of the total proteins analysed, 93.7% could be assigned to an OG. We identified 3,982 OG that are commonly found in all 45 species, despite their significant evolutionary distance. As expected, species that are closely related phylogenetically have a greater number of OG in common than distant ones (Supplemental Fig. 6). When we narrowed our comparison to monocotyledons (18 species), we found 5,544 OG shared among the species. Only three OG were not present in *D. alata* (Fig. 3B). The low number of missing groups in *D. alata* species was also observed by Bredeson et al*.*, when comparing it with species exclusively from the Dioscoreales order.

Among the five Dioscoreales species, we found 8,029 OG in common. *Trichopus zeylanicus* showed the highest number of exclusive OG (1,951) compared to the other *Dioscorea* species (*D. dumetorum*, *D. rotundata*, *D. zingiberensis*, and *D. alata*) and *D. alata* the lowest one (336) (Fig. 3C). Additionally, *D. alata* and *D. rotundata* had a high similarity, and likely underwent the same duplication events [8]. We identified 14 OG that comprise 163 exclusive proteins of Dioscoreales (Supplemental Table 4), with most of them (39) belonging to *D. alata,* potentially because of its better annotated genome*.* Based on their EC numbers, most of these OG are hydrolases. Note that these genes are not exclusive to *Dioscorea* species, and the high number of species-specific OG may be a technical artefact due to low sequence conservation within the gene family or lack of good structural annotation.

For most of the OG found in the three pathways, all or most of the 45 species had gene copies (Supplemental Data 1). Although present in all species, indicating the importance of these proteins in common biological processes, the number of proteins for each OG was uneven, which could be the result of assembly and annotation discrepancies, as well as duplication events that occurred after the differentiation of certain species (in-paralogs).

The comparative analysis allowed increasing the number of genes annotated with the same EC numbers for each pathway with the keyword approach [8]. We found additional 137 genes for the pentose and glucuronate interconversions pathway, 12 genes for starch and sucrose metabolism pathway and 46 genes for flavonoid biosynthesis metabolism (Fig. 3A; Supplemental Fig. 7, 8, 9). We were able to associate one orthologous group to each EC number, which contains one or more genes, except for the pentose and glucuronate interconversions pathway, for which the 73 EC numbers were collapsed in only 57 OG. After combining these two approaches, we could retrieve a total of 564 genes from 161 OG for the three pathways, representing 2.4 Mb from the total of the genome size. The comparative analysis allowed an increase of 48% more genes than using only the keyword search strategy. These genes were distributed across the whole *D. alata* genome, with chromosomes 2, 5, 11, and 17 having the highest number of genes and chromosome 16 the lowest (Fig. 1B).

We restricted the study of polymorphism within the genes found in the three pathways of interest and more specifically in the open reading frame regions for further analysis. A total of 406,325 SNPs on 564 candidate genes from the three pathways was selected. By keeping only SNPs annotated as high impact by snpEff (*i.e.* non-synonymous, stop codon gain, frame shift), which are responsible for the most significant differences on the protein sequences, a final number of 4,287 SNPs was used. The pathway of pentose and glucuronate interconversions had the highest number (2,912), followed by starch and sucrose pathway (765) and flavonoid biosynthesis (610).

### Identification of genes under selection in the three targeted pathways

Once we have determined the complete set of *D. alata* genes belonging to the three pathways, we searched for regions under selective pressure, using *Fst* analysis. When comparing the 564 genes from the three pathways to the 1,053 genes included in the *Fst* outlier windows, we found 27 genes in common, including nine genes found exclusively through the comparative genomic approach (Fig. 3D, Supplemental Table 5). The majority of the 158 SNPs on these 27 candidate genes were on the pentose and glucuronate interconversions pathway (119 polymorphisms in 10 genes) only found between Cluster B (mostly African genotypes) and C (Asian genotypes), followed by starch and sucrose (23 polymorphisms in 7 genes) and flavonoids (16 polymorphisms in 10 genes).

On the pentose and glucuronate interconversions pathway, we found three pectate lyase genes (*Dioal.01G025700.1*, *Dioal.01G025800.1*, *Dioal.01G035100.1*), which together with the polygalactunorase gene (PG) (*Dioal.06G005800.1*) and pectin methylesterase gene (PME) (*Dioal.08G105700.1*) are known as pectin-degrading enzymes [46]. PME family acts prior to hydrolysis by PG enzyme that degrades the homogalacturonate backbone (polygalacturonate). Pectate lyase gene family is responsible for the cleavage of pectate, which is the product of pectin degradation. From the same pathway, we also found four malectin-like receptor-like kinase genes (MLD) under selection (*Dioal.20G055000.1*, *Dioal.20G055100.1*, *Dioal.20G055200.1*, *Dioal.20G055300.1*).

On the starch and sucrose pathway, seven genes were under selection. Among them, two cellulose synthase genes (*Dioal.08G106000* and *Dioal.08G130500*), were under selection between clusters B (African and Caribbean gene pool) and C (Asian gene pool). Additionally, we identified a starch synthase gene (*Dioal.09G032200*) and a nudix-hydrolase (*Dioal.11G000200*), under selection among A (Pacific gene pool) and C and among A and B, respectively, a 1,3-beta-glucan synthase (*Dioal.10G014800*), among A and B and a trehalose-6-phosphate phosphatase (*Dioal.04G184200*) under selective pressure among A and C genetic clusters. Lastly, we found a beta-amylase gene (*Dioal.04G168200*) under selection. The beta-amylase gene family was previously described to increase the firmness of cooked sweet potatoes when its enzyme activity is low [47].

On the flavonoids pathway, 2-oxoglutarate-dependent dioxygenase (*Dioal.01G016100* and *Dioal.01G016200*) and chalcone isomerase (*Dioal.08G131200.1*) were found under selection.

### Candidate gene association studies for quality traits in *D. alata*

To get a deeper insight on the role of the genes from the three metabolic pathways in the tuber quality, we conducted a candidate gene association study. We used phenotypic data collected from two-years field trials on 45 *D. alata* genotypes selected to cover the genetic diversity. A set of 10 traits related to colour indices, starch content, and texture were measured. Texture was measured with two different types of probes. A conical probe was used to measure the total area (N sec), which reflects the total force required to penetrate the sample at a constant speed. A flat plate probe was used during a double compression cycle to measure hardness, gumminess, springiness, and cohesiveness (Supplemental note 1, 2 and 3). Descriptive statistics of the measured tuber quality traits are shown in Supplemental Table 6. The colour indices BI (Brown Index) and HI (Hue Index for purple colour) exhibited a wide range of variation (12.57–143 and -11.99–99.92, respectively), showing the presence of different coloured yam tubers in the association panel. The tuber starch content (SC) was on average 77.93 ± 2.10. The textural traits also varied extensively within the panel, indicating different boiled yam qualities of the accessions (Supplemental Table 6). Taken together, our results indicate a wide variation in the tuber quality traits across the 45 genotypes and pinpoint that these traits are largely quantitative in nature and thus can be assessed by an association study.

Using a generalized linear model, we obtained significant associations between SNPs and the phenotypic data. We detected a total of 22 quantitative trait nucleotides (QTNs) distributed on 10 chromosomes, for seven out of the ten traits evaluated in this study (Table 1).

**Table 1:** Marker trait association for the tuber quality traits detected by the generalized linear model

Trait	SNP_ID	Chr	Allele		Consequence Type	Genomic Location	Effect (%)	Gene Model	Predicted Annotation	Pathway	QTN
			**Major**	**Minor**							
Springiness	S1_10223458**	1	C	T	Stop gain	p.Trp287 > STOP	0.38	*Dioal.01G024400.1*	Leucine-rich repeat	Pectin	*qSp1.1*
	S1_10224508**	1	G	A	Stop gain	p.Gln104 > STOP	0.36	*Dioal.01G024400.1*	Leucine-rich repeat	Pectin	*qSp1.2*
	S2_19082482**	2	C	T	Stop gain	p.Gln1032 > STOP	-0.05	*Dioal.02G062700.1*	Protein kinase	Pectin	*qSp2.1*
	S2_19095245	2	C	G	Stop gain	p.Tyr578 > STOP	-0.04	*Dioal.02G062800.1*	Protein kinase	Pectin	*qSp2.2*
	S13_11781422	13	A	G	splice_acceptor_variantintron_variant	c.63-2A > G	-0.37	*Dioal.13G053400.1*	Glucose isomerase	Pectin	*qSp13.1*
	S20_14416887**	20	G	T	Stop gain	p.Gly381 > STOP	-0.46	*Dioal.20G055400.1*	Malectin-like	Pectin	*qS20.1*
Gumminess	S4_20715506	4	C	T	Start Loss	p.Met1?	-3.97	*Dioal.04G130600.1*	Pectin methylesterase	Pectin	*qG4.1*
	S7_6378890	7	G	A	splice_donor_variant intron_variant	c.-109 + 1G > A	-9.23	*Dioal.07G051400.1*	Alpha amylase	Starch	*qG7.1*
	S13_11783621	13	G	T	Stop gain	p.Glu208 > STOP	-2.61	*Dioal.13G053400.1*	Glucose isomerase	Pectin	*qG13.1*
	S13_11784207	13	A	G	splice_acceptor_variant intron_variant	c.723-1G > A	23.60	*Dioal.13G053400.1*	Glucose isomerase	Pectin	*qG13.2*
	S19_24800556	19	G	A	splice_donor_variant intron_variant	c.537 + 2C > T	4.87	*Dioal.19G187400.1*	Pectinesterase	Pectin	*qG19.2*
Total area	S2_18814720**	2	T	A	Stop gain	p.Tyr289 > STOP	21.88	*Dioal.02G061200.1*	Protein kinase	Pectin	*qTa2.1*
	**S4_20715506**	4	C	T	Start Loss	p.Met1?	-27.90	*Dioal.04G130600.1*	Pectin methylesterase	Pectin	*qTa4.1*
	S7_16450864	7	G	T	Stop gain	p.Tyr228 > STOP	-29.42	*Dioal.07G067100.1*	4-Alpha-glucanotransferase	Starch	*qTa7.1*
Hardness	S13_28669451	13	C	G	splice_donor_variant intron_variant	c.-837 + 1G > C	62.22	*Dioal.13G093500.1*	Beta-glucosidase	Starch	*qH13.2*
	S20_14334067**	20	G	T	Stop gain	p.Gly6 > STOP	-12.80	*Dioal.20G055000.1*	Malectin-like	Pectin	*qH20.1*
	S20_14364743**	20	A	C	Stop loss	p.Ter592Serext*?	10.75	*Dioal.20G055100.1*	Malectin-like	Pectin	*qH20.2*
Starch Content	**S10_2314181**	10	G	T	Stop gain	p.Gly35 > STOP	-14.75	*Dioal.10G014800.1*	1,3-beta-Glucan synthase	Starch	*qSC10.1*
	**S15_23348104**	15	T	C	splice_donor_variant intron_variant	c.1029 + 2C > T	4.99	*Dioal.15G119500.1*	beta-Amylase	Starch	*qSC15.2*
	S17_19982533	17	T	C	splice_acceptor_variant intron_variant	c.811-2A > G	-9.70	*Dioal.17G112400.1*	Glucose-1-phosphate adenylyltransferase	Starch	*qSC17.1*
Brown Index	S19_17029453	19	G	C	Stop gain	p.Tyr339 > STOP	34.69	*Dioal.19G083800.1*	Flavanone 3-dioxygenase	Flavonoid	*qYI19.1*
HUE Index	S4_16031453	4	C	T	Stop gain	p.Trp272 > STOP	50.28	*Dioal.04G073400.1*	UDP-glucosyltransferase protein	Flavonoid	*qHI4.1*

Bold = Genes found in *Fst* analysis

^**^ = Genes found by Orthology analysis

In addition to the phenotypic data, we produced the transcriptome data for six genotypes of *D. alata* from the association panel, to assess whether the polymorphisms found in the previous analyses had an influence on the gene expression. Among the 564 genes found in the three pathways, only 62 genes had no expression in the six genotypes (Supplemental Table 7). Most non-expressed genes were found on the pentose and glucuronate interconversions pathway (64%). Then, we focused on the candidate genes identified by the association analysis (Table 1, Supplemental Fig. 10).

Out of the 22 QTNs, 17 were associated with texture traits, with fourteen and three within pentose and glucuronate interconversions, and sucrose and starch pathways, respectively (Table 1 and Supplemental Fig. 10). Within pentose and glucuronate interconversions pathway, we found pectin esterase (*Dioal.19G187400.1*) and xylose isomerase genes (*Dioal.13G053400*) associated to springiness and gumminess. Leucine-rich repeat (*Dioal.01G024400)*, protein kinases (*Dioal.02G062700*, *Dioal.02G062800*), a malectin-like encoding gene (*Dioal.20G055400*) were associated to springiness and had a stop gain modification. Two other malectin-like genes found under selection were associated with hardness. Within starch and sucrose pathway we found three candidate genes associated with gumminess, total area and hardness, ɑ-amylase (*Dioal.07G051400.1*), 4-ɑ-glucanotransferase *(Dioal.07G067100)* and beta-glucosidase (*Dioal.13G093500*), respectively. Using the transcriptome data produced for six different genotypes from the panel, we compared the expression values and the expressed alleles of each candidate gene, in all the genotypes. Out of the 22 candidate genes identified, we were able to evaluate the expression of 21 by utilizing transcriptome data. However, the leucine-rich repeat gene (Dioal.01G024400) exhibited no expression. In addition, we could associate the alternative alleles found to the expression for four candidate genes (*Dioal.13G053400*, *Dioal.04G130600*, *Dioal.02G061200*, *Dioal.10G014800*, *Dioal.13G093500*) (Supplemental Fig. 11).

Hereafter, we present in more details five key genes significantly associated with textural traits, starch content and colour including two for which the expression was correlated to the alleles modifications.

Pectin methylesterase (*Dioal.04G130600.1*), was associated with gumminess and total area and found under selection (Fig. 4A). A tuber pectin methyl esterase activity has been identified as a potential factor impacting cooked potato textural properties [18]. The SNP S4_20715506 (C/T) located in the single exon of *Dioal.04G130600.1* (Fig. 4A), the change result on the loss of the initial methionine. We observed that the C-allele at S4_20715506 decreases gumminess (Fig. 4A) and total area (Supplemental Fig. 10). None of the accessions used for transcriptome analysis had the T-allele (Fig. 4B), hence we were unable to evaluate the effect of this polymorphism on gene expression patterns between these two haplotypes. Nonetheless, our results suggest that allelic variation in *Dioal.04G130600.1* could affect the enzyme activity, leading to varying textural properties of boiled yam.

Beta-glucosidase, found associated with hardness (Fig. 4C), is a cellulase enzyme playing a part in metabolism of cell wall polysaccharides [48]. The alternative C-allele at the qH13.2 had a strong effect on hardness (62%) by modifying the splice donor on position 837 in the gene *Dioal.13G093500* (Table 1). The genotypes CRB47, Roujol49, and Roujol75 which are homozygous for the G-allele showed an overall higher expression than the genotypes with the C-allele (CRB96, Roujol62, and Roujol9) (Fig. 4D; Supplemental Fig. 11).

Among the three QTNs associated to starch content (Table 1), the 1,3-beta-glucan-synthase (*Dioal.10G014800*) had the highest effect (Fig. 4E) and was also found in an outlier region by our *Fst* analysis (Supplemental Table 5). The genotypes harbouring the G-allele had a premature stop codon instead of a glycine. They had 14% less SC compared to the genotypes with the T-allele. We speculated that the gain of a stop codon results in a non-functional or altered protein which affects the starch content.

**Fig. 5:**
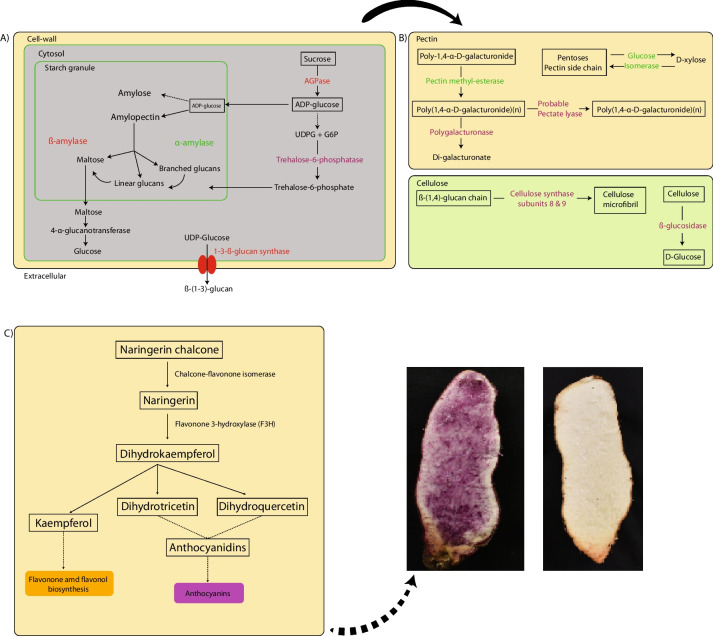
Schema of the predicted genes found by candidate association analysis. By candidate association analysis within the pentose and glucuronate interconversions, in green, starch and sucrose metabolism pathway, in red, flavonoids biosynthesis, in black. In purple genes under selection identified by Fst scan. Combined colours are genes found using both approaches

Lastly, within the flavonoid biosynthesis pathway, we identified two QTNs qHI4.1 and qBI19.1 for Brown index (BI) and HUE index (HI), respectively (Table 1). The gene *Dioal.19G083800.1* encodes for flavanone 3-hydroxylase (F3H). It catalyses the 3-beta-hydroxylation of 2S-flavanones to 2R,3R-dihydroflavonols which are intermediates in the biosynthesis of major pigments (flavonols, anthocyanidins, and proanthocyanidins) [49]. *Dioal.19G083800.1* contains SNP S19_17029453 (G/C) in the last exon, with the G-allele leading to a premature stop codon, a truncated protein and likely a non-functional enzyme (Fig. 4G). The genotypes with the G-allele at SNP S19_17029453 tend to have brown colour in contrast to the genotypes with the C-allele displaying mostly whitish tuber flesh (Fig. 5). Thus, this mutation may block the formation of flavonoid compounds and redirect the phenylpropanoid pathway to the formation of other brownish compounds like oxidized phenolic acids (Fig. 5). Expression pattern of *Dioal.19G083800* between two contrasting accessions with G/C alleles showed that it was expressed nearly 20 times higher in heterozygous (Roujol75; C/G) than in homozygous (Roujol62; G/G) genotypes (Fig. 4H). Overall, these results suggest that a functional mutation in *Dioal.19G083800* partly modulates colour formation in yam tuber.

Another key gene detected for colour variation (purple and non-purple yam varieties) was *Dioal.04G073400*, encoding an UDP-glucosyltransferase (UGT). Anthocyanins are unstable water-soluble pigments. UGTs are key enzymes that stabilize anthocyanidin by attaching sugar moieties to the anthocyanin aglycone [50]. In this study, the T-allele at the SNP S4_16031453 leads to a stop codon in the middle of *Dioal.04G073400* (Fig. 4I), which will probably result in a non-functional enzyme. Intriguingly, all accessions with the T-allele displayed purple tuber (very low HI) (Fig. 4I). It is possible that the non-functionality of the enzyme producing uncoloured flavanones redirect the pathway to anthocyanins biosynthesis resulting in the purple colour of the tubers (Fig. 5).

Altogether, the candidate gene association analysis showed that variants in several genes affect the functions and potentially the expression of key structural genes involved in the regulation of yam tuber quality traits. Interestingly, only 38% of the genes found in this analysis were retrieved from the keyword annotation, while the remaining were retrieved by comparative genomics.

## Discussion

In this study, the use of comparative genomics and genome sequence analysis of 127 *Dioscorea alata* accessions enabled the identification of candidate genes in the main metabolic pathways involved in yam tuber quality. The functional annotation of the *D. alata* genome remains suboptimal since there are few genomic data available for this crop [13]. Only recently a complete *D. alata* genome reference was published [8]. Therefore, the comparative genomics combined with keyword search used in this study, allowed the identification of novel genes associated with the three metabolic pathways targeted, pentose and glucuronate interconversions, starch and sucrose metabolism, and flavonoid biosynthesis pathways. We found more paralogs for each metabolic pathway, even if the number of different ECs remained the same. We annotated orthologous groups using the conserved domains, which facilitated the correction of incomplete or outdated gene assignments, such as for Malectin-like genes (MLD). The MLD genes, frequently erroneously described as Receptor-Like Kinases, can bind to pectin, with a preference for more highly methyl esterified pectin, causing a loosening in the cell wall [50]. In addition, based on the orthology analysis we found gene structure errors, such as for OG0082990 and OG0083049 (Supplemental Data 1), which calls for manual curation of important gene families to further improve the functional annotation of *D. alata* genome.

We identified three main gene pools in the admixture analysis, one composed mainly of Pacific representatives and, to a lesser extent, African and Caribbean genotypes, one composed mainly of African and Caribbean representatives, and the last of Asian representatives. This is consistent with our previous work, which highlighted an early divergence between the Asian and Pacific gene pools. The Indian gene pool later split from that of Asia and spread westwards to Africa and later to America. The Pacific gene pool spread eastwards. Over time, human translocation led to tuber exchanges between the different continents [5]. Consequently, quality traits have most likely been shaped by the evolution of the greater yam, due to environmental conditions, human selection and traditions.

We found that various gene ontology categories were enriched from the genes under selection between the three identified genetic groups. The most enriched pathways under selection were transmembrane transport, protein phosphorylation, response to wounding, hormone signalling, lignin biosynthetic process, known to play important physiological roles in defence response during plant-stress interactions [51]. The enrichment of gene ontology categories associated with stress response suggest that the selected genotypes from this study were selected not only by their tuber qualities, but also by their stress response. Their ability to withstand these challenges is crucial to produce high-quality tubers. The focus on the three pathways mostly involved in tuber quality traits revealed key enzymes likely under selection: cellulose synthase, required for primary and secondary cell wall cellulose synthesis [52], pectate lyase and polygalactunorase known as pectin degrading enzymes, involved in the demethylesterification of homogalacturonans. We also identified genes involved in starch synthesis, nudix hydrolase, soluble starch synthase 3 and 1,3- beta-glucan synthase [53], these genes are responsible for starch synthesis in plants [54], confirming the importance of starch content in yam during farmer selection and most probably during domestication [55]. In addition, recent studies demonstrated that the manipulation of a 1,3-beta-glucan synthase gene in barley induced the starch and polysaccharide profiles in grains [56]. Genes for the pentose and glucuronate interconversion pathway were found under selection only between African and Asian genetic groups. This result deserves further investigation to understand the nature of specific or local drivers of selection (environment or human mediated) in this pathway. Five genes from the pectin and starch-sugar pathways were found in common between the *Fst* analysis and candidate gene association study, showing that the quality of yam tubers was also a factor of differentiation and selection during yam evolution.

The candidate gene association analysis enabled the identification of fourteen pectin and three starch candidate genes associated with texture, indicating the involvement of the cell wall and starch content in boiled yam texture [57]. Among the seventeen candidate genes, three have high effects on the phenotype. They are known as involved directly in the cell wall composition. Beta-glucosidase explains more than 60% of the hardness confirming it as a major QTN in boiled yam texture. Its role in cell wall thickness has been demonstrated in different crops such as potato [18] or barley, in which it participates in endosperm cell wall degradation during germination [58]. In cotton (*Gossypium hirsutum*), the overexpression of the *GhBG1A,* a gene encoding beta-glucosidase, repressed fiber length but promoted cellulose biosynthesis resulting in thicker fiber cell wall [59]. The four remaining predicted genes with high effect on texture parameters were involved in pectin pathway. Pectin methylesterase, found also under selection, encodes an enzyme which plays an important role in both pectin remodelling and disassembly and consequently in firming and softening of cell wall [60]. In our study, it has a negative effect on both total area and gumminess, indicating its involvement in pectin degradation during yam boiling, such as in cooked potato [18]. In cassava, its role in the root softening process during cassava retting was demonstrated in addition to pectin/pectate lyase and polygalacturonase genes [61]. These two last genes were found under selection in greater yam, but not associated with any trait investigated. Finally, xylose isomerase seems to play an important role in boiled yam texture, because of its association with gumminess and springiness, probably by catalysing the reversible isomerisation of pentoses such as arabinose, one of the components of pectin side chain [62].

Such as for cereals and other tuber crops, the total starch content of yams is a primary determinant of tuber quality [63]. Preferred mealy cooking yam cultivars had significantly higher starch content [57]. Among the three predicted genes associated with starch content, the 1,3-beta-glucan synthase had the highest effect. It is often considered to be a cellulase family member, and plays an important role in cellulose structure. In this study this gene family was found under selection and predicted to be associated with starch content. The (1,3;1,4)-beta-D-glucans are most abundant in walls of the cereals, specifically in the starchy endosperm of grain, where they can contribute up to 70% by weight of the cell walls in barley, rye, and oats [64]. Whether 1,3-beta-glucan synthase gene is related to starch content on yam or other tuberous starchy crops remains unclear.

The colour is a determinant key trait in yam varieties adoption. While in West Africa white colour is preferred, in the Pacific colourful plates are more appreciated. In our study the flavanone 3-hydroxylase (F3H) was found involved in brown index with a high effect. This gene is involved in the accumulation of catechins in tea plants (*Camelia sinensis* L.) [65]. In carnation flower (*Dianthus caryophyllus* L.) it has been found involved in colour and fragrance [66]. RNAi-Mediated silencing of the F3H confirmed that this gene is one of the key enzymes required for the biosynthesis of flavonoids in strawberry fruit [67]. The UDP-glucosyltransferase was found associated with purple colour confirming its role in colour formation in yam tuber. None of these genes were found through transcriptome analysis of two contrasting *D. alata* genotypes for tuber purple colour [68] or by a genome-wide association analysis [21]. These findings suggest the possibility of additional genes playing a role in determining tuber colour. This speculation arises from the fact that our study primarily concentrated on analysing SNPs found in coding regions and focused solely on genes associated with the three investigated pathways.

## Conclusion

In conclusion, quality is one of the main criteria selected during evolution and adaptation of greater yam, which is supported by the functional role of the genes identified in this study. We found 22 candidate genes, in pentose and glucuronate, sucrose and starch, and flavonoid biosynthesis pathways, associated to the three main attributes of boiled yam: texture, starch content and tuber colour. These traits are highly searched by consumers, and the present study will help advance yam breeding, allowing the focus on selecting genotypes with these favourable alleles. In addition, we were able to assess the expression profile of the candidate genes found in the association analysis, and associate the alternative alleles to a change in the expression values of four candidate genes. This is the first work that explores the different cell wall constituent genes and their effect on texture. Further validation of these results on a larger panel by performing a genome-wide association analysis combined with metabolomic and transcriptomic analyses will confirm the robustness of our approach. Validation of the identified genes and alleles will pave the way for favourable allele pyramiding in breeding programs. Moreover, the use of comparative genomics to complement a keyword search approach to retrieve genes from the three pathways has proved highly effective to retrieve new protein-coding genes, and to enrich genomic resources. This approach, applied to orphan crops for which few genomic resources were produced, will increase the information available in public databases, and help breeders to find key genes for important traits.

### Supplementary Information

**Supplementary Material 1. **

**Supplementary Material 2. **

## Data Availability

The Illumina HiSeq 3000 sequencing raw data and transcriptome data are available in the NCBI SRA (Sequence Read Archive), under the BioProject number: PRJNA918625. The phenotypic datasets are available from the corresponding author upon request.

## Abbreviations

AIC: Akaike Information Criterion
BI: Brown Index
CRB-PT: Centre de Ressources Biologiques-Plantes Tropicales
DNA: Deoxyribonucleic acid
EC: Enzyme codes
F3H: Flavanone 3-hydroxylase
Fst: Fixation Index
GFF: General Feature Format
GO: Gene Ontology
HI: HUE index
IITA: International Institute of Tropical Agriculture
MASL: Metres above sea level
MATAB: Mixed alkyl trimethylammonium bromide
MLD: Malectin-like receptor kinase gene
NIRS: Near infrared spectroscopy
OG: Orthology groups
PCA: Principal Component Analysis
PG: Polygalactunorase Gene
PME: Pectin Methylesterase Gene
PRC: Plant Resources Center
QTN: Quantitative Trait Nucleotides
RNA: Riboxynucleic Acid
SC: Starch Content
SNP: Single Nucleotide Polymorphism
UGT: UDP-glycosyltransferase
VCF: Variant calling File
