# Supplementary material for: Whole-genome sequencing and comparative genomics reveal candidate genes associated with quality traits in Dioscorea alata
Source: BMC Genomics. 2024 Mar 6;25:248. doi: 10.1186/s12864-024-10135-2 (PMC10916269; doi:10.1186/s12864-024-10135-2)
Supplement: Supplementary file 1 — Supplementary Material 1. [file 12864_2024_10135_MOESM1_ESM.docx]

**SUPPORTING INFORMATION**

Whole-genome sequencing and comparative genomics reveal candidate

genes associated with quality traits in *Dioscorea alata*

**Ana Paula Zotta Mota^1,2,3†^, Komivi Dossa^1,4†^, Mathieu Lechaudel^5,6^, Denis Cornet^1,2^, Pierre Mournet^1,2^, Sylvain Santoni^2^, David Lopez^1,2*^, Hana Chaïr^1,2*^**

1 CIRAD, UMR AGAP, F34398-Montpellier, France,

2 AGAP, Univ Montpellier, CIRAD, INRAe, Montpellier SupAgro,Montpellier, France,

3 Université Côte d’Azur, Institut Sophia Agrobiotech, INRAE, 06903 Sophia

Antipolis, France

4 CIRAD, UMR AGAP Institut, 97170 Petit Bourg, Guadeloupe, France.

5 UMR Qualisud, CIRAD, F97130-Capesterre-Belle-Eau, Guadeloupe, France

6 QualiSud, Université Montpellier, Institut Agro, CIRAD, Avignon Université,

Université de la Réunion, 34398 Montpellier, France

**†**These authors contributed equally to this work

*Corresponding authors: hana.chair@cirad.fr; [david.lopez@cirad.fr](mailto:david.lopez@cirad.fr)

LEGENDS:

**Supplemental Figure 1.** **Quality and coverage of mapping of genotypes against *D. alata* genome**

**Supplemental Figure 2.** **Number of SNP per condition**.

A) Each column colour indicates one condition per chromosome. Grey: number of SNPs found only in gene coding regions, identified on the gff information. Yellow: SNPs found in the diploid species (107 genotypes) and using the filters ("ld-window 50, maf 0.01, max alleles 2, min-alleles 2, thin 10, min-r2 0.1, max-missing 0.5, remove-indels"). Orange: Number of SNPs found on all genotypes, after filters ("DP > 10 < 200, QUAL > 30, max-missing 0.05"). Blue: total number of SNPs, with no filters applied. B) Percentage of each type of SNPs found in the variant calling.

**Supplemental Figure 3. Population structure of *D. alata* genotypes.** A) neighbour joining tree and B) Alluvial diagram depicting the assignment of each genotype to the cluster identified by Admixture at K=3, according to its geographical origin

**Supplemental Figure 4. Identification of the weighted Fst theoretical distribution.** A) Observed distribution of weighted Fst values. B) Akaike information criterion (AIC) values of tested theoretical distributions (i.e. normal, beta, gamma and Weibull). C) Observed (histogram) and Weibull theoretical (red line) densities distributions with cutoff (red dashed vertical line) corresponding to the top 5% most important weighted Fst values.

**Supplemental Figure 5.** **Phylogenetic tree of species.** Phylogenetic tree of the 45 plant species used on the orthology analysis.

**Supplementary Figure 6**. **Common groups among species used in orthology analysis**. Number of orthologous groups shared among the 45 plant species. Each dot indicates the species which share the orthologous groups. The bars represent the number of groups shared.

**Supplemental Figure 7.** **Metabolic pathway of pentose and glucuronate interconversions**.

The red squares represent the EC number found in *D. alata* using the keyword analysis, described on the main material and methods.

**Supplemental Figure 8.** **Metabolic pathway of starch and sucrose metabolism**.

The red squares represent the EC number found in *D. alata* using the keyword analysis, described on the main material and methods.

**Supplemental Figure 9.** **Metabolic pathway of flavonoid biosynthesis**.

The red squares represent the EC number found in *D. alata* using the keyword analysis, described on the main material and methods.

**Supplemental Figure 10.** **Phenotypic values for all traits analysed and their gene structure**. A) Boxplot of measures for each gene found on the GWAS analysis and their values for each allele. B) Percentage of each type of modification found in the polymorphism analysis of the candidate genes for the five traits. C) Gene structure and modification found for each allele. The black boxes indicate the exons, and the lines indicate the introns.

**Supplemental Figure 11: Gene expression of candidate genes.** Expression values of -log2 of TPM for each candidate gene, which quality trait the QTN is associated, and the allele for each genotype. The five genes described in detail in the results section, are highlighted with a black box. The genes confirming phenotype of alternative alleles with a difference in transcription, are indicated by red boxes.

**Supplemental note 1. Colour and discolouration phenotypic analysis**

**Supplemental note 2. Starch content phenotypic analysis**

**Supplemental note 3. Texture phenotypic analysis**

**Supplemental Figure 1.** **Quality and coverage of mapping of genotypes against *D. alata* genome**


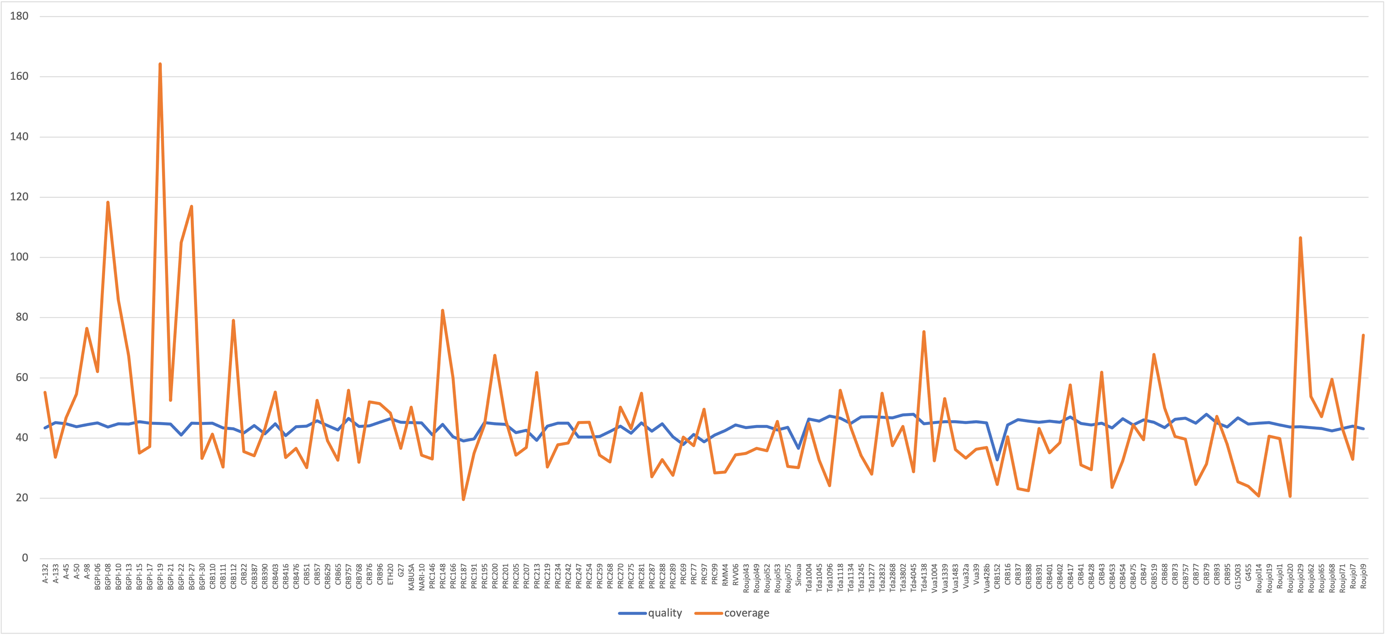


**Supplemental Figure 2.** **Number of SNP per condition**.

A) Each column colour indicates one condition per chromosome. Grey: number of SNPs found only in gene coding regions, identified on the gff information. Yellow: SNPs found in the diploid species (107 genotypes) and using the filters ("ld-window 50, maf 0.01, max alleles 2, min-alleles 2, thin 10, min-r2 0.1, max-missing 0.5, remove-indels"). Orange: Number of SNPs found on all genotypes, after filters ("DP > 10 < 200, QUAL > 30, max-missing 0.05"). Blue: total number of SNPs, with no filters applied.

B) Percentage of each type of SNPs found in the variant calling.

**Supplemental Figure 3. Population structure of *D. alata* genotypes.** A) neighbour joining tree and B) Alluvial diagram depicting the assignment of each genotype to the cluster identified by Admixture at K=3, according to its geographical origin


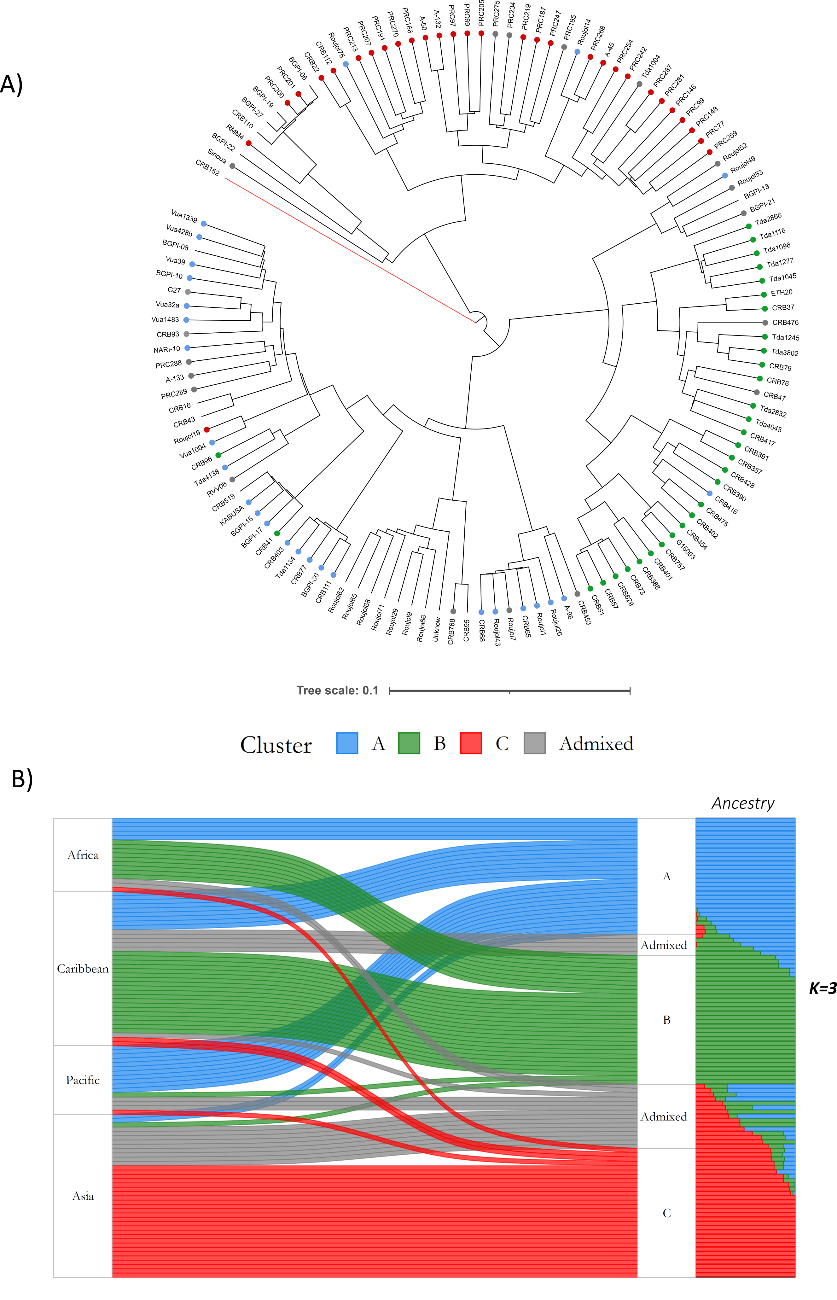


**Supplemental Figure 4. Identification of the weighted Fst theoretical distribution.** A) Observed distribution of weighted Fst values. B) Akaike information criterion (AIC) values of tested theoretical distributions (i.e. normal, beta, gamma and Weibull). C) Observed (histogram) and Weibull theoretical (red line) densities distributions with cutoff (red dashed vertical line) corresponding to the top 5% most important weighted Fst values.

**Supplemental Figure 5.** **Phylogenetic tree of species.** Phylogenetic tree of the 45 plant species used on the orthology analysis.

**Supplementary Figure 6**. **Common groups among species used in orthology analysis**. Number of orthologous groups shared among the 45 plant species. Each dot indicates the species which share the orthologous groups. The bars represent the number of groups shared.

**Supplemental Figure 7.** **Metabolic pathway of pentose and glucuronate interconversions**.

The red squares represent the EC number found in *D. alata* using the keyword analysis, described on the main material and methods.


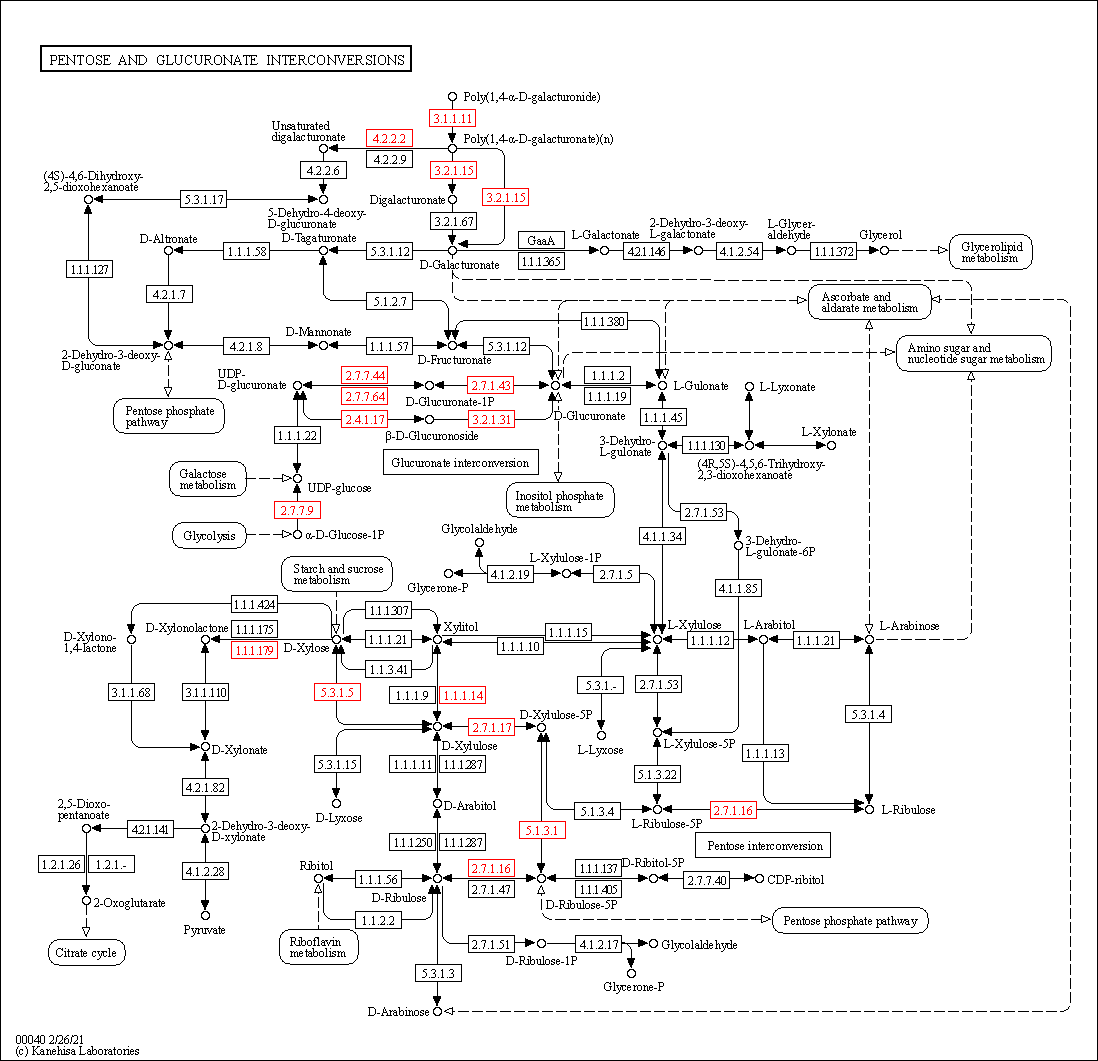


**Supplemental Figure 8.** **Metabolic pathway of starch and sucrose metabolism**.

The red squares represent the EC number found in *D. alata* using the keyword analysis, described on the main material and methods.


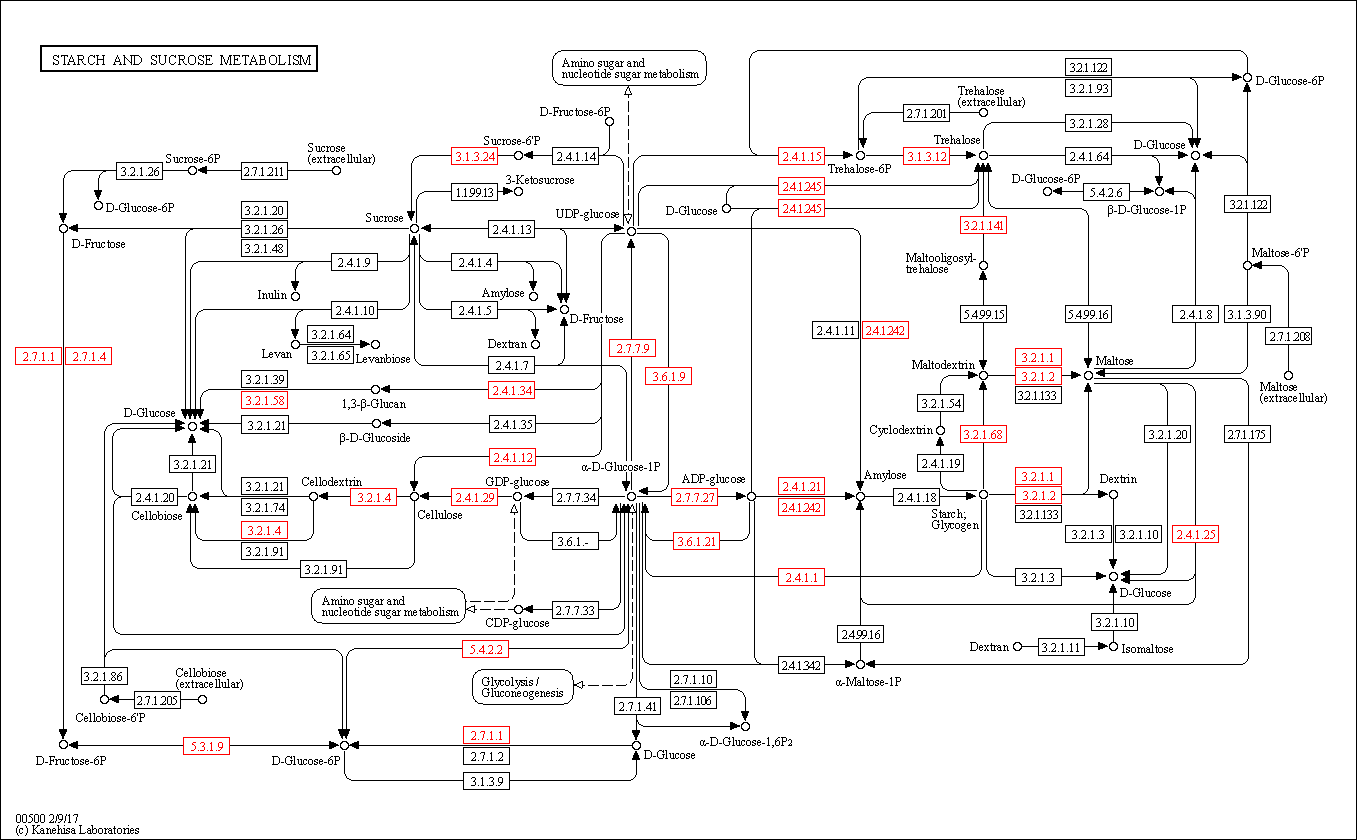


**Supplemental Figure 9.** **Metabolic pathway of flavonoid biosynthesis**.

The red squares represent the EC number found in *D. alata* using the keyword analysis, described on the main material and methods.


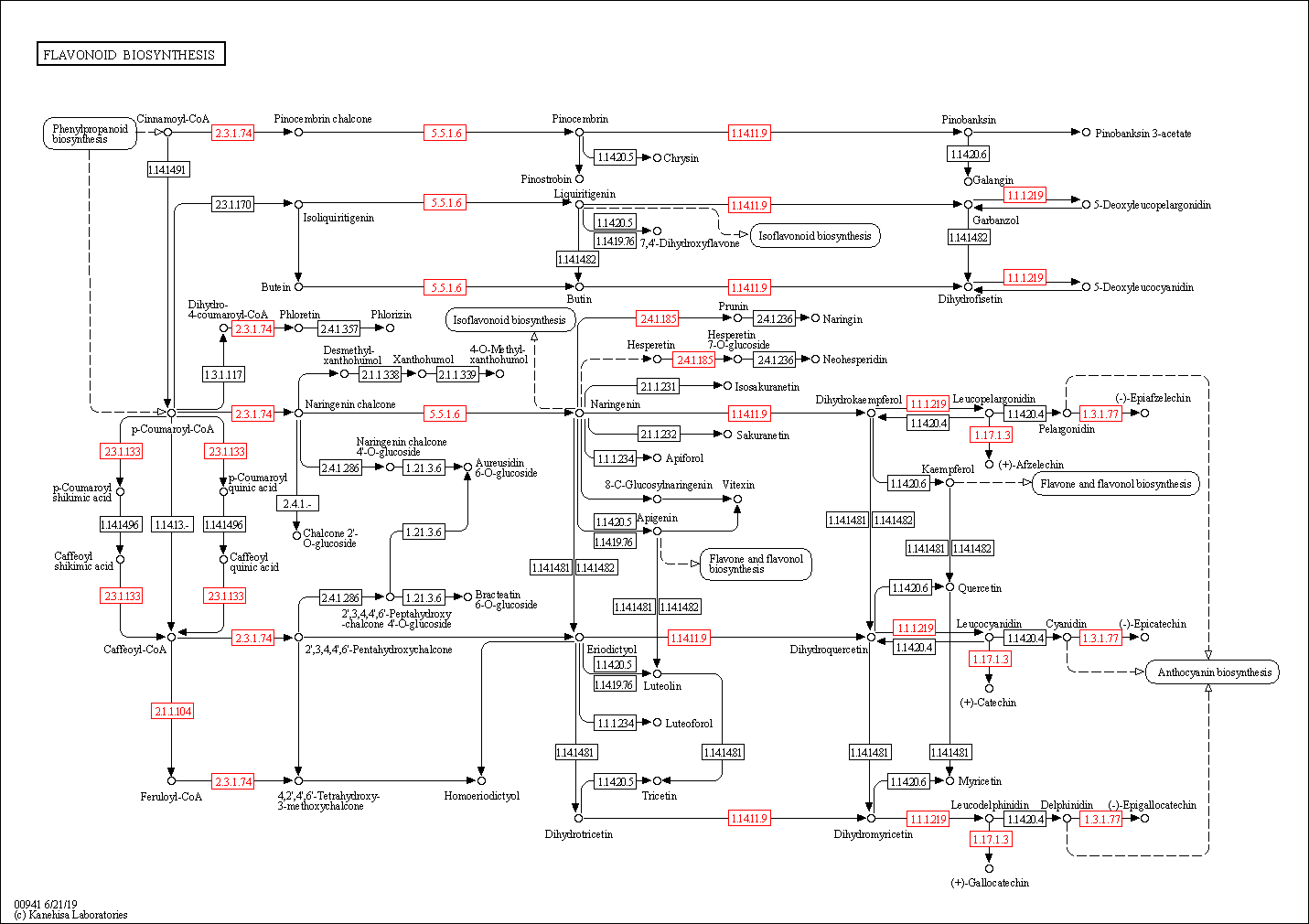


**Supplemental Figure 10.** **Phenotypic values for all traits analysed and their gene structure**.

A) Boxplot of measures for each gene found on the GWAS analysis and their values for each allele. B) Percentage of each type of modification found in the polymorphism analysis of the candidate genes for the five traits. C) Gene structure and modification found for each allele. The black boxes indicate the exons, and the lines indicate the introns.


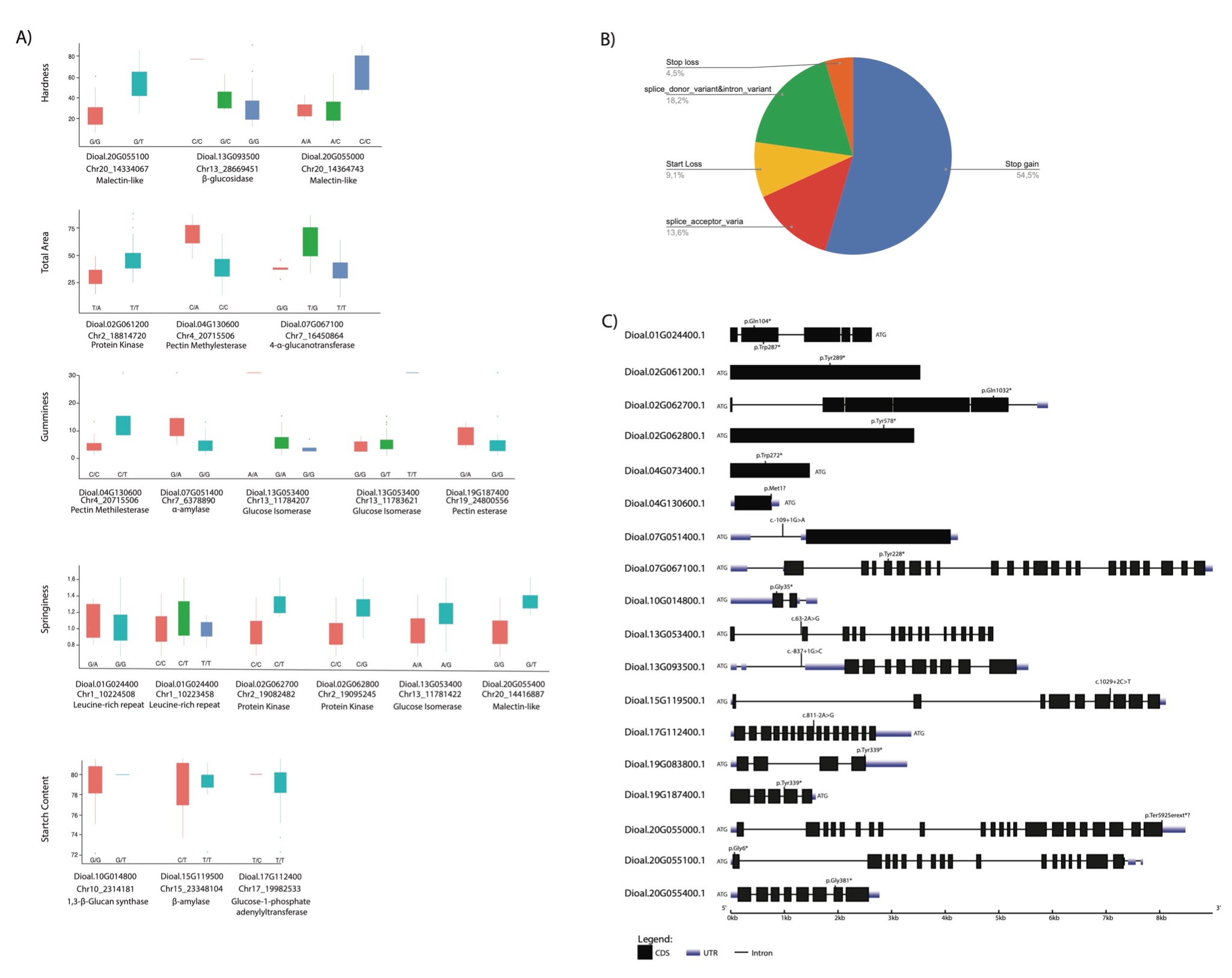


**Supplemental Figure 11: Gene expression of candidate genes.** Expression values of -log2 of TPM for each candidate gene, which quality trait the QTN is associated, and the allele for each genotype. The five genes described in detail in the results section, are highlighted with a black box. The genes confirming phenotype of alternative alleles with a difference in transcription, are indicated by red boxes.

**Supplemental Data 1: Orthologous groups obtained from Orthofinder analysis.**

<https://doi.org/10.18167/DVN1/A4OYRU>

**Supplemental note 1. Colour and discolouration phenotypic analysis**

Computerised image analysis techniques not only offer a methodology for measurement of uneven colouration on food product exhibiting irregular surfaces (Mendoza et al., 2006), but it can also be applied repeatedly over time in order to characterise discolouration. Image was collected on three tuber per genotype (45) and location (3) during the 2018 cropping season. Using a ceramic knife, tubers were cut longitudinally in order to capture radial and longitudinal gradient (REF) and were then directly placed on a black background.

Image acquisition was made using a Nikon D5600 equipped with an AF-S DX zoom Nikkor 18-55mm f/3.5-5.6 and a remote control. Camera was fixed perpendicularly above the tubers using an extension pole and a tripod. Light environment was controlled using two floodlight with tungsten halogen 3200°K bulbs of 500 watt each, placed on both side of tuber sample facing reflective umbrella (Interfit EZ-Lite 2-Light Kit). To follow discolouration in time, the camera intervalometer was set to take picture every 30 seconds for 15 minutes.

To test the repeatability of the system, a custom colour chart was placed on every picture. CIE L*a*b* true colour values of the custom colour chart were measured using a chromameter Minolta CR300, Processer DP 301with a D65 illuminant. These theoretical colour values were compared with observed colour values extracted from the picture. Acquired image were analysed using the Rvision library (Garnier and Muschelli, 2022) with R programming language (R Core Team and others, 2013). Colour patches of the custom colour chart were segmented using simple object detection algorithm based on defined shape, distance, and size. sRGB colour value were averaged for every patch on every picture and then converted into the CIE L*a*b* colour space. The colour difference between theoretical and observed colour were calculated using the $\Delta E_{2000}^{*}$ formula as in (Luo et al., 2001). An average colour difference value of 4.21 was found which can be considered as acceptable for complex images (Song and Luo, 2000).

Tuber segmentation was carried on based on colour thresholds applied to the first picture taken at time 0. Coordinates of tuber flesh pixels are then recorded and used to extract tuber pixels from background on the remaining 29 pictures of a time series. sRGB colour value are converted into CIE L*a*b* colour space to summarize colour information into useful colour indices. Different colour index was tested in order to characterize purple yam. Finally, the Hue was found to be discriminant within the studied diversity panel:

$$Hue=\frac{b^{*}}{a^{*}}$$

The lower the Hue, the more purple the tuber. Brownness (BI) index was calculated by:

$$x=\frac{\left( a^{*}+1.75L^{*} \right)}{\left( 5.645L^{*}+a^{*}-3.012b^{*} \right)}$$

$BI=\left[ 100\left( x-0.31 \right) \right]/0.172$ (Buera et al., 1986)

The browning index (BI) represents the purity of brown colour and is reported as an important parameter in processes where enzymatic or non-enzymatic browning takes place (Palou et al., 1999). High values indicate tubers with a brown colour.

**Supplemental note 2. Starch content phenotypic analysis**

Starch content was predicted using near infrared spectroscopy (NIRS). Reference values for starch were quantified using Ewers polarimetric protocol (ISO 10520). NIR spectroscopy analyses were carried out in the food processing laboratory of INRAE’s Tropical Animal Research Unit, UR143, in Guadeloupe (France). Two replicates of yam flour samples were scanned with a FOSS-NIRSystems model 6500 scanning monochromator (FOSS-NIRSystems, Silver Spring, MD, USA) equipped with an autocup. The spectroscopic procedures and data recording were conducted with ISIscan (TM) software (FOSS, Hillerød, Denmark). Each flour was placed in a small ring cup 36mm in diameter, and reflectance pectra from 400 to 2500nm were recorded at 2 nm intervals.

The model was calibrated using 2016 and 2017 data and validated on external independent 2018 dataset. Pretreatments, calibration and validation were carried out using python language (v3.6, https://www.python.org) with a Keras framework (v2.1.5, https://keras.io/) and a TensorFlow backend (v1.6.0, <https://www.tensorflow.org>). Twelve filters based on Haar transform, Gaussian derivatives, SVG, SNV, and different degrees of MSC were combined two by two leading up to 157 different pretreatments. No spectral outlier was removed. Calibration was done minimizing the mean square error.

Model calibration was done using a convolutional neural network (Ehounou et al., 2018; Vasseur et al., 2018). First, a data augmentation procedure was applied on the calibration data set: for each original sample, five synthetic spectra were generated using a combination of random transformations of the original spectra and added to the initial calibration data set. Then all pre-treatments were applied to this augmented dataset and the resulted spectra associated with the original ones. A convolutional neural network composed of three convolutional layers followed by two dense layers was fitted to the calibration data. In order to avoid overfitting, a batch normalization procedure/layer was applied between the first two convolutional layers and a dropout of 20% of features after the third layer. The model was calibrated using three-fold cross validation. To test the robustness of the model an independent validation was finally done using the validation set. The root mean square error and coefficient of determination of the validation step show fairly good performance (i.e. RMSE=1.86% and R²=0.77) allowing to use the model to predict starch content on 2019 samples.

**Supplemental note 3. Texture phenotypic analysis**

*Yam samples preparation and cooking for texture measures*

Three tubers per variety were sampled. Each tuber was divided, as far as possible, into three equal sections (proximal, distal, central) after having measured its length. After peeling and washing, the sections were cut as follows: for the proximal and distal parts, 1/10 and 3/10 at the bottom side of section length was removed and discarded while for the central part, 2/10 from both ends of section was removed and discarded, and thus 6/10 of each section length (as representative of each part) was suitable to cooking. Each usable/operational part (6/10) was used to produce three cubes of 23 mm edge for penetrometric measurements, and in the central section three additional cubes were sampled at the central section for TPA analysis. Each cube was steam-cooked up to 15 min, followed by a cooling time of 7 min, corresponding to a cube temperature of 45°C.

*Instrumental measurement of the texture of steam-cooked yam*

Instrumental parameters of the texture of steam-cooked yam were measured by puncture and by double-compression tests using the TAX-TPlus texture analyser (Stable Micro Systems, Ltd., Surrey, UK). All textural measurements were made at a sample temperature set at 45°C. In a preliminary test, the temperature during sample cooling was monitored by the Almemo 26-908A data logger equipped with a K-type thermocouple (diameter 1 mm) inserted into the geometrical core of the sample to determine the time being necessary to the decrease of the sample temperature at 45°C.

For the puncture test, a 5-mm-diameter cylindrical metal borer (surface area ~ 20 mm^2^) penetrated the yam cube at a constant speed (1 mm s^−1^) to a depth of 15 mm. The force applied during the displacement of the probe was recorded. Two parameters were calculated from the puncture test have been achieved: hardness (N) and total area (N s), which reflects the total force required to penetrate the sample at a constant speed.

For the double-compression test performed using Texture Profile Analysis (TPA), two compression cycles, each corresponding to 25% of sample strain, were performed at a constant crosshead speed of 1 mm s^−1^, using a probe 60 mm in diameter. Force-time curves were recorded by the software of the instrument. Different TPA parameters were computed from the force-time curve: hardness (N) refers to the peak resistive force during the first compression cycle, cohesiveness (with no unit) is represented by the ratio of the area under the second bite to the area under the first bite *ie* how well a boiled yam retains its shape between the 1st and 2nd compression cycle, gumminess is the energy required to disintegrate a semi-solid food to a swallowable state. It is the product of the semi-solid food's hardness multiplied by its cohesiveness. Finally, springiness (with no unit) represented by the ratio of the distance travelled during the first descent to the distance travelled during the second descent of the probe *ie* a measure of how well a product physically springs back after being deformed during initial compression.

References

**Buera, M. P., Lozano, R., and Petriella, C.** (1986). Definition of colour in the non enzymatic browning process. *Farbe* **32**:318–322.

**Ehounou, A. E., Kouakou, A. M., N’zi, J. C., Dibi, K. E. B., Bakayoko, Y., Essis, B. S., Boni, N., Maledon, E., Asfaw, A., Adebola, P., et al.** (2018). Production of Hybrid Seeds by Intraspecific Crossing in Yam Dioscorea alata L **8**:11.

**Garnier, S., and Muschelli, J.** (2022). *Rvision - A computer vision library for R*.

**Luo, M. R., Cui, G., and Rigg, B.** (2001). The development of the CIE 2000 colour-difference formula: CIEDE2000. *Color Res. Appl.* **26**:340–350.

**Mendoza, F., Dejmek, P., and Aguilera, J. M.** (2006). Calibrated color measurements of agricultural foods using image analysis. *Postharvest Biol. Technol.* **41**:285–295.

**Palou, E., Lopez-Malo, A., Barbosa-Canovas, G. V., Welti-Chanes, J., and Swanson, B. G.** (1999). Polyphenoloxidase Activity and Color of Blanched and High Hydrostatic Pressure Treated Banana Puree. *J. Food Sci.* **64**:42–45.

**R Core Team, R. and others** (2013). R: A language and environment for statistical computing Advance Access published 2013.

**Song, T., and Luo, R.** (2000). Testing color-difference formulae on complex images using a CRT monitor. In *Color and Imaging Conference*, pp. 44–48. Society for Imaging Science and Technology.

**Vasseur, F., Exposito-Alonso, M., Ayala-Garay, O. J., Wang, G., Enquist, B. J., Vile, D., Violle, C., and Weigel, D.** (2018). Adaptive diversification of growth allometry in the plant Arabidopsis thaliana. *Proc. Natl. Acad. Sci.* **115**:3416–3421.
