# Supplementary material for: Whole-genome sequencing and comparative genomics reveal candidate genes associated with quality traits in Dioscorea alata
Source: BMC Genomics. 2024 Mar 6;25:248. doi: 10.1186/s12864-024-10135-2 (PMC10916269; doi:10.1186/s12864-024-10135-2)
Supplement: Supplementary file 2 — Supplementary Material 2. [file 12864_2024_10135_MOESM2_ESM.docx]

**Supplementary Table 1** : Description of the sampling material for whole genome resequencing, transcriptome and GWAS analyses. Details on number of reads after sequencing, coverage, quality and number of SNP after filtering are provided for each genotype.

**Genotype Species Country Continent Ploidy Number of reads**

**from sequencing**

**Quality Coverage Number of SNP**

**after filtering**

**GWAS Transcriptome**

| A-132 | *D. alata* | Japan | Asia | 2 | 95766879 | 43,39 | 55,18 | 10299268 |  |
| --- | --- | --- | --- | --- | --- | --- | --- | --- | --- |
| A-133 | *D. alata* | Japan | Asia | 2 | 56550246 | 45,17 | 33,66 | 9400697 |  |
| A-45 | *D. alata* | Japan | Asia | 2 | 79152424 | 44,76 | 46,78 | 9978431 |  |
| A-50 | *D. alata* | Japan | Asia | 2 | 92603157 | 43,81 | 54,62 | 10358873 |  |
| A-98 | *D. alata* | Japan | Asia | 2 | 129711754 | 44,46 | 76,45 | 10191143 |  |
| BGPI-06 | *D. alata* | Haiti | Caribbean | 3 | 106081870 | 45,03 | 62,1 | 11307106 |  |
| BGPI-08 | *D. alata* | Vanuatu | Pacific | 4 | 205890368 | 43,71 | 118,38 | 14421690 |  |
| BGPI-10 | *D. alata* | Vanuatu | Pacific | 2 | 145266094 | 44,73 | 86,03 | 10563930 |  |
| BGPI-13 | *D. alata* | India | Asia | 3 | 113363112 | 44,69 | 67,33 | 9518329 |  |
| BGPI-15 | *D. alata* | Benin | Africa | 2 | 58911001 | 45,44 | 34,99 | 9075778 |  |
| BGPI-17 | *D. alata* | Ghana | Africa | 2 | 68765110 | 44,95 | 37,22 | 9250710 |  |
| BGPI-19 | *D. alata* | Madagascar | Africa | 3 | 279617423 | 44,84 | 164,34 | 11934631 |  |
| BGPI-21 | *D. alata* | India | Asia | 2 | 89157815 | 44,71 | 52,58 | 9382957 |  |
| BGPI-22 | *D. alata* | Madagascar | Africa | 4 | 184079473 | 41,02 | 104,95 | 19093920 |  |
| BGPI-27 | *D. alata* | Vanuatu | Pacific | 4 | 197470219 | 44,93 | 117,02 | 11761110 |  |
| BGPI-30 | *D. alata* | Cuba | Caribbean | 2 | 56074243 | 44,85 | 33,29 | 9098992 |  |
| CRB110 | *D. alata* | Haïti | Caribbean | 3 | 70192390 | 45 | 41,3 | 10902744 |  |
| CRB111 | *D. alata* | Guadeloupe | Caribbean | 2 | 51228076 | 43,41 | 30,39 | 8913816 |  |
| CRB112 | *D. alata* | Puerto Rico | Caribbean | 2 | 135039090 | 43,14 | 79,09 | 9884631 | GWAS |
| CRB152 | *D. rotundata* | Benin | Africa | 2 | 61233614 | 32,73 | 24,61 | 16085332 |  |
| CRB16 | *D. alata* | Nouvelle Calédonie | Pacific | 3 | 70071343 | 44,41 | 40,4 | 11089014 | GWAS |
| CRB22 | *D. alata* | Puerto Rico | Caribbean | 2 | 60700447 | 41,74 | 35,53 | 8466670 | GWAS |
| CRB37 | *D. alata* | Nouvelle Calédonie | Pacific | 2 | 39823042 | 46,11 | 23,21 | 7284724 | GWAS |
| CRB387 | *D. alata* | Guadeloupe | Caribbean | 2 | 59367771 | 44,17 | 34,14 | 7926715 | GWAS |
| CRB388 | *D. alata* | Guadeloupe | Caribbean | 2 | 38441129 | 45,7 | 22,55 | 7307228 | GWAS |
| CRB390 | *D. alata* | Guadeloupe | Caribbean | 2 | 74524936 | 41,46 | 43,05 | 7339785 | GWAS |
| CRB391 | *D. alata* | Guadeloupe | Caribbean | 2 | 74235036 | 45,3 | 43,18 | 7979661 |  |
| CRB401 | *D. alata* | Guadeloupe | Caribbean | 2 | 59351866 | 45,68 | 35,15 | 8196100 | GWAS |
| CRB402 | *D. alata* | Guadeloupe | Caribbean | 2 | 64918686 | 45,22 | 38,5 | 7924979 | GWAS |
| CRB403 | *D. alata* | Guadeloupe | Caribbean | 2 | 93934429 | 44,76 | 55,3 | 9566965 | GWAS |
| CRB41 | *D. alata* | Puerto Rico | Caribbean | 2 | 54111249 | 44,85 | 31,09 | 7476266 | GWAS |

| CRB416 | *D. alata* | Guadeloupe | Caribbean | 2 | 56420403 | 40,88 | 33,52 | 7665225 | GWAS |  |
| --- | --- | --- | --- | --- | --- | --- | --- | --- | --- | --- |
| CRB417 | *D. alata* | Guadeloupe | Caribbean | 2 | 96773097 | 47,03 | 57,66 | 8756798 | GWAS |  |
| CRB428 | *D. alata* | Guadeloupe | Caribbean | 2 | 50817028 | 44,38 | 29,5 | 8144078 | GWAS |  |
| CRB43 | *D. alata* | Nouvelle Calédonie | Pacific | 3 | 106128081 | 44,98 | 61,93 | 11720008 | GWAS |  |
| CRB453 | *D. alata* | Guadeloupe | Caribbean | 2 | 41479909 | 43,39 | 23,59 | 7826660 | GWAS |  |
| CRB454 | *D. alata* | Puerto Rico | Caribbean | 2 | 54949339 | 46,49 | 32,43 | 7411194 | GWAS |  |
| CRB47 | *D. alata* | Haiti | Caribbean | 2 | 66409430 | 46,07 | 39,45 | 8610506 |  | Transcriptome |
| CRB475 | *D. alata* | Guadeloupe | Caribbean | 2 | 76717055 | 44,39 | 44,42 | 7856422 | GWAS |  |
| CRB476 | *D. alata* | Saint Vincent | Caribbean | 2 | 62522347 | 43,74 | 36,61 | 8522483 | GWAS |  |
| CRB51 | *D. alata* | Puerto Rico | Caribbean | 2 | 51797448 | 44 | 30,2 | 9509097 | GWAS |  |
| CRB519 | *D. trifida* | French Guiana | Caribbean | 4 | 115371743 | 45,27 | 67,82 | 8235552 |  |  |
| CRB57 | *D. alata* | Brazil | Caribbean | 2 | 88707889 | 45,78 | 52,57 | 8903156 |  |  |
| CRB629 | *D. alata* | Guadeloupe | Caribbean | 2 | 66940088 | 44,21 | 39,16 | 8641229 | GWAS |  |
| CRB65 | *D. alata* | Puerto Rico | Caribbean | 2 | 56801675 | 42,71 | 32,7 | 9689719 | GWAS |  |
| CRB68 | *D. alata* | Puerto Rico | Caribbean | 2 | 86393195 | 43,49 | 49,99 | 10085360 | GWAS |  |
| CRB73 | *D. alata* | Guadeloupe | Caribbean | 2 | 68598201 | 46,22 | 40,5 | 8622365 | GWAS |  |
| CRB757 | *D. alata* | Guadeloupe | Caribbean | 2 | 93815088 | 46,56 | 55,91 | 7750770 | GWAS |  |
| CRB76 | *D. alata* | Puerto Rico | Caribbean | 2 | 88651839 | 44,07 | 52,03 | 12689412 |  |  |
| CRB768 | *D. alata* | Vanuatu | Pacific | 2 | 54284890 | 43,86 | 31,93 | 7808440 |  |  |
| CRB77 | *D. alata* | Cote d'Ivoire | Africa | 2 | 41762456 | 44,98 | 24,6 | 8482498 |  |  |
| CRB79 | *D. alata* | Guadeloupe | Caribbean | 2 | 53313818 | 47,9 | 31,39 | 6776332 | GWAS |  |
| CRB93 | *D. alata* | Barbados | Caribbean | 2 | 80979181 | 45,05 | 47,2 | 9236779 | GWAS |  |
| CRB95 | *D. alata* | Nouvelle Calédonie | Pacific | 3 | 65562692 | 43,67 | 37,88 | 12537473 | GWAS |  |
| CRB96 | *D. alata* | Puerto Rico | Caribbean | 2 | 87956030 | 45,26 | 51,42 | 9639635 | GWAS | Transcriptome |
| ETH20 | *D. alata* | Sri Lanka | Asia | 2 | 82663135 | 46,43 | 48,34 | 8386906 |  |  |
| G15003 | *D. alata* | Guadeloupe | Caribbean | 2 | 42633316 | 46,72 | 25,44 | 7358176 | GWAS |  |
| G27 | *D. alata* | Guadeloupe | Caribbean | 2 | 62634350 | 45,29 | 36,65 | 10944249 |  |  |
| KABUSA | *D. alata* | Guadeloupe | Caribbean | 2 | 85455342 | 45,17 | 50,25 | 9364302 |  |  |
| NARI-10 | *D. alata* | Papua New Guinea | Pacific | 2 | 58477009 | 45,07 | 34,36 | 9459790 |  |  |
| PRC146 | *D. alata* | Vietnam | Asia | 2 | 58272456 | 41,17 | 33,03 | 8896573 |  |  |
| PRC148 | *D. alata* | Vietnam | Asia | 2 | 140490051 | 44,62 | 82,48 | 10807327 |  |  |
| PRC166 | *D. alata* | Vietnam | Asia | 2 | 105373827 | 40,4 | 59,98 | 10051492 |  |  |
| PRC187 | *D. alata* | Vietnam | Asia | 2 | 33927062 | 39,06 | 19,57 | 8493993 |  |  |
| PRC191 | *D. alata* | Vietnam | Asia | 2 | 60085115 | 39,77 | 35,07 | 9173819 |  |  |
| PRC195 | *D. alata* | Vietnam | Asia | 2 | 76877000 | 45,12 | 44,36 | 10022922 |  |  |

| PRC200 | *D. alata* | Vietnam | Asia | 2 | 114725292 | 44,81 | 67,46 | 11361858 |  |
| --- | --- | --- | --- | --- | --- | --- | --- | --- | --- |
| PRC201 | *D. alata* | Vietnam | Asia | 2 | 79072162 | 44,58 | 46,23 | 10932099 |  |
| PRC205 | *D. alata* | Vietnam | Asia | 2 | 60435797 | 41,77 | 34,29 | 9622106 |  |
| PRC207 | *D. alata* | Vietnam | Asia | 2 | 63587344 | 42,62 | 36,93 | 9288711 |  |
| PRC213 | *D. alata* | Vietnam | Asia | 2 | 104720414 | 39,28 | 61,78 | 10524234 |  |
| PRC219 | *D. alata* | Vietnam | Asia | 2 | 55980420 | 44,03 | 30,42 | 9795033 |  |
| PRC234 | *D. alata* | Vietnam | Asia | 2 | 64034853 | 44,98 | 37,75 | 9259424 |  |
| PRC242 | *D. alata* | Vietnam | Asia | 2 | 65358626 | 44,94 | 38,35 | 9828187 |  |
| PRC247 | *D. alata* | Vietnam | Asia | 2 | 77157149 | 40,37 | 45,13 | 9681268 |  |
| PRC254 | *D. alata* | Vietnam | Asia | 2 | 77387289 | 40,35 | 45,24 | 9644953 |  |
| PRC259 | *D. alata* | Vietnam | Asia | 2 | 59726975 | 40,54 | 34,31 | 9019531 |  |
| PRC268 | *D. alata* | Vietnam | Asia | 2 | 54291758 | 42,24 | 32,08 | 9495200 |  |
| PRC270 | *D. alata* | Vietnam | Asia | 2 | 85353141 | 43,99 | 50,26 | 10001804 |  |
| PRC275 | *D. alata* | Vietnam | Asia | 2 | 73450109 | 41,61 | 43,22 | 9253254 |  |
| PRC281 | *D. alata* | Vietnam | Asia | 2 | 94636781 | 45,1 | 54,93 | 10198556 |  |
| PRC287 | *D. alata* | Vietnam | Asia | 2 | 46181720 | 42,34 | 27,19 | 9191874 |  |
| PRC288 | *D. alata* | Vietnam | Asia | 2 | 55820638 | 44,79 | 32,88 | 9251287 |  |
| PRC289 | *D. alata* | Vietnam | Asia | 2 | 47165311 | 40,48 | 27,69 | 8353053 |  |
| PRC69 | *D. alata* | Vietnam | Asia | 2 | 71743350 | 37,93 | 40,37 | 9604151 |  |
| PRC77 | *D. alata* | Vietnam | Asia | 2 | 63926528 | 41,26 | 37,52 | 8902212 |  |
| PRC97 | *D. alata* | Vietnam | Asia | 2 | 85222097 | 38,73 | 49,6 | 9549891 |  |
| PRC99 | *D. alata* | Vietnam | Asia | 2 | 50524744 | 40,99 | 28,48 | 8943031 |  |
| RMM4 | *D. alata* | Madagascar | Africa | 2 | 49288382 | 42,54 | 28,73 | 15573625 |  |
| Roujol1 | *D. alata* | Vanuatu | Pacific | 2 | 68182612 | 44,39 | 39,81 | 8900525 | GWAS |
| Roujol14 | *D. alata* | Vanuatu | Pacific | 2 | 35694479 | 44,96 | 20,8 | 8820192 |  |
| Roujol19 | *D. alata* | Vanuatu | Pacific | 2 | 69457842 | 45,17 | 40,65 | 9634263 | GWAS |
| Roujol20 | *D. alata* | Vanuatu | Pacific | 2 | 36415735 | 43,66 | 20,67 | 9584396 |  |
| Roujol29 | *D. alata* | Vanuatu | Pacific | 4 | 185487556 | 43,75 | 106,58 | 10383660 | GWAS |
| Roujol43 | *D. alata* | Guadeloupe | Caribbean | 2 | 60314605 | 43,49 | 34,89 | 12887428 | GWAS |
| Roujol49 | *D. alata* | India | Asia | 2 | 65047088 | 43,91 | 36,63 | 9732324 | GWAS Transcriptome |
| Roujol52 | *D. alata* | India | Asia | 2 | 61759395 | 43,87 | 35,8 | 9279504 |  |
| Roujol53 | *D. alata* | India | Asia | 2 | 77855784 | 42,67 | 45,56 | 9452744 | GWAS |
| Roujol62 | *D. alata* | Guadeloupe | Caribbean | 4 | 92335169 | 43,47 | 53,85 | 10424875 | GWAS Transcriptome |
| Roujol65 | *D. alata* | Guadeloupe | Caribbean | 4 | 82408582 | 43,2 | 47,13 | 13659734 | GWAS |
| Roujol66 | *D. alata* | Guadeloupe | Caribbean | 4 | 169830971 | 44,42 | 45,96 | 13213577 | GWAS |

| Roujol68 | *D. alata* | Guadeloupe | Caribbean | 4 | 102963745 | 42,4 | 59,52 | 13235923 | GWAS |  |
| --- | --- | --- | --- | --- | --- | --- | --- | --- | --- | --- |
| Roujol7 | *D. alata* | Vanuatu | Pacific | 2 | 56281989 | 43,98 | 32,92 | 14088626 | GWAS |  |
| Roujol71 | *D. alata* | Guadeloupe | Caribbean | 4 | 75874947 | 43,15 | 43,27 | 11771177 | GWAS |  |
| Roujol75 | *D. alata* | Guadeloupe | Caribbean | 2 | 53462245 | 43,6 | 30,57 | 9549894 | GWAS | Transcriptome |
| Roujol9 | *D. alata* | Vanuatu | Pacific | 4 | 128585712 | 43,1 | 74,23 | 10957820 | GWAS | Transcriptome |
| RVV06 | *D. alata* | Madagascar | Africa | 2 | 59913430 | 44,41 | 34,48 | 12938060 |  |  |
| Sinoua | *D. alata* | Vanuatu | Pacific | 2 | 56592894 | 36,58 | 30,21 | 19723443 | GWAS |  |
| Tda1004 | *D. alata* | Togo | Africa | 2 | 75236997 | 46,36 | 44,85 | 8433750 |  |  |
| Tda1045 | *D. alata* | Togo | Africa | 2 | 54932519 | 45,69 | 32,64 | 8152304 |  |  |
| Tda1096 | *D. alata* | Togo | Africa | 2 | 41235078 | 47,28 | 24,2 | 6285654 |  |  |
| Tda1118 | *D. alata* | Togo | Africa | 2 | 93286254 | 46,6 | 55,91 | 7832848 |  |  |
| Tda1134 | *D. alata* | Togo | Africa | 2 | 74807248 | 44,82 | 43,73 | 9315886 |  |  |
| Tda1245 | *D. alata* | Togo | Africa | 2 | 57815523 | 47,03 | 34,23 | 7160396 |  |  |
| Tda1277 | *D. alata* | Benin | Africa | 2 | 47069522 | 47,13 | 28,04 | 7318337 |  |  |
| Tda2832 | *D. alata* | Ghana | Africa | 2 | 93596436 | 46,96 | 54,94 | 6920696 |  |  |
| Tda2868 | *D. alata* | Togo | Africa | 2 | 63479745 | 46,78 | 37,53 | 7353034 |  |  |
| Tda3802 | *D. alata* | Cote d'Ivoire | Africa | 2 | 73402825 | 47,75 | 43,86 | 7138215 |  |  |
| Tda4045 | *D. alata* | Benin | Africa | 2 | 48646979 | 47,87 | 28,81 | 6219666 |  |  |
| Tda4138 | *D. alata* | Sierra Leone | Africa | 2 | 127601507 | 44,74 | 75,33 | 10062013 |  |  |
| Unknow | *D. alata* | Guadeloupe | Caribbean | 4 | 53375336 | 14,66 | 43,19 | 8539364 |  |  |
| Vua1004 | *D. alata* | Vanuatu | Pacific | 2 | 54817584 | 45,12 | 32,51 | 9444318 |  |  |
| Vua1339 | *D. alata* | Vanuatu | Pacific | 2 | 89392345 | 45,47 | 53,15 | 9112370 |  |  |
| Vua1483 | *D. alata* | Vanuatu | Pacific | 2 | 61464154 | 45,41 | 36,23 | 9349818 |  |  |
| Vua32a | *D. alata* | Vanuatu | Pacific | 2 | 56034208 | 45,2 | 33,32 | 9543158 |  |  |
| Vua39 | *D. alata* | Vanuatu | Pacific | 2 | 61947475 | 45,47 | 36,29 | 9127708 |  |  |
| Vua428b | *D. alata* | Vanuatu | Pacific | 2 | 62448757 | 45,02 | 36,91 | 8753693 |  |  |

**Supplementary Table 2** : All genes found for the three selected pathways used in this study. When found by keyword analysis, the EC number present in the annotation file, the Orthology group which they belong, the method used to retrieve the gene, the predicted function annotation, and their KEGG pathway

| **Gene** | **EC Number** | **Genome** | **Retrieved from** | **Blast** | **KEGG**  **Pathway** |
| --- | --- | --- | --- | --- | --- |
|  |  |  |  | sp\|Q6JN47\|EIX1_SOLLC Receptor-like protein EIX1 OS=Solanum lycopersicum |  |
| Dioal.01G024300.1 |  | OG0000010 | Orthogroups | OX=4081 GN=EIX1 PE=2 SV=2 | map00040 |
|  |  |  |  | sp\|Q7FZR1\|RLP52_ARATH Receptor-like protein 52 OS=Arabidopsis thaliana |  |
| Dioal.01G024400.1 |  | OG0000010 | Orthogroups | OX=3702 GN=RLP52 PE=2 SV=1 | map00040 |
|  |  |  |  | sp\|Q6JN46\|EIX2_SOLLC Receptor-like protein EIX2 OS=Solanum lycopersicum |  |
| Dioal.01G024600.1 |  | OG0000010 | Orthogroups | OX=4081 GN=EIX2 PE=1 SV=2 | map00040 |
|  |  |  |  | sp\|Q9FRS6\|PXL1_ARATH Leucine-rich repeat receptor-like protein kinase PXL1 |  |
| Dioal.01G024700.1 |  | OG0000010 | Orthogroups | OS=Arabidopsis thaliana OX=3702 GN=PXL1 PE=1 SV=1 | map00040 |
|  |  |  |  | sp\|Q6JN46\|EIX2_SOLLC Receptor-like protein EIX2 OS=Solanum lycopersicum |  |
| Dioal.01G024800.1 |  | OG0000010 | Orthogroups | OX=4081 GN=EIX2 PE=1 SV=2 | map00040 |
|  |  |  |  | sp\|Q944R1\|PLY15_ARATH Probable pectate lyase 15 OS=Arabidopsis thaliana |  |
| Dioal.01G025700.1 | EC:4.2.2.2 | OG0000048 | Genome Annotation | OX=3702 GN=At4g13710 PE=2 SV=1 | map00040 |
|  |  |  |  | sp\|Q944R1\|PLY15_ARATH Probable pectate lyase 15 OS=Arabidopsis thaliana |  |
| Dioal.01G025800.1 | EC:4.2.2.2 | OG0000048 | Genome Annotation | OX=3702 GN=At4g13710 PE=2 SV=1 | map00040 |
|  |  |  |  | sp\|Q9ZQA3\|PME15_ARATH Probable pectinesterase 15 OS=Arabidopsis thaliana |  |
| Dioal.01G032100.1 | EC:3.1.1.11 | OG0003143 | Genome Annotation | OX=3702 GN=PME15 PE=2 SV=1 | map00040 |
|  |  |  |  | sp\|Q944R1\|PLY15_ARATH Probable pectate lyase 15 OS=Arabidopsis thaliana |  |
| Dioal.01G035100.1 | EC:4.2.2.2 | OG0000048 | Genome Annotation | OX=3702 GN=At4g13710 PE=2 SV=1 | map00040 |
|  |  |  |  | sp\|Q6JN46\|EIX2_SOLLC Receptor-like protein EIX2 OS=Solanum lycopersicum |  |
| Dioal.01G036500.1 |  | OG0000010 | Orthogroups | OX=4081 GN=EIX2 PE=1 SV=2 | map00040 |
|  |  |  |  | sp\|Q6JN46\|EIX2_SOLLC Receptor-like protein EIX2 OS=Solanum lycopersicum |  |
| Dioal.01G036800.1 |  | OG0000010 | Orthogroups | OX=4081 GN=EIX2 PE=1 SV=2 | map00040 |
|  |  |  |  | sp\|Q9SI72\|PMEI9_ARATH Pectinesterase inhibitor 9 OS=Arabidopsis thaliana |  |
| Dioal.01G079100.1 | EC:3.1.1.11 | OG0000198 | Genome Annotation | OX=3702 GN=PMEI9 PE=2 SV=1 | map00040 |
|  |  |  |  | sp\|Q6JN46\|EIX2_SOLLC Receptor-like protein EIX2 OS=Solanum lycopersicum |  |
| Dioal.01G079500.1 | EC:3.2.1.15 | OG0000010 | Genome Annotation | OX=4081 GN=EIX2 PE=1 SV=2 | map00040 |
|  |  |  |  | sp\|Q84R10\|PME36_ARATH Probable pectinesterase/pectinesterase inhibitor 36 |  |
| Dioal.01G084900.1 | EC:3.1.1.11 | OG0000009 | Genome Annotation | OS=Arabidopsis thaliana OX=3702 GN=PME36 PE=2 SV=2 | map00040 |

|  |  |  |  | sp\|Q8GT41\|PLA1_PLAAC Putative invertase inhibitor OS=Platanus acerifolia |  |
| --- | --- | --- | --- | --- | --- |
| Dioal.01G090800.1 |  | OG0000666 | Orthogroups | OX=140101 PE=1 SV=1 | map00040 |
|  |  |  |  | sp\|Q6JN46\|EIX2_SOLLC Receptor-like protein EIX2 OS=Solanum lycopersicum |  |
| Dioal.02G029400.1 |  | OG0000010 | Orthogroups | OX=4081 GN=EIX2 PE=1 SV=2 | map00040 |
|  |  |  |  | sp\|Q6JN46\|EIX2_SOLLC Receptor-like protein EIX2 OS=Solanum lycopersicum |  |
| Dioal.02G041200.1 |  | OG0000010 | Orthogroups | OX=4081 GN=EIX2 PE=1 SV=2 | map00040 |
|  |  |  |  | sp\|Q9SCP2\|PLY12_ARATH Probable pectate lyase 12 OS=Arabidopsis thaliana |  |
| Dioal.02G059800.1 | EC:4.2.2.2 | OG0000048 | Genome Annotation | OX=3702 GN=At3g53190 PE=2 SV=2 | map00040 |
|  |  |  |  | sp\|Q6JN47\|EIX1_SOLLC Receptor-like protein EIX1 OS=Solanum lycopersicum |  |
| Dioal.02G061100.1 |  | OG0000010 | Orthogroups | OX=4081 GN=EIX1 PE=2 SV=2 | map00040 |
|  |  |  |  | sp\|Q6JN46\|EIX2_SOLLC Receptor-like protein EIX2 OS=Solanum lycopersicum |  |
| Dioal.02G061200.1 |  | OG0000010 | Orthogroups | OX=4081 GN=EIX2 PE=1 SV=2 | map00040 |
|  |  |  |  | sp\|Q6JN46\|EIX2_SOLLC Receptor-like protein EIX2 OS=Solanum lycopersicum |  |
| Dioal.02G061300.1 |  | OG0000010 | Orthogroups | OX=4081 GN=EIX2 PE=1 SV=2 | map00040 |
|  |  |  |  | sp\|Q6JN46\|EIX2_SOLLC Receptor-like protein EIX2 OS=Solanum lycopersicum |  |
| Dioal.02G061400.1 |  | OG0000010 | Orthogroups | OX=4081 GN=EIX2 PE=1 SV=2 | map00040 |
|  |  |  |  | sp\|Q6JN47\|EIX1_SOLLC Receptor-like protein EIX1 OS=Solanum lycopersicum |  |
| Dioal.02G061500.1 |  | OG0000010 | Orthogroups | OX=4081 GN=EIX1 PE=2 SV=2 | map00040 |
|  |  |  |  | sp\|Q6JN47\|EIX1_SOLLC Receptor-like protein EIX1 OS=Solanum lycopersicum |  |
| Dioal.02G061600.1 |  | OG0000010 | Orthogroups | OX=4081 GN=EIX1 PE=2 SV=2 | map00040 |
|  |  |  |  | sp\|Q6JN46\|EIX2_SOLLC Receptor-like protein EIX2 OS=Solanum lycopersicum |  |
| Dioal.02G061800.1 |  | OG0000010 | Orthogroups | OX=4081 GN=EIX2 PE=1 SV=2 | map00040 |
|  |  |  |  | sp\|Q6JN46\|EIX2_SOLLC Receptor-like protein EIX2 OS=Solanum lycopersicum |  |
| Dioal.02G061900.1 |  | OG0000010 | Orthogroups | OX=4081 GN=EIX2 PE=1 SV=2 | map00040 |
|  |  |  |  | sp\|Q6JN46\|EIX2_SOLLC Receptor-like protein EIX2 OS=Solanum lycopersicum |  |
| Dioal.02G062000.1 |  | OG0000010 | Orthogroups | OX=4081 GN=EIX2 PE=1 SV=2 | map00040 |
|  |  |  |  | sp\|Q6JN46\|EIX2_SOLLC Receptor-like protein EIX2 OS=Solanum lycopersicum |  |
| Dioal.02G062100.1 |  | OG0000010 | Orthogroups | OX=4081 GN=EIX2 PE=1 SV=2 | map00040 |
|  |  |  |  | sp\|Q6JN46\|EIX2_SOLLC Receptor-like protein EIX2 OS=Solanum lycopersicum |  |
| Dioal.02G062200.1 |  | OG0000010 | Orthogroups | OX=4081 GN=EIX2 PE=1 SV=2 | map00040 |
|  |  |  |  | sp\|Q6JN46\|EIX2_SOLLC Receptor-like protein EIX2 OS=Solanum lycopersicum |  |
| Dioal.02G062400.1 |  | OG0000010 | Orthogroups | OX=4081 GN=EIX2 PE=1 SV=2 | map00040 |
|  |  |  |  | sp\|Q6JN46\|EIX2_SOLLC Receptor-like protein EIX2 OS=Solanum lycopersicum |  |
| Dioal.02G062500.1 | EC:3.2.1.15 | OG0000010 | Genome Annotation | OX=4081 GN=EIX2 PE=1 SV=2 | map00040 |

|  | | | sp\|Q6JN46\|EIX2_SOLLC Receptor-like protein EIX2 OS=Solanum lycopersicum |  |
| --- | --- | --- | --- | --- |
| Dioal.02G062700.1 | OG0000010 | Orthogroups | OX=4081 GN=EIX2 PE=1 SV=2 | map00040 |
|  |  |  | sp\|Q6JN46\|EIX2_SOLLC Receptor-like protein EIX2 OS=Solanum lycopersicum |  |
| Dioal.02G062800.1 | OG0000010 | Orthogroups | OX=4081 GN=EIX2 PE=1 SV=2 | map00040 |
|  |  |  | sp\|Q6JN46\|EIX2_SOLLC Receptor-like protein EIX2 OS=Solanum lycopersicum |  |
| Dioal.02G064200.1 | OG0000010 | Orthogroups | OX=4081 GN=EIX2 PE=1 SV=2 | map00040 |
|  |  |  | sp\|Q6JN46\|EIX2_SOLLC Receptor-like protein EIX2 OS=Solanum lycopersicum |  |
| Dioal.02G064300.1 | OG0000010 | Orthogroups | OX=4081 GN=EIX2 PE=1 SV=3 | map00040 |
|  |  |  | sp\|Q6JN46\|EIX2_SOLLC Receptor-like protein EIX2 OS=Solanum lycopersicum |  |
| Dioal.02G064400.1 | OG0000010 | Orthogroups | OX=4081 GN=EIX2 PE=1 SV=2 | map00040 |
|  |  |  | sp\|Q6JN46\|EIX2_SOLLC Receptor-like protein EIX2 OS=Solanum lycopersicum |  |
| Dioal.02G064600.1 | OG0000010 | Orthogroups | OX=4081 GN=EIX2 PE=1 SV=2 | map00040 |
|  |  |  | sp\|Q6JN46\|EIX2_SOLLC Receptor-like protein EIX2 OS=Solanum lycopersicum |  |
| Dioal.02G064800.1 | OG0000010 | Orthogroups | OX=4081 GN=EIX2 PE=1 SV=2 | map00040 |
|  |  |  | sp\|Q6JN46\|EIX2_SOLLC Receptor-like protein EIX2 OS=Solanum lycopersicum |  |
| Dioal.02G064900.1 | OG0000010 | Orthogroups | OX=4081 GN=EIX2 PE=1 SV=2 | map00040 |
|  |  |  | sp\|Q6JN46\|EIX2_SOLLC Receptor-like protein EIX2 OS=Solanum lycopersicum |  |
| Dioal.02G065000.1 | OG0000010 | Orthogroups | OX=4081 GN=EIX2 PE=1 SV=2 | map00040 |
|  |  |  | sp\|Q6JN46\|EIX2_SOLLC Receptor-like protein EIX2 OS=Solanum lycopersicum |  |
| Dioal.02G065100.1 | OG0000010 | Orthogroups | OX=4081 GN=EIX2 PE=1 SV=2 | map00040 |
|  |  |  | sp\|Q6JN46\|EIX2_SOLLC Receptor-like protein EIX2 OS=Solanum lycopersicum |  |
| Dioal.02G065200.1 | OG0000010 | Orthogroups | OX=4081 GN=EIX2 PE=1 SV=2 | map00040 |
|  |  |  | sp\|Q6JN46\|EIX2_SOLLC Receptor-like protein EIX2 OS=Solanum lycopersicum |  |
| Dioal.02G065300.1 | OG0000010 | Orthogroups | OX=4081 GN=EIX2 PE=1 SV=2 | map00040 |
|  |  |  | sp\|Q6JN46\|EIX2_SOLLC Receptor-like protein EIX2 OS=Solanum lycopersicum |  |
| Dioal.02G066000.1 | OG0000010 | Orthogroups | OX=4081 GN=EIX2 PE=1 SV=2 | map00040 |
|  |  |  | sp\|Q6JN46\|EIX2_SOLLC Receptor-like protein EIX2 OS=Solanum lycopersicum |  |
| Dioal.02G066200.1 | OG0000010 | Orthogroups | OX=4081 GN=EIX2 PE=1 SV=2 | map00040 |
|  |  |  | sp\|Q6JN46\|EIX2_SOLLC Receptor-like protein EIX2 OS=Solanum lycopersicum |  |
| Dioal.02G066400.1 | OG0000010 | Orthogroups | OX=4081 GN=EIX2 PE=1 SV=2 | map00040 |
|  |  |  | sp\|Q6JN46\|EIX2_SOLLC Receptor-like protein EIX2 OS=Solanum lycopersicum |  |
| Dioal.02G066500.1 | OG0000010 | Orthogroups | OX=4081 GN=EIX2 PE=1 SV=2 | map00040 |
|  |  |  | sp\|Q6JN46\|EIX2_SOLLC Receptor-like protein EIX2 OS=Solanum lycopersicum |  |
| Dioal.02G066700.1 | OG0000010 | Orthogroups | OX=4081 GN=EIX2 PE=1 SV=2 | map00040 |

| Dioal.02G066900.1 Dioal.02G067100.1 Dioal.02G067200.1 Dioal.02G067400.1 Dioal.02G071200.1 Dioal.02G071300.1 |  | OG0000010 OG0000010 OG0000010 OG0000010 OG0000010 OG0000010 | Orthogroups Orthogroups Orthogroups Orthogroups Orthogroups Orthogroups | sp\|Q6JN46\|EIX2_SOLLC Receptor-like protein EIX2 OS=Solanum lycopersicum OX=4081 GN=EIX2 PE=1 SV=2  sp\|Q6JN46\|EIX2_SOLLC Receptor-like protein EIX2 OS=Solanum lycopersicum OX=4081 GN=EIX2 PE=1 SV=2  sp\|Q6JN46\|EIX2_SOLLC Receptor-like protein EIX2 OS=Solanum lycopersicum OX=4081 GN=EIX2 PE=1 SV=2  sp\|Q6JN46\|EIX2_SOLLC Receptor-like protein EIX2 OS=Solanum lycopersicum OX=4081 GN=EIX2 PE=1 SV=2  sp\|Q6JN46\|EIX2_SOLLC Receptor-like protein EIX2 OS=Solanum lycopersicum OX=4081 GN=EIX2 PE=1 SV=2  sp\|Q6JN46\|EIX2_SOLLC Receptor-like protein EIX2 OS=Solanum lycopersicum OX=4081 GN=EIX2 PE=1 SV=2 | map00040 map00040 map00040 map00040 map00040 map00040 |
| --- | --- | --- | --- | --- | --- |
| Dioal.02G074500.1 Dioal.03G015800.1 | EC:5.3.1.5 | OG0000010 OG0007701 | Orthogroups Genome Annotation | sp\|Q9LN69\|PPR50_ARATH Putative pentatricopeptide repeat-containing protein At1g19290 OS=Arabidopsis thaliana OX=3702 GN=At1g19290 PE=3 SV=2 sp\|Q40082\|XYLA_HORVU Xylose isomerase OS=Hordeum vulgare OX=4513 GN=XYLA PE=1 SV=1 | map00040 map00040 |
| Dioal.03G035700.1 | EC:3.2.1.167 EC | OG0003419 | Genome Annotation | sp\|Q9FZP1\|HPSE3_ARATH Heparanase-like protein 3 OS=Arabidopsis thaliana  OX=3702 GN=At5g34940 PE=2 SV=2 | map00040 |
| Dioal.03G049500.1 | EC:5.3.1.5 | OG0082968 | Genome Annotation | sp\|Q9FKK7\|XYLA_ARATH Xylose isomerase OS=Arabidopsis thaliana OX=3702  GN=XYLA PE=2 SV=2 | map00040 |
| Dioal.03G088200.1 | EC:3.2.1.15 | OG0002643 | Genome Annotation | sp\|Q00874\|DR100_ARATH DNA damage-repair/toleration protein DRT100  OS=Arabidopsis thaliana OX=3702 GN=DRT100 PE=2 SV=2 | map00040 |
| Dioal.04G018700.1 | EC:3.2.1.15 | OG0000322 | Genome Annotation | sp\|P48979\|PGLR_PRUPE Polygalacturonase OS=Prunus persica OX=3760 PE=2 SV=1 | map00040 |
| Dioal.04G027900.1 | EC:2.4.1.17 | OG0000490 | Genome Annotation | sp\|Q9LSB1\|GUX1_ARATH UDP-glucuronate:xylan alpha-glucuronosyltransferase 1 OS=Arabidopsis thaliana OX=3702 GN=GUX1 PE=2 SV=1 | map00040 |
| Dioal.04G096300.1 Dioal.04G101400.1 | EC:3.1.1.11 | OG0000021 OG0001942 | Orthogroups Genome Annotation | sp\|C0LGE0\|Y1765_ARATH Probable LRR receptor-like serine/threonine-protein kinase At1g07650 OS=Arabidopsis thaliana OX=3702 GN=At1g07650 PE=1 SV=1 sp\|Q8VYZ3\|PME53_ARATH Probable pectinesterase 53 OS=Arabidopsis thaliana OX=3702 GN=PME53 PE=2 SV=1 | map00040 map00040 |

| Dioal.04G130000.1 | EC:3.1.1.11 | OG0000009 | Genome Annotation | sp\|O04887\|PME2_CITSI Pectinesterase 2 OS=Citrus sinensis OX=2711 GN=PECS-  2.1 PE=2 SV=1 | map00040 |
| --- | --- | --- | --- | --- | --- |
| Dioal.04G130600.1 | EC:3.1.1.11 | OG0000198 | Genome Annotation | sp\|P17407\|21KD_DAUCA 21 kDa protein OS=Daucus carota OX=4039 PE=2 SV=1 | map00040 |
| Dioal.04G130700.1 | EC:3.1.1.11 | OG0000198 | Genome Annotation | sp\|Q9SB37\|PMEI7_ARATH Pectinesterase inhibitor 7 OS=Arabidopsis thaliana OX=3702 GN=PMEI7 PE=2 SV=1 | map00040 |
| Dioal.04G130800.1 | EC:3.1.1.11 | OG0000198 | Genome Annotation | sp\|P17407\|21KD_DAUCA 21 kDa protein OS=Daucus carota OX=4039 PE=2 SV=1 | map00040 |
| Dioal.04G130900.1 | EC:3.1.1.11 | OG0000198 | Genome Annotation | sp\|P17407\|21KD_DAUCA 21 kDa protein OS=Daucus carota OX=4039 PE=2 SV=1 | map00040 |
| Dioal.04G131000.1 | EC:3.1.1.11 | OG0000198 | Genome Annotation | sp\|P17407\|21KD_DAUCA 21 kDa protein OS=Daucus carota OX=4039 PE=2 SV=1 | map00040 |
| Dioal.04G141500.1 | EC:3.1.1.11 | OG0000666 | Genome Annotation | sp\|A9YUH4\|PLAO1_PLAOI Putative invertase inhibitor OS=Platanus orientalis  OX=122832 PE=1 SV=1 | map00040 |
| Dioal.04G180800.1 | EC:3.1.1.11 | OG0009313 | Genome Annotation | sp\|Q8LPF3\|PME68_ARATH Probable pectinesterase 68 OS=Arabidopsis thaliana OX=3702 GN=PME68 PE=2 SV=1 | map00040 |
| Dioal.05G026200.1 | EC:2.4.1.17 | OG0000490 | Genome Annotation | sp\|Q9LSB1\|GUX1_ARATH UDP-glucuronate:xylan alpha-glucuronosyltransferase 1 OS=Arabidopsis thaliana OX=3702 GN=GUX1 PE=2 SV=1 | map00040 |
| Dioal.05G026300.1 | EC:3.1.1.11 | OG0002470 | Genome Annotation | sp\|Q9LXD9\|PME51_ARATH Probable pectinesterase/pectinesterase inhibitor 51  OS=Arabidopsis thaliana OX=3702 GN=PME51 PE=2 SV=1 | map00040 |
| Dioal.05G044700.1 | EC:3.2.1.15 | OG0000322 | Genome Annotation | sp\|P48979\|PGLR_PRUPE Polygalacturonase OS=Prunus persica OX=3760 PE=2  SV=1 | map00040 |
| Dioal.05G046500.1 | EC:3.1.1.11 | OG0018898 | Genome Annotation | sp\|Q43867\|PME1_ARATH Pectinesterase 1 OS=Arabidopsis thaliana OX=3702  GN=PME1 PE=1 SV=1 | map00040 |
| Dioal.05G064400.1 | EC:4.2.2.2 | OG0000048 | Genome Annotation | sp\|Q93Z25\|PLY22_ARATH Probable pectate lyase 22 OS=Arabidopsis thaliana  OX=3702 GN=At5g63180 PE=2 SV=1 | map00040 |
| Dioal.05G072900.1 |  | OG0030054 | Orthogroups | sp\|Q766Z3\|REV3_ARATH DNA polymerase zeta catalytic subunit OS=Arabidopsis thaliana OX=3702 GN=REV3 PE=2 SV=1 | map00040 |
| Dioal.05G087200.1 | EC:4.2.2.2 | OG0000048 | Genome Annotation | sp\|P40973\|PLY_LILLO Pectate lyase OS=Lilium longiflorum OX=4690 PE=2 SV=1 | map00040 |
| Dioal.05G105900.1 | EC:4.2.2.2 | OG0000048 | Genome Annotation | sp\|O65388\|PLY2_ARATH Putative pectate lyase 2 OS=Arabidopsis thaliana  OX=3702 GN=At1g11920 PE=3 SV=2 | map00040 |

| Dioal.05G141700.1 | EC:3.2.1.15 | OG0002811 | Genome Annotation | sp\|Q949Z1\|PGLR4_ARATH Polygalacturonase At1g48100 OS=Arabidopsis  thaliana OX=3702 GN=At1g48100 PE=2 SV=1 | map00040 |
| --- | --- | --- | --- | --- | --- |
| Dioal.05G183500.1 Dioal.05G185100.1 | EC:3.1.1.11 | OG0000010 OG0000198 | Orthogroups Genome Annotation | sp\|Q6JN46\|EIX2_SOLLC Receptor-like protein EIX2 OS=Solanum lycopersicum OX=4081 GN=EIX2 PE=1 SV=2  sp\|Q9SB37\|PMEI7_ARATH Pectinesterase inhibitor 7 OS=Arabidopsis thaliana  OX=3702 GN=PMEI7 PE=2 SV=1 | map00040 map00040 |
| Dioal.05G191700.1 | EC:1.1.1.179 | OG0001528 | Genome Annotation | sp\|Q9SZ83\|Y4967_ARATH Uncharacterized oxidoreductase At4g09670  OS=Arabidopsis thaliana OX=3702 GN=At4g09670 PE=1 SV=1 | map00040 |
| Dioal.05G197800.1 | EC:3.1.1.11 | OG0000666 | Genome Annotation | sp\|A9YUH4\|PLAO1_PLAOI Putative invertase inhibitor OS=Platanus orientalis  OX=122832 PE=1 SV=1 | map00040 |
| Dioal.05G197900.1 | EC:3.1.1.11 | OG0000666 | Genome Annotation | sp\|A9YUH4\|PLAO1_PLAOI Putative invertase inhibitor OS=Platanus orientalis OX=122832 PE=1 SV=1 | map00040 |
| Dioal.05G198000.1 | EC:3.1.1.11 | OG0000009 | Genome Annotation | sp\|P83948\|PME3_CITSI Pectinesterase 3 OS=Citrus sinensis OX=2711 PE=1 SV=1 | map00040 |
| Dioal.05G213900.1 | EC:3.1.1.11 | OG0000009 | Genome Annotation | sp\|Q9FJ21\|PME58_ARATH Probable pectinesterase/pectinesterase inhibitor 58  OS=Arabidopsis thaliana OX=3702 GN=PME58 PE=2 SV=1 | map00040 |
| Dioal.05G232900.1 | EC:3.2.1.15 | OG0000086 | Genome Annotation | sp\|Q6H9K0\|PGLR2_PLAAC Exopolygalacturonase (Fragment) OS=Platanus  acerifolia OX=140101 GN=plaa2 PE=1 SV=1 | map00040 |
| Dioal.05G234400.1 |  | OG0007130 | Orthogroups | A0A6I9QRX2_ELAGV\|uncharacterized protein LOC105039462 isoform X1 | map00040 |
| Dioal.05G234500.1 | EC:1.1.1.179 EC | OG0007130 | Genome Annotation | A0A6I9QRX2_ELAGV\|uncharacterized protein LOC105039462 isoform X2 | map00040 |
| Dioal.05G234600.1  Dioal.05G236800.1 | EC:3.2.1.15 | OG0007130  OG0000086 | Orthogroups  Genome Annotation | A0A6I9QRX2_ELAGV\|uncharacterized protein LOC105039462 isoform X3 sp\|Q6H9K0\|PGLR2_PLAAC Exopolygalacturonase (Fragment) OS=Platanus  acerifolia OX=140101 GN=plaa2 PE=1 SV=1 | map00040  map00040 |
| Dioal.06G005800.1 | EC:3.2.1.15 | OG0001446 | Genome Annotation | sp\|Q949Z1\|PGLR4_ARATH Polygalacturonase At1g48100 OS=Arabidopsis  thaliana OX=3702 GN=At1g48100 PE=2 SV=1 | map00040 |
| Dioal.06G011700.1 | EC:3.1.1.11 | OG0000009 | Genome Annotation | sp\|Q3E8Z8\|PME28_ARATH Putative pectinesterase/pectinesterase inhibitor 28  OS=Arabidopsis thaliana OX=3702 GN=PME28 PE=2 SV=1 | map00040 |
| Dioal.06G013000.1 | EC:3.1.1.11 | OG0000009 | Genome Annotation | sp\|O22256\|PME20_ARATH Probable pectinesterase/pectinesterase inhibitor 20  OS=Arabidopsis thaliana OX=3702 GN=PME20 PE=2 SV=2 | map00040 |
| Dioal.06G024200.1 Dioal.06G033200.1 |  | OG0000021 OG0005426 | Orthogroups Orthogroups | sp\|Q93YN1\|CRPK1_ARATH Cold-responsive protein kinase 1 OS=Arabidopsis thaliana OX=3702 GN=CRPK1 PE=1 SV=1  sp\|Q940S3\|UAP1_ARATH UDP-N-acetylglucosamine diphosphorylase 1  OS=Arabidopsis thaliana OX=3702 GN=GLCNAC1PUT1 PE=1 SV=1 | map00040 map00040 |

Dioal.06G057500.1 EC:2.4.1.17 OG0000490 Genome Annotation Dioal.06G072100.1 EC:4.2.2.2 OG0000048 Genome Annotation Dioal.06G072400.1 EC:4.2.2.2 OG0000048 Genome Annotation

sp|Q8GWW4|GUX2_ARATH UDP-glucuronate:xylan alpha- glucuronosyltransferase 2 OS=Arabidopsis thaliana OX=3702 GN=GUX2 PE=2

SV=1 map00040

sp|Q9C8G4|PLY4_ARATH Probable pectate lyase 4 OS=Arabidopsis thaliana

OX=3702 GN=At1g30350 PE=2 SV=1 map00040

sp|P18632|PLY1_CRYJA Pectate lyase 1 OS=Cryptomeria japonica OX=3369 PE=1

SV=3 map00040

Dioal.06G093700.1 EC:3.1.1.11 OG0046047 Genome Annotation A0A8B7MTC5_PHODC|pectinesterase inhibitor 3-like map00040

sp|Q84WM7|PPME1_ARATH Pectinesterase PPME1 OS=Arabidopsis thaliana

Dioal.06G094500.1 EC:3.1.1.11 OG0001549 Genome Annotation Dioal.06G096600.1 EC:3.1.1.11 OG0000009 Genome Annotation Dioal.06G096700.1 EC:3.1.1.11 OG0000009 Genome Annotation Dioal.06G096800.1 EC:3.1.1.11 OG0000009 Genome Annotation Dioal.07G008700.1 EC:3.2.1.15 OG0001446 Genome Annotation

Dioal.07G012800.1 EC:3.1.1.11 OG0009398 Genome Annotation

OX=3702 GN=PPME1 PE=1 SV=1 map00040

sp|Q7Y201|PME13_ARATH Probable pectinesterase/pectinesterase inhibitor 13 OS=Arabidopsis thaliana OX=3702 GN=PME13 PE=2 SV=2 map00040 sp|Q94CB1|PME25_ARATH Probable pectinesterase/pectinesterase inhibitor 25 OS=Arabidopsis thaliana OX=3702 GN=PME25 PE=2 SV=1 map00040 sp|Q43062|PME_PRUPE Pectinesterase/pectinesterase inhibitor PPE8B

OS=Prunus persica OX=3760 PE=2 SV=1 map00040 sp|Q949Z1|PGLR4_ARATH Polygalacturonase At1g48100 OS=Arabidopsis

thaliana OX=3702 GN=At1g48100 PE=2 SV=1 map00040 sp|Q9LSP1|PME67_ARATH Probable pectinesterase 67 OS=Arabidopsis thaliana

OX=3702 GN=PME67 PE=2 SV=1 map00040

Dioal.07G015800.1 EC:3.1.1.11 OG0026537 Genome Annotation A0A8B7MTC5_PHODC|pectinesterase inhibitor 3-like map00040

sp|Q3E8Z8|PME28_ARATH Putative pectinesterase/pectinesterase inhibitor 28

Dioal.07G018800.1 EC:3.1.1.11 OG0000009 Genome Annotation Dioal.07G019000.1 EC:3.1.1.11 OG0000009 Genome Annotation Dioal.07G026400.1 EC:3.1.1.11 OG0003890 Genome Annotation Dioal.07G026500.1 EC:3.1.1.11 EC: OG0000009 Genome Annotation Dioal.07G026600.1 EC:3.1.1.11 OG0000009 Genome Annotation Dioal.07G027000.1 EC:3.2.1.15 OG0000086 Genome Annotation

OS=Arabidopsis thaliana OX=3702 GN=PME28 PE=2 SV=1 map00040 sp|Q3E8Z8|PME28_ARATH Putative pectinesterase/pectinesterase inhibitor 28 OS=Arabidopsis thaliana OX=3702 GN=PME28 PE=2 SV=1 map00040 sp|A9YUH4|PLAO1_PLAOI Putative invertase inhibitor OS=Platanus orientalis

OX=122832 PE=1 SV=1 map00040

sp|Q8RXK7|PME41_ARATH Probable pectinesterase/pectinesterase inhibitor 41 OS=Arabidopsis thaliana OX=3702 GN=PME41 PE=2 SV=2 map00040 sp|Q9M9W7|PME22_ARATH Putative pectinesterase/pectinesterase inhibitor 22 OS=Arabidopsis thaliana OX=3702 GN=PME22 PE=3 SV=1 map00040 sp|Q05967|PGLR_TOBAC Polygalacturonase OS=Nicotiana tabacum OX=4097

GN=PG1 PE=2 SV=1 map00040

Dioal.07G037500.1 EC:5.3.1.5 OG0007701 Genome Annotation Dioal.07G045100.1 EC:3.1.1.11 OG0002470 Genome Annotation Dioal.07G068800.1 OG0000010 Orthogroups

Dioal.07G101200.1 OG0000010 Orthogroups Dioal.08G021600.1 EC:2.7.1.17 OG0006496 Genome Annotation Dioal.08G045800.1 EC:3.2.1.15 OG0000086 Genome Annotation Dioal.08G050900.1 EC:3.2.1.15 OG0002643 Genome Annotation Dioal.08G080700.1 OG0000010 Orthogroups

Dioal.08G080800.1 OG0000010 Orthogroups

Dioal.08G081100.1 OG0000010 Orthogroups

Dioal.08G081200.1 OG0000010 Orthogroups Dioal.08G089400.1 EC:2.7.7.10 EC: OG0007363 Genome Annotation Dioal.08G105700.1 EC:3.1.1.11 OG0016372 Genome Annotation Dioal.08G114300.1 EC:3.2.1.167 EC OG0003419 Genome Annotation

sp|Q40082|XYLA_HORVU Xylose isomerase OS=Hordeum vulgare OX=4513

GN=XYLA PE=1 SV=1 map00040

sp|Q9LXD9|PME51_ARATH Probable pectinesterase/pectinesterase inhibitor 51 OS=Arabidopsis thaliana OX=3702 GN=PME51 PE=2 SV=1 map00040 sp|Q6JN46|EIX2_SOLLC Receptor-like protein EIX2 OS=Solanum lycopersicum

OX=4081 GN=EIX2 PE=1 SV=2 map00040

sp|Q6JN46|EIX2_SOLLC Receptor-like protein EIX2 OS=Solanum lycopersicum

OX=4081 GN=EIX2 PE=1 SV=2 map00040

sp|Q949W8|XK2_ARATH Xylulose kinase 2 OS=Arabidopsis thaliana OX=3702

GN=XK2 PE=1 SV=1 map00040

sp|Q6H9K0|PGLR2_PLAAC Exopolygalacturonase (Fragment) OS=Platanus

acerifolia OX=140101 GN=plaa2 PE=1 SV=1 map00040 sp|Q00874|DR100_ARATH DNA damage-repair/toleration protein DRT100

OS=Arabidopsis thaliana OX=3702 GN=DRT100 PE=2 SV=2 map00040 sp|Q6JN46|EIX2_SOLLC Receptor-like protein EIX2 OS=Solanum lycopersicum

OX=4081 GN=EIX2 PE=1 SV=2 map00040

sp|Q6JN46|EIX2_SOLLC Receptor-like protein EIX2 OS=Solanum lycopersicum

OX=4081 GN=EIX2 PE=1 SV=2 map00040

sp|Q6JN46|EIX2_SOLLC Receptor-like protein EIX2 OS=Solanum lycopersicum

OX=4081 GN=EIX2 PE=1 SV=2 map00040

sp|Q6JN46|EIX2_SOLLC Receptor-like protein EIX2 OS=Solanum lycopersicum

OX=4081 GN=EIX2 PE=1 SV=2 map00040

sp|A2YGP6|USP_ORYSI UDP-sugar pyrophosphorylase OS=Oryza sativa subsp.

indica OX=39946 GN=USP PE=3 SV=2 map00040

sp|P83326|PMEI_ACTDE Pectinesterase inhibitor OS=Actinidia deliciosa OX=3627

GN=PMEI PE=1 SV=2 map00040

sp|Q9FZP1|HPSE3_ARATH Heparanase-like protein 3 OS=Arabidopsis thaliana

OX=3702 GN=At5g34940 PE=2 SV=2 map00040

Dioal.09G009200.1 EC:2.7.1.16 OG0008763 Genome Annotation A0A804HNX8_MUSAM|FGGY_C domain-containing protein map00040

Dioal.09G066900.1 EC:4.2.2.2 OG0018490 Genome Annotation sp|P40973|PLY_LILLO Pectate lyase OS=Lilium longiflorum OX=4690 PE=2 SV=1 map00040

sp|Q93ZC9|GLAK1_ARATH Glucuronokinase 1 OS=Arabidopsis thaliana OX=3702

Dioal.09G071100.1 EC:2.7.1.43 OG0009558 Genome Annotation GN=GLCAK1 PE=1 SV=1 map00040

| Dioal.09G071800.1 | EC:4.2.2.2 | OG0003594 | Genome Annotation | sp\|Q9FM66\|PLY21_ARATH Putative pectate lyase 21 OS=Arabidopsis thaliana OX=3702 GN=At5g55720 PE=3 SV=1 | map00040 |
| --- | --- | --- | --- | --- | --- |
| Dioal.10G020100.1 | EC:5.1.3.1 | OG0008340 | Genome Annotation | sp\|Q9SE42\|RPE1_ORYSJ Ribulose-phosphate 3-epimerase, cytoplasmic isoform OS=Oryza sativa subsp. japonica OX=39947 GN=Os09g0505700 PE=1 SV=1 | map00040 |
| Dioal.10G043000.1 | EC:4.2.2.2 | OG0000048 | Genome Annotation | sp\|Q93Z25\|PLY22_ARATH Probable pectate lyase 22 OS=Arabidopsis thaliana  OX=3702 GN=At5g63180 PE=2 SV=1 | map00040 |
| Dioal.10G055200.1 | EC:3.2.1.15 | OG0000322 | Genome Annotation | sp\|P48979\|PGLR_PRUPE Polygalacturonase OS=Prunus persica OX=3760 PE=2  SV=1 | map00040 |
| Dioal.10G055300.1 | EC:3.2.1.15 | OG0000322 | Genome Annotation | sp\|P48979\|PGLR_PRUPE Polygalacturonase OS=Prunus persica OX=3760 PE=2  SV=1 | map00040 |
| Dioal.11G009600.1 | EC:3.1.1.11 | OG0000198 | Genome Annotation | sp\|Q9SI72\|PMEI9_ARATH Pectinesterase inhibitor 9 OS=Arabidopsis thaliana  OX=3702 GN=PMEI9 PE=2 SV=1 | map00040 |
| Dioal.11G019600.1 | EC:3.1.1.11 | OG0018898 | Genome Annotation | sp\|Q43867\|PME1_ARATH Pectinesterase 1 OS=Arabidopsis thaliana OX=3702  GN=PME1 PE=1 SV=1 | map00040 |
| Dioal.11G025100.1 | EC:3.1.1.11 | OG0000666 | Genome Annotation | sp\|Q8GT41\|PLA1_PLAAC Putative invertase inhibitor OS=Platanus acerifolia  OX=140101 PE=1 SV=1 | map00040 |
| Dioal.11G040500.1 | EC:1.1.1.14 | OG0004242 | Genome Annotation | sp\|Q9FJ95\|DHSO_ARATH Sorbitol dehydrogenase OS=Arabidopsis thaliana  OX=3702 GN=SDH PE=1 SV=1 | map00040 |
| Dioal.11G061100.1 | EC:1.1.1.14 | OG0000021 | Orthogroups | sp\|Q93YN1\|CRPK1_ARATH Cold-responsive protein kinase 1 OS=Arabidopsis  thaliana OX=3702 GN=CRPK1 PE=1 SV=1 | map00040 |
| Dioal.11G072500.1 | EC:2.7.7.9 | OG0001502 | Genome Annotation | sp\|Q9SDX3\|UGPA_MUSAC UTP--glucose-1-phosphate uridylyltransferase  OS=Musa acuminata OX=4641 GN=UGPA PE=2 SV=1 | map00040 |
| Dioal.11G077600.1 | EC:2.7.11.1 EC: | OG0000010 | Genome Annotation | sp\|Q6JN46\|EIX2_SOLLC Receptor-like protein EIX2 OS=Solanum lycopersicum  OX=4081 GN=EIX2 PE=1 SV=2 | map00040 |
| Dioal.11G077700.1 Dioal.11G077800.1 Dioal.11G077900.1 Dioal.11G078100.1 |  | OG0000010 OG0000010 OG0000010 OG0000010 | Orthogroups Orthogroups Orthogroups Orthogroups | sp\|Q6JN46\|EIX2_SOLLC Receptor-like protein EIX2 OS=Solanum lycopersicum OX=4081 GN=EIX2 PE=1 SV=2  sp\|Q6JN46\|EIX2_SOLLC Receptor-like protein EIX2 OS=Solanum lycopersicum OX=4081 GN=EIX2 PE=1 SV=2  sp\|Q6JN46\|EIX2_SOLLC Receptor-like protein EIX2 OS=Solanum lycopersicum OX=4081 GN=EIX2 PE=1 SV=2  sp\|Q6JN46\|EIX2_SOLLC Receptor-like protein EIX2 OS=Solanum lycopersicum  OX=4081 GN=EIX2 PE=1 SV=2 | map00040 map00040 map00040 map00040 |

|  | | | sp\|Q6JN46\|EIX2_SOLLC Receptor-like protein EIX2 OS=Solanum lycopersicum |  |
| --- | --- | --- | --- | --- |
| Dioal.11G078200.1 | OG0000010 | Orthogroups | OX=4081 GN=EIX2 PE=1 SV=2 | map00040 |
|  |  |  | sp\|Q6JN46\|EIX2_SOLLC Receptor-like protein EIX2 OS=Solanum lycopersicum |  |
| Dioal.11G078300.1 | OG0000010 | Orthogroups | OX=4081 GN=EIX2 PE=1 SV=2 | map00040 |
|  |  |  | sp\|Q6JN46\|EIX2_SOLLC Receptor-like protein EIX2 OS=Solanum lycopersicum |  |
| Dioal.11G078400.1 | OG0000010 | Orthogroups | OX=4081 GN=EIX2 PE=1 SV=2 | map00040 |
|  |  |  | sp\|Q6JN46\|EIX2_SOLLC Receptor-like protein EIX2 OS=Solanum lycopersicum |  |
| Dioal.11G078500.1 | OG0000010 | Orthogroups | OX=4081 GN=EIX2 PE=1 SV=2 | map00040 |
|  |  |  | sp\|Q6JN46\|EIX2_SOLLC Receptor-like protein EIX2 OS=Solanum lycopersicum |  |
| Dioal.11G078600.1 | OG0000010 | Orthogroups | OX=4081 GN=EIX2 PE=1 SV=2 | map00040 |
|  |  |  | sp\|Q6JN46\|EIX2_SOLLC Receptor-like protein EIX2 OS=Solanum lycopersicum |  |
| Dioal.11G078800.1 | OG0000010 | Orthogroups | OX=4081 GN=EIX2 PE=1 SV=2 | map00040 |
|  |  |  | sp\|Q6JN46\|EIX2_SOLLC Receptor-like protein EIX2 OS=Solanum lycopersicum |  |
| Dioal.11G079700.1 | OG0000010 | Orthogroups | OX=4081 GN=EIX2 PE=1 SV=2 | map00040 |
|  |  |  | sp\|Q6JN46\|EIX2_SOLLC Receptor-like protein EIX2 OS=Solanum lycopersicum |  |
| Dioal.11G079800.1 | OG0000010 | Orthogroups | OX=4081 GN=EIX2 PE=1 SV=2 | map00040 |
|  |  |  | sp\|Q6JN46\|EIX2_SOLLC Receptor-like protein EIX2 OS=Solanum lycopersicum |  |
| Dioal.11G080000.1 | OG0000010 | Orthogroups | OX=4081 GN=EIX2 PE=1 SV=2 | map00040 |
|  |  |  | sp\|Q6JN46\|EIX2_SOLLC Receptor-like protein EIX2 OS=Solanum lycopersicum |  |
| Dioal.11G080100.1 | OG0000010 | Orthogroups | OX=4081 GN=EIX2 PE=1 SV=2 | map00040 |
|  |  |  | sp\|Q6JN46\|EIX2_SOLLC Receptor-like protein EIX2 OS=Solanum lycopersicum |  |
| Dioal.11G080200.1 | OG0000010 | Orthogroups | OX=4081 GN=EIX2 PE=1 SV=2 | map00040 |
|  |  |  | sp\|Q6JN46\|EIX2_SOLLC Receptor-like protein EIX2 OS=Solanum lycopersicum |  |
| Dioal.11G080300.1 | OG0000010 | Orthogroups | OX=4081 GN=EIX2 PE=1 SV=2 | map00040 |
|  |  |  | sp\|Q6JN46\|EIX2_SOLLC Receptor-like protein EIX2 OS=Solanum lycopersicum |  |
| Dioal.11G080400.1 | OG0000010 | Orthogroups | OX=4081 GN=EIX2 PE=1 SV=2 | map00040 |
|  |  |  | sp\|Q6JN46\|EIX2_SOLLC Receptor-like protein EIX2 OS=Solanum lycopersicum |  |
| Dioal.11G080500.1 | OG0000010 | Orthogroups | OX=4081 GN=EIX2 PE=1 SV=2 | map00040 |
|  |  |  | sp\|Q6JN46\|EIX2_SOLLC Receptor-like protein EIX2 OS=Solanum lycopersicum |  |
| Dioal.11G081900.1 | OG0000010 | Orthogroups | OX=4081 GN=EIX2 PE=1 SV=2 | map00040 |
|  |  |  | sp\|Q6JN46\|EIX2_SOLLC Receptor-like protein EIX2 OS=Solanum lycopersicum |  |
| Dioal.11G082000.1 | OG0000010 | Orthogroups | OX=4081 GN=EIX2 PE=1 SV=2 | map00040 |
|  |  |  | sp\|Q6JN46\|EIX2_SOLLC Receptor-like protein EIX2 OS=Solanum lycopersicum |  |
| Dioal.11G082200.1 | OG0000010 | Orthogroups | OX=4081 GN=EIX2 PE=1 SV=2 | map00040 |

Dioal.11G082300.1 OG0000010 Orthogroups

Dioal.11G082400.1 OG0000010 Orthogroups Dioal.11G093000.1 EC:4.2.2.2 OG0000048 Genome Annotation

sp|Q6JN46|EIX2_SOLLC Receptor-like protein EIX2 OS=Solanum lycopersicum

OX=4081 GN=EIX2 PE=1 SV=2 map00040

sp|Q6JN46|EIX2_SOLLC Receptor-like protein EIX2 OS=Solanum lycopersicum

OX=4081 GN=EIX2 PE=1 SV=2 map00040

sp|Q944R1|PLY15_ARATH Probable pectate lyase 15 OS=Arabidopsis thaliana

OX=3702 GN=At4g13710 PE=2 SV=1 map00040

Dioal.11G103300.1 EC:2.4.1.17 OG0005187 Genome Annotation Dioal.12G004700.1 EC:3.2.1.15 OG0001446 Genome Annotation Dioal.12G006500.1 EC:3.1.1.11 OG0001067 Genome Annotation Dioal.12G012800.1 OG0003890 Orthogroups Dioal.12G014900.1 EC:3.1.1.11 OG0011954 Genome Annotation Dioal.12G015500.1 EC:5.1.3.1 OG0007379 Genome Annotation Dioal.12G025500.1 EC:3.2.1.15 OG0003721 Genome Annotation Dioal.12G079400.1 OG0000010 Orthogroups Dioal.12G079700.1 EC:4.2.2.2 OG0003594 Genome Annotation Dioal.13G014400.1 EC:3.1.1.11 OG0008212 Genome Annotation Dioal.13G021200.1 EC:3.2.1.167 EC OG0001270 Genome Annotation Dioal.13G044000.1 EC:3.2.1.15 OG0001060 Genome Annotation

sp|Q8GWB7|GUX6_ARATH Inositol phosphorylceramide glucuronosyltransferase

1 OS=Arabidopsis thaliana OX=3702 GN=IPUT1 PE=1 SV=1 map00040 sp|Q949Z1|PGLR4_ARATH Polygalacturonase At1g48100 OS=Arabidopsis

thaliana OX=3702 GN=At1g48100 PE=2 SV=1 map00040 sp|Q4PSN0|PME29_ARATH Probable pectinesterase 29 OS=Arabidopsis thaliana

OX=3702 GN=PME29 PE=2 SV=1 map00040

sp|O49603|CVIF2_ARATH Cell wall / vacuolar inhibitor of fructosidase 2

OS=Arabidopsis thaliana OX=3702 GN=C/VIF2 PE=1 SV=1 map00040 sp|O22244|PMEI6_ARATH Pectinesterase inhibitor 6 OS=Arabidopsis thaliana

OX=3702 GN=PMEI6 PE=2 SV=1 map00040

sp|Q43157|RPE_SPIOL Ribulose-phosphate 3-epimerase, chloroplastic

OS=Spinacia oleracea OX=3562 GN=RPE PE=1 SV=1 map00040 sp|G9LZD7|XIAO_ORYSJ Probable inactive leucine-rich repeat receptor kinase

XIAO OS=Oryza sativa subsp. japonica OX=39947 GN=XIAO PE=2 SV=1 map00040 sp|Q9LNV9|RLP1_ARATH Receptor-like protein 1 OS=Arabidopsis thaliana

OX=3702 GN=RLP1 PE=2 SV=2 map00040

sp|Q9C8G4|PLY4_ARATH Probable pectate lyase 4 OS=Arabidopsis thaliana

OX=3702 GN=At1g30350 PE=2 SV=1 map00040

sp|Q9FM79|PME62_ARATH Pectinesterase QRT1 OS=Arabidopsis thaliana

OX=3702 GN=QRT1 PE=2 SV=1 map00040

sp|Q8L608|HPSE2_ARATH Heparanase-like protein 2 OS=Arabidopsis thaliana

OX=3702 GN=At5g61250 PE=2 SV=1 map00040

sp|P35336|PGLR_ACTDE Polygalacturonase OS=Actinidia deliciosa OX=3627

PE=2 SV=1 map00040

Dioal.13G045800.1 OG0000021 Orthogroups

sp|C0LGG8|Y5343_ARATH Probable LRR receptor-like serine/threonine-protein

kinase At1g53430 OS=Arabidopsis thaliana OX=3702 GN=At1g53430 PE=1 SV=1 map00040

| Dioal.13G046000.1 | EC:3.2.1.15 | OG0000021 | Genome Annotation | sp\|C0LGG8\|Y5343_ARATH Probable LRR receptor-like serine/threonine-protein kinase At1g53430 OS=Arabidopsis thaliana OX=3702 GN=At1g53430 PE=1 SV=1 | map00040 |
| --- | --- | --- | --- | --- | --- |
| Dioal.13G046100.1 Dioal.13G053400.1 | EC:5.3.1.5 | OG0000021 OG0083049 | Orthogroups Genome Annotation | sp\|C0LGG9\|Y5344_ARATH Probable LRR receptor-like serine/threonine-protein kinase At1g53440 OS=Arabidopsis thaliana OX=3702 GN=At1g53440 PE=2 SV=2 sp\|Q40082\|XYLA_HORVU Xylose isomerase OS=Hordeum vulgare OX=4513 GN=XYLA PE=1 SV=1 | map00040 map00040 |
| Dioal.13G055900.1 Dioal.13G075500.1 Dioal.14G015100.1 Dioal.14G020300.1 Dioal.14G020400.1 Dioal.14G020500.1 Dioal.14G020700.1 Dioal.14G020800.1 Dioal.14G020900.1 Dioal.14G021000.1  Dioal.14G021100.1 | EC:4.2.2.2 | OG0030054 OG0000010 OG0000010 OG0000010 OG0000010 OG0000010 OG0000010 OG0000010 OG0000010 OG0000010  OG0000010 | Orthogroups Orthogroups Orthogroups Orthogroups Orthogroups Orthogroups Orthogroups Orthogroups Orthogroups Orthogroups  Orthogroups | A0A8B9A1R6_PHODC\|DNA polymerase  sp\|Q6JN46\|EIX2_SOLLC Receptor-like protein EIX2 OS=Solanum lycopersicum OX=4081 GN=EIX2 PE=1 SV=2  sp\|Q6JN46\|EIX2_SOLLC Receptor-like protein EIX2 OS=Solanum lycopersicum OX=4081 GN=EIX2 PE=1 SV=2  sp\|Q6JN46\|EIX2_SOLLC Receptor-like protein EIX2 OS=Solanum lycopersicum OX=4081 GN=EIX2 PE=1 SV=2  sp\|Q6JN46\|EIX2_SOLLC Receptor-like protein EIX2 OS=Solanum lycopersicum OX=4081 GN=EIX2 PE=1 SV=2  sp\|Q6JN46\|EIX2_SOLLC Receptor-like protein EIX2 OS=Solanum lycopersicum OX=4081 GN=EIX2 PE=1 SV=2  sp\|Q6JN46\|EIX2_SOLLC Receptor-like protein EIX2 OS=Solanum lycopersicum OX=4081 GN=EIX2 PE=1 SV=2  sp\|Q6JN46\|EIX2_SOLLC Receptor-like protein EIX2 OS=Solanum lycopersicum OX=4081 GN=EIX2 PE=1 SV=2  sp\|Q6JN46\|EIX2_SOLLC Receptor-like protein EIX2 OS=Solanum lycopersicum OX=4081 GN=EIX2 PE=1 SV=2  sp\|Q6JN46\|EIX2_SOLLC Receptor-like protein EIX2 OS=Solanum lycopersicum OX=4081 GN=EIX2 PE=1 SV=2  sp\|Q6JN46\|EIX2_SOLLC Receptor-like protein EIX2 OS=Solanum lycopersicum  OX=4081 GN=EIX2 PE=1 SV=2 | map00040 map00040 map00040 map00040 map00040 map00040 map00040 map00040 map00040 map00040  map00040 |
| Dioal.14G021200.1 Dioal.14G022100.1 |  | OG0000010 OG0000048 | Orthogroups Genome Annotation | sp\|Q6JN46\|EIX2_SOLLC Receptor-like protein EIX2 OS=Solanum lycopersicum OX=4081 GN=EIX2 PE=1 SV=2  sp\|Q944R1\|PLY15_ARATH Probable pectate lyase 15 OS=Arabidopsis thaliana  OX=3702 GN=At4g13710 PE=2 SV=1 | map00040 map00040 |

Dioal.14G022400.1 EC:4.2.2.2 OG0000048 Genome Annotation Dioal.14G023000.1 OG0000010 Orthogroups

Dioal.14G023100.1 OG0000010 Orthogroups

Dioal.14G023300.1 OG0000010 Orthogroups

Dioal.14G023400.1 OG0000010 Orthogroups

Dioal.14G023500.1 OG0000010 Orthogroups

Dioal.14G023700.1 OG0000010 Orthogroups

sp|Q9SCP2|PLY12_ARATH Probable pectate lyase 12 OS=Arabidopsis thaliana

OX=3702 GN=At3g53190 PE=2 SV=2 map00040

sp|Q6JN47|EIX1_SOLLC Receptor-like protein EIX1 OS=Solanum lycopersicum

OX=4081 GN=EIX1 PE=2 SV=2 map00040

sp|Q6JN46|EIX2_SOLLC Receptor-like protein EIX2 OS=Solanum lycopersicum

OX=4081 GN=EIX2 PE=1 SV=2 map00040

sp|Q6JN46|EIX2_SOLLC Receptor-like protein EIX2 OS=Solanum lycopersicum

OX=4081 GN=EIX2 PE=1 SV=2 map00040

sp|Q6JN46|EIX2_SOLLC Receptor-like protein EIX2 OS=Solanum lycopersicum

OX=4081 GN=EIX2 PE=1 SV=2 map00040

sp|Q6JN46|EIX2_SOLLC Receptor-like protein EIX2 OS=Solanum lycopersicum

OX=4081 GN=EIX2 PE=1 SV=2 map00040

sp|Q6JN46|EIX2_SOLLC Receptor-like protein EIX2 OS=Solanum lycopersicum

OX=4081 GN=EIX2 PE=1 SV=2 map00040

Dioal.14G078000.1 EC:2.4.1.17 OG0000490 Genome Annotation Dioal.14G083600.1 EC:3.2.1.15 OG0000322 Genome Annotation Dioal.14G118200.1 OG0000021 Orthogroups Dioal.14G122300.1 EC:3.1.1.11 OG0000009 Genome Annotation

sp|Q9LSB1|GUX1_ARATH UDP-glucuronate:xylan alpha-glucuronosyltransferase

1 OS=Arabidopsis thaliana OX=3702 GN=GUX1 PE=2 SV=1 map00040 sp|P48979|PGLR_PRUPE Polygalacturonase OS=Prunus persica OX=3760 PE=2

SV=1 map00040

sp|Q93YN1|CRPK1_ARATH Cold-responsive protein kinase 1 OS=Arabidopsis

thaliana OX=3702 GN=CRPK1 PE=1 SV=1 map00040

sp|O04887|PME2_CITSI Pectinesterase 2 OS=Citrus sinensis OX=2711 GN=PECS-

2.1 PE=2 SV=1 map00040

Dioal.14G140600.1 EC:3.1.1.11 OG0000198 Genome Annotation sp|P17407|21KD_DAUCA 21 kDa protein OS=Daucus carota OX=4039 PE=2 SV=1 map00040

Dioal.14G142100.1 EC:3.1.1.11 OG0000198 Genome Annotation sp|P17407|21KD_DAUCA 21 kDa protein OS=Daucus carota OX=4039 PE=2 SV=1 map00040

sp|Q8L608|HPSE2_ARATH Heparanase-like protein 2 OS=Arabidopsis thaliana

Dioal.15G010900.1 EC:3.2.1.167 EC OG0001270 Genome Annotation Dioal.15G011000.1 EC:3.2.1.167 EC OG0001270 Genome Annotation Dioal.15G084300.1 EC:3.2.1.15 OG0001060 Genome Annotation

OX=3702 GN=At5g61250 PE=2 SV=1 map00040

sp|Q8L608|HPSE2_ARATH Heparanase-like protein 2 OS=Arabidopsis thaliana

OX=3702 GN=At5g61250 PE=2 SV=1 map00040

sp|P35336|PGLR_ACTDE Polygalacturonase OS=Actinidia deliciosa OX=3627

PE=2 SV=1 map00040

| Dioal.15G084400.1 | EC:3.2.1.15 | OG0001060 | Genome Annotation | sp\|P35336\|PGLR_ACTDE Polygalacturonase OS=Actinidia deliciosa OX=3627  PE=2 SV=1 | map00040 |
| --- | --- | --- | --- | --- | --- |
| Dioal.15G084500.1 | EC:3.2.1.15 | OG0001060 | Genome Annotation | sp\|P35336\|PGLR_ACTDE Polygalacturonase OS=Actinidia deliciosa OX=3627  PE=2 SV=1 | map00040 |
| Dioal.15G084600.1 | EC:3.2.1.15 | OG0001060 | Genome Annotation | sp\|P35336\|PGLR_ACTDE Polygalacturonase OS=Actinidia deliciosa OX=3627  PE=2 SV=1 | map00040 |
| Dioal.15G114300.1 | EC:3.1.1.11 | OG0000009 | Genome Annotation | sp\|P09607\|PME21_SOLLC Pectinesterase 2.1 OS=Solanum lycopersicum  OX=4081 GN=PME2.1 PE=2 SV=2 | map00040 |
| Dioal.15G114400.1 | EC:3.1.1.11 | OG0000009 | Genome Annotation | sp\|P09607\|PME21_SOLLC Pectinesterase 2.1 OS=Solanum lycopersicum  OX=4081 GN=PME2.1 PE=2 SV=2 | map00040 |
| Dioal.15G114500.1 | EC:3.1.1.11 | OG0000009 | Genome Annotation | sp\|Q96575\|PME22_SOLLC Pectinesterase 2.2 OS=Solanum lycopersicum  OX=4081 GN=PME2.2 PE=3 SV=1 | map00040 |
| Dioal.16G016100.1 Dioal.16G076300.1 Dioal.16G090700.1 | EC:3.2.1.15 | OG0000666 OG0003721 OG0000086 | Orthogroups Orthogroups Genome Annotation | sp\|Q8GT41\|PLA1_PLAAC Putative invertase inhibitor OS=Platanus acerifolia OX=140101 PE=1 SV=1  A0A8B7CV80_PHODC\|leucine-rich repeat receptor-like kinase protein FLORAL ORGAN NUMBER1  sp\|P35339\|PGLR3_MAIZE Exopolygalacturonase OS=Zea mays OX=4577  GN=PG2C PE=2 SV=1 | map00040 map00040 map00040 |
| Dioal.17G001900.1 | EC:3.2.1.15 | OG0000546 | Genome Annotation | sp\|Q9SFB7\|QRT2_ARATH Polygalacturonase QRT2 OS=Arabidopsis thaliana  OX=3702 GN=QRT2 PE=1 SV=2 | map00040 |
| Dioal.17G002000.1 | EC:3.2.1.15 | OG0000546 | Genome Annotation | sp\|Q9LW07\|PGLR3_ARATH Probable polygalacturonase At3g15720  OS=Arabidopsis thaliana OX=3702 GN=At3g15720 PE=3 SV=1 | map00040 |
| Dioal.17G002100.1 | EC:3.2.1.15 | OG0000546 | Genome Annotation | sp\|Q9SFB7\|QRT2_ARATH Polygalacturonase QRT2 OS=Arabidopsis thaliana  OX=3702 GN=QRT2 PE=1 SV=2 | map00040 |
| Dioal.17G007700.1 Dioal.17G007800.1 |  | OG0000010 OG0000010 | Orthogroups Orthogroups | sp\|Q6JN46\|EIX2_SOLLC Receptor-like protein EIX2 OS=Solanum lycopersicum OX=4081 GN=EIX2 PE=1 SV=2  sp\|Q6JN46\|EIX2_SOLLC Receptor-like protein EIX2 OS=Solanum lycopersicum OX=4081 GN=EIX2 PE=1 SV=2 | map00040 map00040 |
| Dioal.17G020200.1 | EC:4.2.2.2 | OG0000048 | Genome Annotation | sp\|P40973\|PLY_LILLO Pectate lyase OS=Lilium longiflorum OX=4690 PE=2 SV=1 | map00040 |
| Dioal.17G048900.1 | EC:3.1.1.11 | OG0000009 | Genome Annotation | sp\|Q9M3B0\|PME34_ARATH Probable pectinesterase/pectinesterase inhibitor 34  OS=Arabidopsis thaliana OX=3702 GN=PME34 PE=2 SV=1 | map00040 |
| Dioal.17G121600.1 | EC:3.2.1.15 | OG0004369 | Genome Annotation | sp\|Q94AJ5\|PGLR5_ARATH Probable polygalacturonase At1g80170  OS=Arabidopsis thaliana OX=3702 GN=At1g80170 PE=1 SV=1 | map00040 |

| Dioal.18G002100.1 | EC:3.1.1.11 | OG0000009 | Genome Annotation | sp\|Q43062\|PME_PRUPE Pectinesterase/pectinesterase inhibitor PPE8B  OS=Prunus persica OX=3760 PE=2 SV=1 | map00040 |
| --- | --- | --- | --- | --- | --- |
| Dioal.18G002200.1 | EC:3.1.1.11 EC: | OG0000009 | Genome Annotation | sp\|O48711\|PME12_ARATH Probable pectinesterase/pectinesterase inhibitor 12  OS=Arabidopsis thaliana OX=3702 GN=PME12 PE=2 SV=1 | map00040 |
| Dioal.18G002700.1 | EC:3.1.1.11 | OG0000009 | Genome Annotation | sp\|Q9SG77\|PME24_ARATH Putative pectinesterase/pectinesterase inhibitor 24  OS=Arabidopsis thaliana OX=3702 GN=PME24 PE=3 SV=1 | map00040 |
| Dioal.18G002800.1 | EC:3.1.1.11 | OG0000009 | Genome Annotation | sp\|Q43111\|PME3_PHAVU Pectinesterase 3 OS=Phaseolus vulgaris OX=3885  GN=MPE3 PE=2 SV=1 | map00040 |
| Dioal.18G002900.1 | EC:3.1.1.11 | OG0000009 | Genome Annotation | sp\|Q9SKX2\|PME16_ARATH Probable pectinesterase/pectinesterase inhibitor 16  OS=Arabidopsis thaliana OX=3702 GN=PME16 PE=2 SV=1 | map00040 |
| Dioal.18G021200.1 | EC:2.4.1.17 | OG0000490 | Genome Annotation | sp\|Q8GWW4\|GUX2_ARATH UDP-glucuronate:xylan alpha- glucuronosyltransferase 2 OS=Arabidopsis thaliana OX=3702 GN=GUX2 PE=2  SV=1 | map00040 |
| Dioal.18G034400.1 | EC:5.3.1.5 | OG0030054 | Genome Annotation | sp\|Q9FKK7\|XYLA_ARATH Xylose isomerase OS=Arabidopsis thaliana OX=3702  GN=XYLA PE=2 SV=2 | map00040 |
| Dioal.18G035000.1  Dioal.18G035700.1 | EC:5.3.1.5 | OG0030054  OG0025251 | Orthogroups  Genome Annotation | A0A8B9A1R6_PHODC\|DNA polymerase  sp\|Q9FKK7\|XYLA_ARATH Xylose isomerase OS=Arabidopsis thaliana OX=3702 GN=XYLA PE=2 SV=2 | map00040  map00040 |
| Dioal.18G036300.1 | EC:5.3.1.5 | OG0018886 | Genome Annotation | sp\|Q40082\|XYLA_HORVU Xylose isomerase OS=Hordeum vulgare OX=4513  GN=XYLA PE=1 SV=1 | map00040 |
| Dioal.18G040500.1 Dioal.18G047000.1 | EC:3.1.1.11 | OG0000010 OG0000198 | Orthogroups Genome Annotation | sp\|Q6JN47\|EIX1_SOLLC Receptor-like protein EIX1 OS=Solanum lycopersicum OX=4081 GN=EIX1 PE=2 SV=2  sp\|Q9SI72\|PMEI9_ARATH Pectinesterase inhibitor 9 OS=Arabidopsis thaliana  OX=3702 GN=PMEI9 PE=2 SV=1 | map00040 map00040 |
| Dioal.18G051400.1 | EC:3.1.1.11 | OG0000009 | Genome Annotation | sp\|O49298\|PME6_ARATH Probable pectinesterase/pectinesterase inhibitor 6  OS=Arabidopsis thaliana OX=3702 GN=PME6 PE=2 SV=1 | map00040 |
| Dioal.18G051500.1 | EC:3.1.1.11 | OG0000009 | Genome Annotation | sp\|O49298\|PME6_ARATH Probable pectinesterase/pectinesterase inhibitor 6  OS=Arabidopsis thaliana OX=3702 GN=PME6 PE=2 SV=1 | map00040 |
| Dioal.18G051600.1 | EC:3.1.1.11 | OG0000009 | Genome Annotation | sp\|O49298\|PME6_ARATH Probable pectinesterase/pectinesterase inhibitor 6  OS=Arabidopsis thaliana OX=3702 GN=PME6 PE=2 SV=1 | map00040 |
| Dioal.18G067500.1 | EC:1.1.1.179 | OG0001528 | Genome Annotation | sp\|Q9SZ83\|Y4967_ARATH Uncharacterized oxidoreductase At4g09670  OS=Arabidopsis thaliana OX=3702 GN=At4g09670 PE=1 SV=1 | map00040 |
| Dioal.18G067600.1 | EC:1.1.1.179 | OG0001528 | Genome Annotation | sp\|Q9SZ83\|Y4967_ARATH Uncharacterized oxidoreductase At4g09670  OS=Arabidopsis thaliana OX=3702 GN=At4g09670 PE=1 SV=1 | map00040 |

Dioal.18G074000.1 EC:3.1.1.11 OG0000009 Genome Annotation Dioal.18G093900.1 EC:2.7.7.9 OG0009535 Genome Annotation Dioal.19G014900.1 EC:3.2.1.167 EC OG0001270 Genome Annotation Dioal.19G022000.1 EC:3.1.1.11 OG0006796 Genome Annotation Dioal.19G045900.1 EC:3.1.1.11 OG0000198 Genome Annotation Dioal.19G078300.1 OG0000021 Orthogroups

Dioal.19G105500.1 OG0000010 Orthogroups

Dioal.19G105600.1 OG0000010 Orthogroups

Dioal.19G134100.1 OG0003721 Orthogroups

Dioal.19G140100.1 OG0000021 Orthogroups Dioal.19G149200.1 EC:2.7.7.23 EC: OG0005426 Genome Annotation Dioal.19G149600.1 EC:3.1.1.11 OG0003143 Genome Annotation Dioal.19G149900.1 EC:3.2.1.15 OG0002692 Genome Annotation

sp|Q43111|PME3_PHAVU Pectinesterase 3 OS=Phaseolus vulgaris OX=3885

GN=MPE3 PE=2 SV=1 map00040

sp|F4IY62|UGPA3_ARATH UTP--glucose-1-phosphate uridylyltransferase 3,

chloroplastic OS=Arabidopsis thaliana OX=3702 GN=UGP3 PE=1 SV=1 map00040 sp|Q9FF10|HPSE1_ARATH Heparanase-like protein 1 OS=Arabidopsis thaliana

OX=3702 GN=At5g07830 PE=2 SV=1 map00040

sp|Q9LVQ0|PME31_ARATH Pectinesterase 31 OS=Arabidopsis thaliana OX=3702 GN=PME31 PE=1 SV=1 map00040

sp|Q9SI72|PMEI9_ARATH Pectinesterase inhibitor 9 OS=Arabidopsis thaliana

OX=3702 GN=PMEI9 PE=2 SV=1 map00040

sp|Q93YN1|CRPK1_ARATH Cold-responsive protein kinase 1 OS=Arabidopsis

thaliana OX=3702 GN=CRPK1 PE=1 SV=1 map00040

sp|Q6JN46|EIX2_SOLLC Receptor-like protein EIX2 OS=Solanum lycopersicum

OX=4081 GN=EIX2 PE=1 SV=2 map00040

sp|Q6JN46|EIX2_SOLLC Receptor-like protein EIX2 OS=Solanum lycopersicum

OX=4081 GN=EIX2 PE=1 SV=2 map00040

sp|Q42371|ERECT_ARATH LRR receptor-like serine/threonine-protein kinase

ERECTA OS=Arabidopsis thaliana OX=3702 GN=ERECTA PE=1 SV=1 map00040 sp|Q93YN1|CRPK1_ARATH Cold-responsive protein kinase 1 OS=Arabidopsis

thaliana OX=3702 GN=CRPK1 PE=1 SV=1 map00040

sp|O64765|UAP2_ARATH UDP-N-acetylglucosamine diphosphorylase 2

OS=Arabidopsis thaliana OX=3702 GN=GLCNAC1PUT2 PE=1 SV=1 map00040 sp|O23038|PME8_ARATH Probable pectinesterase 8 OS=Arabidopsis thaliana

OX=3702 GN=PME8 PE=2 SV=2 map00040

sp|O49432|QRT3_ARATH Polygalacturonase QRT3 OS=Arabidopsis thaliana

OX=3702 GN=QRT3 PE=2 SV=1 map00040

Dioal.19G150200.1 EC:3.1.1.11 OG0000021 Orthogroups

sp|C0LGH2|Y1561_ARATH Probable LRR receptor-like serine/threonine-protein

kinase At1g56130 OS=Arabidopsis thaliana OX=3702 GN=At1g56130 PE=2 SV=2 map00040

Dioal.19G150300.1 OG0000021 Orthogroups

sp|C0LGH2|Y1561_ARATH Probable LRR receptor-like serine/threonine-protein

kinase At1g56130 OS=Arabidopsis thaliana OX=3702 GN=At1g56130 PE=2 SV=2 map00040

| Dioal.19G150500.1 |  | OG0000021 | Orthogroups | sp\|C0LGH2\|Y1561_ARATH Probable LRR receptor-like serine/threonine-protein kinase At1g56130 OS=Arabidopsis thaliana OX=3702 GN=At1g56130 PE=2 SV=2 | map00040 |
| --- | --- | --- | --- | --- | --- |
| Dioal.19G150600.1 |  | OG0000021 | Orthogroups | sp\|C0LGH2\|Y1561_ARATH Probable LRR receptor-like serine/threonine-protein kinase At1g56130 OS=Arabidopsis thaliana OX=3702 GN=At1g56130 PE=2 SV=2 | map00040 |
| Dioal.19G150700.1 Dioal.19G181200.1 Dioal.19G187400.1 | EC:3.1.1.11 | OG0000021 OG0000009 OG0001942 | Orthogroups Genome Annotation Genome Annotation | sp\|C0LGH2\|Y1561_ARATH Probable LRR receptor-like serine/threonine-protein kinase At1g56130 OS=Arabidopsis thaliana OX=3702 GN=At1g56130 PE=2 SV=2 sp\|O81301\|PME40_ARATH Probable pectinesterase/pectinesterase inhibitor 40 OS=Arabidopsis thaliana OX=3702 GN=PME40 PE=2 SV=1 sp\|Q8VYZ3\|PME53_ARATH Probable pectinesterase 53 OS=Arabidopsis thaliana OX=3702 GN=PME53 PE=2 SV=1 | map00040 map00040 map00040 |
| Dioal.20G018900.1 | EC:5.3.1.5 | OG0018886 | Genome Annotation | sp\|Q40082\|XYLA_HORVU Xylose isomerase OS=Hordeum vulgare OX=4513  GN=XYLA PE=1 SV=1 | map00040 |
| Dioal.20G033700.1 | EC:3.2.1.15 | OG0002811 | Genome Annotation | sp\|Q949Z1\|PGLR4_ARATH Polygalacturonase At1g48100 OS=Arabidopsis  thaliana OX=3702 GN=At1g48100 PE=2 SV=1 | map00040 |
| Dioal.20G035100.1 | EC:3.1.1.11 | OG0005257 | Genome Annotation | sp\|Q9SIJ9\|PME11_ARATH Putative pectinesterase 11 OS=Arabidopsis thaliana  OX=3702 GN=PME11 PE=3 SV=1 | map00040 |
| Dioal.20G035200.1 | EC:3.1.1.11 | OG0005257 | Genome Annotation | sp\|Q9SIJ9\|PME11_ARATH Putative pectinesterase 11 OS=Arabidopsis thaliana OX=3702 GN=PME11 PE=3 SV=1 | map00040 |
| Dioal.20G035900.1 Dioal.20G042200.1 | EC:3.1.1.11 | OG0000021 OG0001942 | Orthogroups Genome Annotation | sp\|C0LGG9\|Y5344_ARATH Probable LRR receptor-like serine/threonine-protein kinase At1g53440 OS=Arabidopsis thaliana OX=3702 GN=At1g53440 PE=2 SV=2 sp\|Q8VYZ3\|PME53_ARATH Probable pectinesterase 53 OS=Arabidopsis thaliana OX=3702 GN=PME53 PE=2 SV=1 | map00040 map00040 |
| Dioal.20G055000.1 |  | OG0000021 | Orthogroups | sp\|C0LGG8\|Y5343_ARATH Probable LRR receptor-like serine/threonine-protein kinase At1g53430 OS=Arabidopsis thaliana OX=3702 GN=At1g53430 PE=1 SV=1 | map00040 |
| Dioal.20G055100.1 |  | OG0000021 | Orthogroups | sp\|C0LGG9\|Y5344_ARATH Probable LRR receptor-like serine/threonine-protein kinase At1g53440 OS=Arabidopsis thaliana OX=3702 GN=At1g53440 PE=2 SV=2 | map00040 |

| Dioal.20G055200.1 | OG0000021 | Orthogroups | sp\|C0LGG8\|Y5343_ARATH Probable LRR receptor-like serine/threonine-protein kinase At1g53430 OS=Arabidopsis thaliana OX=3702 GN=At1g53430 PE=1 SV=1 | map00040 |
| --- | --- | --- | --- | --- |
| Dioal.20G055300.1 | OG0000021 | Orthogroups | sp\|C0LGG9\|Y5344_ARATH Probable LRR receptor-like serine/threonine-protein kinase At1g53440 OS=Arabidopsis thaliana OX=3702 GN=At1g53440 PE=2 SV=2 | map00040 |
| Dioal.20G055400.1 | OG0000021 | Orthogroups | sp\|C0LGG8\|Y5343_ARATH Probable LRR receptor-like serine/threonine-protein kinase At1g53430 OS=Arabidopsis thaliana OX=3702 GN=At1g53430 PE=1 SV=1 | map00040 |
| Dioal.20G055500.1 | OG0000021 | Orthogroups | sp\|C0LGG9\|Y5344_ARATH Probable LRR receptor-like serine/threonine-protein kinase At1g53440 OS=Arabidopsis thaliana OX=3702 GN=At1g53440 PE=2 SV=2 | map00040 |
| Dioal.20G055700.1 | OG0000021 | Orthogroups | sp\|C0LGG9\|Y5344_ARATH Probable LRR receptor-like serine/threonine-protein kinase At1g53440 OS=Arabidopsis thaliana OX=3702 GN=At1g53440 PE=2 SV=2 | map00040 |
| Dioal.20G055800.1 | OG0000021 | Orthogroups | sp\|C0LGG8\|Y5343_ARATH Probable LRR receptor-like serine/threonine-protein kinase At1g53430 OS=Arabidopsis thaliana OX=3702 GN=At1g53430 PE=1 SV=1 | map00040 |
| Dioal.20G055900.1 | OG0000021 | Orthogroups | sp\|C0LGG8\|Y5343_ARATH Probable LRR receptor-like serine/threonine-protein kinase At1g53430 OS=Arabidopsis thaliana OX=3702 GN=At1g53430 PE=1 SV=1 | map00040 |
| Dioal.20G056000.1 | OG0000021 | Orthogroups | sp\|C0LGG8\|Y5343_ARATH Probable LRR receptor-like serine/threonine-protein kinase At1g53430 OS=Arabidopsis thaliana OX=3702 GN=At1g53430 PE=1 SV=1 | map00040 |
| Dioal.20G056100.1 | OG0000021 | Orthogroups | sp\|C0LGG9\|Y5344_ARATH Probable LRR receptor-like serine/threonine-protein kinase At1g53440 OS=Arabidopsis thaliana OX=3702 GN=At1g53440 PE=2 SV=2 | map00040 |
| Dioal.20G056200.1 | OG0000021 | Orthogroups | sp\|C0LGG8\|Y5343_ARATH Probable LRR receptor-like serine/threonine-protein kinase At1g53430 OS=Arabidopsis thaliana OX=3702 GN=At1g53430 PE=1 SV=1 | map00040 |
| Dioal.20G056300.1 | OG0000021 | Orthogroups | sp\|C0LGG8\|Y5343_ARATH Probable LRR receptor-like serine/threonine-protein kinase At1g53430 OS=Arabidopsis thaliana OX=3702 GN=At1g53430 PE=1 SV=1 | map00040 |

Dioal.20G056400.1 OG0000021 Orthogroups

sp|C0LGG8|Y5343_ARATH Probable LRR receptor-like serine/threonine-protein

kinase At1g53430 OS=Arabidopsis thaliana OX=3702 GN=At1g53430 PE=1 SV=1 map00040

Dioal.01G018700.1 EC:3.2.1.2 OG0004453 Genome Annotation Dioal.01G070900.1 EC:3.2.1.4 OG0002962 Genome Annotation Dioal.02G090200.1 OG0000179 Orthogroups Dioal.02G098800.1 EC:5.4.2.2 EC:5. OG0007280 Genome Annotation

Dioal.03G018100.1 EC:2.4.1.15 EC: OG0000282 Genome Annotation

sp|O80831|BAM7_ARATH Beta-amylase 7 OS=Arabidopsis thaliana OX=3702

GN=BAM7 PE=1 SV=2 map00500

sp|P05522|GUN1_PERAE Endoglucanase 1 OS=Persea americana OX=3435

GN=CEL1 PE=2 SV=1 map00500

sp|Q84JA6|CESA4_ARATH Cellulose synthase A catalytic subunit 4 [UDP-forming] OS=Arabidopsis thaliana OX=3702 GN=CESA4 PE=1 SV=1 map00500 A0A8B9AHL2_PHODC|phosphoglucomutase (alpha-D-glucose-1,6-bisphosphate-

dependent) map00500

sp|Q9LRA7|TPS9_ARATH Probable alpha,alpha-trehalose-phosphate synthase

[UDP-forming] 9 OS=Arabidopsis thaliana OX=3702 GN=TPS9 PE=2 SV=1 map00500

Dioal.03G020000.1 EC:2.7.1.1 OG0007187 Genome Annotation A0A6I9Q8B2_ELAGV|AFG1-like ATPase map00500

sp|Q3B724|CALS5_ARATH Callose synthase 5 OS=Arabidopsis thaliana OX=3702

Dioal.03G027600.1 EC:2.4.1.34 OG0000252 Genome Annotation Dioal.03G073600.1 EC:2.7.7.27 OG0003708 Genome Annotation Dioal.04G001400.1 EC:5.3.1.9 OG0008581 Genome Annotation Dioal.04G013300.1 EC:2.7.1.4 OG0001115 Genome Annotation Dioal.04G020700.1 EC:2.7.1.4 OG0001115 Genome Annotation

Dioal.04G021700.1 EC:2.7.7.27 OG0003708 Genome Annotation Dioal.04G091400.1 EC:2.4.1.34 OG0000252 Genome Annotation Dioal.04G092600.1 EC:3.2.1.4 OG0000967 Genome Annotation Dioal.04G095100.1 EC:2.4.1.15 EC: OG0000282 Genome Annotation

GN=CALS5 PE=1 SV=1 map00500

sp|Q9M462|GLGS_BRANA Glucose-1-phosphate adenylyltransferase small

subunit, chloroplastic OS=Brassica napus OX=3708 GN=AGPS1 PE=2 SV=1 map00500 sp|P54237|G6PI1_CLAMI Glucose-6-phosphate isomerase, cytosolic 1 OS=Clarkia

mildrediae OX=49756 GN=PGIC1 PE=3 SV=1 map00500 sp|Q6XZ79|SCRK1_MAIZE Fructokinase-1 OS=Zea mays OX=4577 GN=FRK1

PE=1 SV=1 map00500

sp|A2YQL4|SCRK2_ORYSI Fructokinase-2 OS=Oryza sativa subsp. indica

OX=39946 GN=FRK2 PE=1 SV=2 map00500

sp|F4I8U2|APS2L_ARATH Inactive glucose-1-phosphate adenylyltransferase small subunit 2, chloroplastic OS=Arabidopsis thaliana OX=3702 GN=APS2 PE=1

SV=1 map00500

sp|Q9SHJ3|CALS7_ARATH Callose synthase 7 OS=Arabidopsis thaliana OX=3702

GN=CALS7 PE=3 SV=3 map00500

sp|C0HLA0|GH5FP_CHAOB Glycosyl hydrolase 5 family protein

OS=Chamaecyparis obtusa OX=13415 PE=1 SV=1 map00500 sp|Q9LMI0|TPS7_ARATH Probable alpha,alpha-trehalose-phosphate synthase

[UDP-forming] 7 OS=Arabidopsis thaliana OX=3702 GN=TPS7 PE=1 SV=1 map00500

Dioal.04G101300.1 EC:2.7.1.1 OG0000334 Genome Annotation Dioal.04G130300.1 EC:3.2.1.4 OG0002803 Genome Annotation Dioal.04G158700.1 EC:2.4.1.15 EC: OG0000282 Genome Annotation Dioal.04G168200.1 EC:3.2.1.2 OG0002084 Genome Annotation Dioal.04G184200.1 EC:3.1.3.12 OG0002034 Genome Annotation Dioal.05G025500.1 EC:3.2.1.4 OG0012518 Genome Annotation Dioal.05G053000.1 EC:2.7.1.4 OG0001115 Genome Annotation Dioal.05G053400.1 EC:2.7.1.4 OG0001115 Genome Annotation Dioal.05G055600.1 EC:3.2.1.4 OG0007353 Genome Annotation Dioal.05G075200.1 EC:3.2.1.4 OG0000967 Genome Annotation Dioal.05G075500.1 EC:3.2.1.4 OG0000967 Genome Annotation Dioal.05G075600.1 EC:3.2.1.4 OG0000967 Genome Annotation Dioal.05G096100.1 EC:2.7.1.1 OG0000334 Genome Annotation Dioal.05G113700.1 EC:3.2.1.4 OG0005413 Genome Annotation Dioal.05G114400.1 EC:3.1.3.12 OG0000588 Genome Annotation Dioal.05G128700.1 EC:3.2.1.4 OG0000967 Genome Annotation

sp|Q9SEK3|HXK1_SPIOL Hexokinase-1 OS=Spinacia oleracea OX=3562 GN=HXK1

PE=2 SV=1 map00500

sp|Q9CAC1|GUN8_ARATH Endoglucanase 8 OS=Arabidopsis thaliana OX=3702

GN=CEL1 PE=2 SV=1 map00500

sp|O23617|TPS5_ARATH Alpha,alpha-trehalose-phosphate synthase [UDP-

forming] 5 OS=Arabidopsis thaliana OX=3702 GN=TPS5 PE=1 SV=2 map00500 sp|O23553|BAM3_ARATH Beta-amylase 3, chloroplastic OS=Arabidopsis thaliana

OX=3702 GN=BAM3 PE=1 SV=3 map00500

sp|Q9SUW0|TPPG_ARATH Probable trehalose-phosphate phosphatase G

OS=Arabidopsis thaliana OX=3702 GN=TPPG PE=2 SV=1 map00500 sp|Q38890|GUN25_ARATH Endoglucanase 25 OS=Arabidopsis thaliana OX=3702

GN=KOR PE=1 SV=1 map00500

sp|A2YQL4|SCRK2_ORYSI Fructokinase-2 OS=Oryza sativa subsp. indica

OX=39946 GN=FRK2 PE=1 SV=2 map00500

sp|A2YQL4|SCRK2_ORYSI Fructokinase-2 OS=Oryza sativa subsp. indica

OX=39946 GN=FRK2 PE=1 SV=2 map00500

sp|Q9C9H5|GUN9_ARATH Endoglucanase 9 OS=Arabidopsis thaliana OX=3702

GN=CEL3 PE=1 SV=1 map00500

sp|C0HLA0|GH5FP_CHAOB Glycosyl hydrolase 5 family protein

OS=Chamaecyparis obtusa OX=13415 PE=1 SV=1 map00500 sp|C0HLA0|GH5FP_CHAOB Glycosyl hydrolase 5 family protein

OS=Chamaecyparis obtusa OX=13415 PE=1 SV=1 map00500 sp|C0HLA0|GH5FP_CHAOB Glycosyl hydrolase 5 family protein

OS=Chamaecyparis obtusa OX=13415 PE=1 SV=1 map00500 sp|P93834|HXK2_ARATH Hexokinase-2 OS=Arabidopsis thaliana OX=3702

GN=HXK2 PE=1 SV=1 map00500

sp|Q42059|GUN6_ARATH Endoglucanase 6 OS=Arabidopsis thaliana OX=3702 GN=At1g64390 PE=2 SV=2 map00500

sp|Q6ZGP8|TPP4_ORYSJ Probable trehalose-phosphate phosphatase 4

OS=Oryza sativa subsp. japonica OX=39947 GN=TPP4 PE=2 SV=1 map00500 sp|C0HLA0|GH5FP_CHAOB Glycosyl hydrolase 5 family protein

OS=Chamaecyparis obtusa OX=13415 PE=1 SV=1 map00500

Dioal.05G145300.1 EC:2.4.1.245 OG0005016 Genome Annotation uncharacterized protein map00500

| Dioal.05G175900.1 | EC:3.2.1.4 | OG0001721 | Genome Annotation | sp\|Q7XUK4\|GUN12_ORYSJ Endoglucanase 12 OS=Oryza sativa subsp. japonica  OX=39947 GN=GLU3 PE=2 SV=2 | map00500 |
| --- | --- | --- | --- | --- | --- |
| Dioal.05G185800.1 | EC:5.4.2.2 | OG0008162 | Genome Annotation | sp\|Q9ZSQ4\|PGMC_POPTN Phosphoglucomutase, cytoplasmic OS=Populus  tremula OX=113636 GN=PGM1 PE=2 SV=1 | map00500 |
| Dioal.05G188500.1 | EC:3.1.3.12 | OG0000588 | Genome Annotation | sp\|Q6ZAL2\|TPP6_ORYSJ Probable trehalose-phosphate phosphatase 6 OS=Oryza  sativa subsp. japonica OX=39947 GN=TPP6 PE=2 SV=1 | map00500 |
| Dioal.05G189200.1 | EC:3.2.1.58 | OG0002985 | Genome Annotation | A0A8N4ID02_ELAGV\|probable glucan 1,3-beta-glucosidase | map00500 |
| Dioal.05G210400.1 | EC:3.1.3.12 | OG0000588 | Genome Annotation | sp\|Q6ZAL2\|TPP6_ORYSJ Probable trehalose-phosphate phosphatase 6 OS=Oryza  sativa subsp. japonica OX=39947 GN=TPP6 PE=2 SV=1 | map00500 |
| Dioal.05G211400.1 | EC:3.1.3.12 | OG0000588 | Genome Annotation | sp\|Q6ZAL2\|TPP6_ORYSJ Probable trehalose-phosphate phosphatase 6 OS=Oryza  sativa subsp. japonica OX=39947 GN=TPP6 PE=2 SV=1 | map00500 |
| Dioal.06G018300.1 | EC:3.1.3.24 | OG0003909 | Genome Annotation | sp\|Q5IH13\|SPP2_TOBAC Sucrose-phosphatase 2 OS=Nicotiana tabacum  OX=4097 GN=SPP2 PE=2 SV=1 | map00500 |
| Dioal.06G019000.1 | EC:3.1.3.24 | OG0003909 | Genome Annotation | sp\|Q5IH13\|SPP2_TOBAC Sucrose-phosphatase 2 OS=Nicotiana tabacum  OX=4097 GN=SPP2 PE=2 SV=1 | map00500 |
| Dioal.06G026700.1 Dioal.06G027400.1 | EC:2.7.7.27 | OG0019473 OG0019473 | Orthogroups Genome Annotation | sp\|F4HQA1\|PAF1_ARATH Protein PAF1 homolog OS=Arabidopsis thaliana OX=3702 GN=VIP2 PE=1 SV=1  sp\|F4HQA1\|PAF1_ARATH Protein PAF1 homolog OS=Arabidopsis thaliana  OX=3702 GN=VIP2 PE=1 SV=1 | map00500 map00500 |
| Dioal.06G028600.1 Dioal.06G028900.1 Dioal.06G029200.1 | EC:2.7.7.27 | OG0019473 OG0019473 OG0019473 | Orthogroups Orthogroups Genome Annotation | sp\|F4HQA1\|PAF1_ARATH Protein PAF1 homolog OS=Arabidopsis thaliana OX=3702 GN=VIP2 PE=1 SV=1  sp\|F4HQA1\|PAF1_ARATH Protein PAF1 homolog OS=Arabidopsis thaliana OX=3702 GN=VIP2 PE=1 SV=1  sp\|F4HQA1\|PAF1_ARATH Protein PAF1 homolog OS=Arabidopsis thaliana  OX=3702 GN=VIP2 PE=1 SV=1 | map00500 map00500 map00500 |
| Dioal.06G033200.1 Dioal.06G045200.1 | EC:3.2.1.1 | OG0005426 OG0001569 | Orthogroups Genome Annotation | sp\|Q940S3\|UAP1_ARATH UDP-N-acetylglucosamine diphosphorylase 1 OS=Arabidopsis thaliana OX=3702 GN=GLCNAC1PUT1 PE=1 SV=1 sp\|P27939\|AMY3C_ORYSJ Alpha-amylase isozyme 3C OS=Oryza sativa subsp.  japonica OX=39947 GN=AMY1.7 PE=2 SV=2 | map00500 map00500 |
| Dioal.06G045800.1 | EC:3.2.1.1 | OG0001569 | Genome Annotation | sp\|P27937\|AMY3B_ORYSJ Alpha-amylase isozyme 3B OS=Oryza sativa subsp.  japonica OX=39947 GN=AMY1.6 PE=2 SV=1 | map00500 |
| Dioal.06G055800.1 | EC:2.7.1.4 | OG0002795 | Genome Annotation | sp\|Q9C524\|SCRK6_ARATH Probable fructokinase-6, chloroplastic OS=Arabidopsis  thaliana OX=3702 GN=At1g66430 PE=2 SV=1 | map00500 |

Dioal.06G067700.1 EC:3.2.1.4 OG0008229 Genome Annotation

sp|O64890|GUN13_ARATH Endoglucanase 13 OS=Arabidopsis thaliana OX=3702 GN=At2g44550 PE=2 SV=1 map00500

Dioal.06G072700.1 EC:3.2.1.58 OG0010692 Genome Annotation A0A6I9SF01_ELAGV|probable glucan 1,3-beta-glucosidase A isoform X1 map00500

sp|Q652F9|GUN17_ORYSJ Endoglucanase 17 OS=Oryza sativa subsp. japonica

Dioal.06G081700.1 EC:3.2.1.4 OG0002803 Genome Annotation Dioal.06G084000.1 EC:2.4.1.15 EC: OG0000282 Genome Annotation

Dioal.07G000900.1 EC:2.7.7.27 OG0000839 Genome Annotation Dioal.07G026700.1 EC:3.2.1.4 OG0002962 Genome Annotation Dioal.07G051400.1 EC:3.2.1.68 OG0010793 Genome Annotation Dioal.07G064700.1 EC:2.7.1.1 OG0000334 Genome Annotation Dioal.07G067100.1 EC:2.4.1.25 OG0006855 Genome Annotation Dioal.07G095500.1 EC:3.2.1.4 OG0001721 Genome Annotation Dioal.08G008000.1 EC:3.2.1.1 OG0012351 Genome Annotation

OX=39947 GN=GLU13 PE=2 SV=1 map00500

sp|Q94AH8|TPS6_ARATH Alpha,alpha-trehalose-phosphate synthase [UDP-

forming] 6 OS=Arabidopsis thaliana OX=3702 GN=TPS6 PE=1 SV=2 map00500 sp|Q0D7I3|GLGL4_ORYSJ Glucose-1-phosphate adenylyltransferase large subunit

4, chloroplastic/amyloplastic OS=Oryza sativa subsp. japonica OX=39947

GN=AGPL4 PE=1 SV=1 map00500

sp|O81416|GUN17_ARATH Endoglucanase 17 OS=Arabidopsis thaliana OX=3702 GN=At4g02290 PE=2 SV=1 map00500

sp|Q8L735|ISOA2_ARATH Isoamylase 2, chloroplastic OS=Arabidopsis thaliana

OX=3702 GN=ISA2 PE=1 SV=2 map00500

sp|Q2KNB4|HXK3_ORYSJ Hexokinase-3 OS=Oryza sativa subsp. japonica

OX=39947 GN=HXK3 PE=2 SV=1 map00500

sp|Q69Q02|DPE2_ORYSJ 4-alpha-glucanotransferase DPE2 OS=Oryza sativa

subsp. japonica OX=39947 GN=DPE2 PE=2 SV=1 map00500 sp|P0C1U4|GUN9_ORYSJ Endoglucanase 9 OS=Oryza sativa subsp. japonica

OX=39947 GN=GLU1 PE=2 SV=1 map00500

sp|Q94A41|AMY3_ARATH Alpha-amylase 3, chloroplastic OS=Arabidopsis

thaliana OX=3702 GN=AMY3 PE=1 SV=1 map00500

Dioal.08G030400.1 EC:2.7.1.1 OG0005155 Genome Annotation A0A8B7D305_PHODC|N-acetyl-D-glucosamine kinase map00500

Dioal.08G041200.1 OG0000179 Orthogroups Dioal.08G081500.1 EC:3.2.1.4 OG0002953 Genome Annotation Dioal.08G088800.1 EC:3.2.1.68 OG0008556 Genome Annotation Dioal.08G089400.1 EC:2.7.7.10 EC: OG0007363 Genome Annotation Dioal.08G106000.1 OG0000179 Orthogroups

sp|Q69V23|CESA3_ORYSJ Probable cellulose synthase A catalytic subunit 3 [UDP-

forming] OS=Oryza sativa subsp. japonica OX=39947 GN=CESA3 PE=2 SV=1 map00500 sp|O48766|GUN11_ARATH Endoglucanase 11 OS=Arabidopsis thaliana OX=3702 GN=At2g32990 PE=2 SV=1 map00500

sp|D0TZF0|ISOA1_ORYSJ Isoamylase 1, chloroplastic OS=Oryza sativa subsp.

japonica OX=39947 GN=ISA1 PE=1 SV=1 map00500

sp|A2YGP6|USP_ORYSI UDP-sugar pyrophosphorylase OS=Oryza sativa subsp.

indica OX=39946 GN=USP PE=3 SV=2 map00500

sp|A2Z1C8|CESA9_ORYSI Cellulose synthase A catalytic subunit 9 [UDP-forming]

OS=Oryza sativa subsp. indica OX=39946 GN=CESA9 PE=2 SV=1 map00500

| Dioal.08G124800.1 | EC:3.2.1.58 | OG0000457 | Genome Annotation | sp\|A5JTQ3\|XYL2_MEDSV Beta-xylosidase/alpha-L-arabinofuranosidase 2 OS=Medicago sativa subsp. varia OX=36902 GN=Xyl2 PE=2 SV=1 | map00500 |
| --- | --- | --- | --- | --- | --- |
| Dioal.08G130500.1 |  | OG0000179 | Orthogroups | sp\|Q84ZN6\|CESA8_ORYSJ Probable cellulose synthase A catalytic subunit 8 [UDP- forming] OS=Oryza sativa subsp. japonica OX=39947 GN=CESA8 PE=1 SV=1 | map00500 |
| Dioal.09G032200.1 | EC:2.4.1.21 | OG0005148 | Genome Annotation | sp\|Q43846\|SSY3_SOLTU Soluble map00500 synthase 3, chloroplastic/amyloplastic OS=Solanum tuberosum OX=4113 GN=SS3 PE=1 SV=1 | map00500 |
| Dioal.09G085500.1 | EC:2.4.1.1 | OG0002392 | Genome Annotation | sp\|P53537\|PHSH_VICFA Alpha-glucan phosphorylase, H isozyme OS=Vicia faba  OX=3906 PE=2 SV=1 | map00500 |
| Dioal.10G014800.1 | EC:2.4.1.34 | OG0082990 | Genome Annotation | sp\|Q7XUK4\|GUN12_ORYSJ Endoglucanase 12 OS=Oryza sativa subsp. japonica  OX=39947 GN=GLU3 PE=2 SV=2 | map00500 |
| Dioal.10G054800.1 | EC:3.2.1.4 | OG0001721 | Genome Annotation | A0A8J5L566_ZINOF\|Endoglucanase | map00500 |
| Dioal.11G000200.1 | EC:3.6.1.21 | OG0006928 | Genome Annotation | sp\|Q9SZ63\|NUD14_ARATH Nudix hydrolase 14, chloroplastic OS=Arabidopsis thaliana OX=3702 GN=NUDT14 PE=1 SV=2 | map00500 |
| Dioal.11G003300.1 | EC:2.4.1.242 | OG0004548 | Genome Annotation | sp\|Q43784\|SSG1_MANES Granule-bound map00500 synthase 1, chloroplastic/amyloplastic OS=Manihot esculenta OX=3983 GN=WAXY PE=2 SV=1 | map00500 |
| Dioal.11G015700.1 | EC:3.2.1.4 | OG0009270 | Genome Annotation | sp\|Q69NF5\|GUN23_ORYSJ Endoglucanase 23 OS=Oryza sativa subsp. japonica  OX=39947 GN=GLU12 PE=2 SV=1 | map00500 |
| Dioal.11G019100.1 | EC:2.7.1.4 | OG0002795 | Genome Annotation | sp\|Q9FLH8\|SCRK7_ARATH Probable fructokinase-7 OS=Arabidopsis thaliana  OX=3702 GN=At5g51830 PE=1 SV=1 | map00500 |
| Dioal.11G027600.1 | EC:2.4.1.34 | OG0002788 | Genome Annotation | sp\|Q9SJM0\|CALSA_ARATH Callose synthase 10 OS=Arabidopsis thaliana  OX=3702 GN=CALS10 PE=2 SV=5 | map00500 |
| Dioal.11G039500.1 | EC:3.2.1.2 | OG0011059 | Genome Annotation | sp\|Q9FH80\|BAM8_ARATH Beta-amylase 8 OS=Arabidopsis thaliana OX=3702  GN=BAM8 PE=1 SV=1 | map00500 |
| Dioal.11G051100.1 | EC:2.7.1.1 | OG0000334 | Genome Annotation | sp\|Q6Z398\|HXK4_ORYSJ Hexokinase-4, chloroplastic OS=Oryza sativa subsp.  japonica OX=39947 GN=HXK4 PE=2 SV=1 | map00500 |
| Dioal.11G072300.1 | EC:2.4.1.34 | OG0000252 | Genome Annotation | sp\|Q9LXT9\|CALS3_ARATH Callose synthase 3 OS=Arabidopsis thaliana OX=3702  GN=CALS3 PE=3 SV=3 | map00500 |
| Dioal.11G072500.1 | EC:2.7.7.9 | OG0001502 | Genome Annotation | sp\|Q9SDX3\|UGPA_MUSAC UTP--glucose-1-phosphate uridylyltransferase  OS=Musa acuminata OX=4641 GN=UGPA PE=2 SV=1 | map00500 |
| Dioal.12G022300.1 | EC:3.2.1.2 | OG0006811 | Genome Annotation | sp\|Q8VYW2\|BAM9_ARATH Inactive beta-amylase 9 OS=Arabidopsis thaliana  OX=3702 GN=BAM9 PE=2 SV=1 | map00500 |

|  |  |  |  | sp\|F4HQA1\|PAF1_ARATH Protein PAF1 homolog OS=Arabidopsis thaliana |  |
| --- | --- | --- | --- | --- | --- |
| Dioal.12G034900.1 |  | OG0019473 | Orthogroups | OX=3702 GN=VIP2 PE=1 SV=1 | map00500 |
|  |  |  |  | sp\|Q2KNB4\|HXK3_ORYSJ Hexokinase-3 OS=Oryza sativa subsp. japonica |  |
| Dioal.12G044100.1 | EC:2.7.1.1 | OG0000334 | Genome Annotation | OX=39947 GN=HXK3 PE=2 SV=1 | map00500 |
|  |  |  |  | sp\|P0C1U4\|GUN9_ORYSJ Endoglucanase 9 OS=Oryza sativa subsp. japonica |  |
| Dioal.12G056900.1 | EC:3.2.1.4 | OG0001721 | Genome Annotation | OX=39947 GN=GLU1 PE=2 SV=1 | map00500 |
|  |  |  |  | sp\|O64890\|GUN13_ARATH Endoglucanase 13 OS=Arabidopsis thaliana OX=3702 |  |
| Dioal.12G069200.1 | EC:3.2.1.4 | OG0008229 | Genome Annotation | GN=At2g44550 PE=2 SV=1 | map00500 |
|  |  |  |  | sp\|O64890\|GUN13_ARATH Endoglucanase 13 OS=Arabidopsis thaliana OX=3702 |  |
| Dioal.12G069300.1 | EC:3.2.1.4 | OG0008229 | Genome Annotation | GN=At2g44550 PE=2 SV=1 | map00500 |
|  |  |  |  | sp\|Q9M394\|SCKL1_ARATH Fructokinase-like 1, chloroplastic OS=Arabidopsis |  |
| Dioal.12G094700.1 | EC:2.7.1.4 | OG0009109 | Genome Annotation | thaliana OX=3702 GN=FLN1 PE=1 SV=1 | map00500 |
|  |  |  |  | sp\|P27598\|PHSL_IPOBA Alpha-1,4 glucan phosphorylase L isozyme, |  |
| Dioal.13G021600.1 | EC:2.4.1.1 | OG0018484 | Genome Annotation | chloroplastic/amyloplastic OS=Ipomoea batatas OX=4120 PE=2 SV=1 | map00500 |
|  |  |  |  | sp\|P53536\|PHSL_VICFA Alpha-1,4 glucan phosphorylase L isozyme, |  |
| Dioal.13G021700.1 | EC:2.4.1.1 | OG0002392 | Genome Annotation | chloroplastic/amyloplastic OS=Vicia faba OX=3906 GN=PHO1 PE=2 SV=2 | map00500 |
|  |  |  |  | sp\|Q84R49\|GUN10_ORYSJ Endoglucanase 10 OS=Oryza sativa subsp. japonica |  |
| Dioal.13G082100.1 | EC:3.2.1.4 | OG0001721 | Genome Annotation | OX=39947 GN=GLU2 PE=2 SV=1 | map00500 |
|  |  |  |  | sp\|A5JTQ3\|XYL2_MEDSV Beta-xylosidase/alpha-L-arabinofuranosidase 2 |  |
| Dioal.13G093000.1 | EC:3.2.1.58 | OG0000457 | Genome Annotation | OS=Medicago sativa subsp. varia OX=36902 GN=Xyl2 PE=2 SV=1 | map00500 |
|  |  |  |  | sp\|A5JTQ3\|XYL2_MEDSV Beta-xylosidase/alpha-L-arabinofuranosidase 2 |  |
| Dioal.13G093100.1 | EC:3.2.1.58 | OG0012881 | Genome Annotation | OS=Medicago sativa subsp. varia OX=36902 GN=Xyl2 PE=2 SV=2 | map00500 |
|  |  |  |  | sp\|A5JTQ3\|XYL2_MEDSV Beta-xylosidase/alpha-L-arabinofuranosidase 2 |  |
| Dioal.13G093500.1 | EC:3.2.1.58 | OG0000457 | Genome Annotation | OS=Medicago sativa subsp. varia OX=36902 GN=Xyl2 PE=2 SV=1 | map00500 |
|  |  |  |  | sp\|A5JTQ3\|XYL2_MEDSV Beta-xylosidase/alpha-L-arabinofuranosidase 2 |  |
| Dioal.13G093600.1 | EC:3.2.1.58 | OG0000457 | Genome Annotation | OS=Medicago sativa subsp. varia OX=36902 GN=Xyl2 PE=2 SV=1 | map00500 |
|  |  |  |  | sp\|Q9LIR6\|BAM1_ARATH Beta-amylase 1, chloroplastic OS=Arabidopsis thaliana |  |
| Dioal.14G009900.1 | EC:3.2.1.2 | OG0002084 | Genome Annotation | OX=3702 GN=BAM1 PE=1 SV=1 | map00500 |
|  |  |  |  | sp\|A2XYW8\|GUN13_ORYSI Endoglucanase 13 OS=Oryza sativa subsp. indica |  |
| Dioal.14G031600.1 | EC:3.2.1.4 | OG0010860 | Genome Annotation | OX=39946 GN=GLU6 PE=3 SV=2 | map00500 |
|  |  |  |  | sp\|Q8LFG1\|AMY2_ARATH Probable alpha-amylase 2 OS=Arabidopsis thaliana |  |
| Dioal.14G047200.1 | EC:3.2.1.1 | OG0008881 | Genome Annotation | OX=3702 GN=AMY2 PE=2 SV=1 | map00500 |
|  |  |  |  | sp\|Q8LFG1\|AMY2_ARATH Probable alpha-amylase 2 OS=Arabidopsis thaliana |  |
| Dioal.14G047300.1 | EC:3.2.1.1 | OG0008881 | Genome Annotation | OX=3702 GN=AMY2 PE=2 SV=1 | map00500 |

Dioal.14G066900.1 EC:2.4.1.12 EC: OG0000179 Genome Annotation Dioal.14G081700.1 EC:2.7.1.4 OG0001115 Genome Annotation Dioal.14G127500.1 EC:2.4.1.34 OG0000252 Genome Annotation Dioal.14G143700.1 EC:3.2.1.4 OG0003884 Genome Annotation Dioal.15G006700.1 EC:2.7.1.4 OG0008521 Genome Annotation Dioal.15G034100.1 EC:2.7.1.1 OG0000334 Genome Annotation Dioal.15G046000.1 EC:2.7.1.1 OG0005155 Genome Annotation Dioal.15G075400.1 EC:3.2.1.58 OG0000457 Genome Annotation

sp|Q9SJ22|CESA9_ARATH Probable cellulose synthase A catalytic subunit 9 [UDP-

forming] OS=Arabidopsis thaliana OX=3702 GN=CESA9 PE=2 SV=1 map00500 sp|A2YQL4|SCRK2_ORYSI Fructokinase-2 OS=Oryza sativa subsp. indica

OX=39946 GN=FRK2 PE=1 SV=2 map00500

sp|Q9LXT9|CALS3_ARATH Callose synthase 3 OS=Arabidopsis thaliana OX=3702

GN=CALS3 PE=3 SV=3 map00500

sp|Q69SG5|GUN24_ORYSJ Endoglucanase 24 OS=Oryza sativa subsp. japonica

OX=39947 GN=Os09g0533900 PE=2 SV=1 map00500

sp|F4I0K2|SCKL2_ARATH Fructokinase-like 2, chloroplastic OS=Arabidopsis

thaliana OX=3702 GN=FLN2 PE=1 SV=2 map00500

sp|Q9SEK2|HXK1_TOBAC Hexokinase-1 OS=Nicotiana tabacum OX=4097

GN=HXK1 PE=2 SV=1 map00500

sp|Q9SEK2|HXK1_TOBAC Hexokinase-1 OS=Nicotiana tabacum OX=4097

GN=HXK1 PE=2 SV=2 map00500

sp|A5JTQ3|XYL2_MEDSV Beta-xylosidase/alpha-L-arabinofuranosidase 2

OS=Medicago sativa subsp. varia OX=36902 GN=Xyl2 PE=2 SV=1 map00500

Dioal.15G084200.1 OG0000179 Orthogroups Dioal.15G119500.1 EC:3.2.1.2 OG0004077 Genome Annotation Dioal.16G010200.1 EC:2.4.1.15 EC: OG0000282 Genome Annotation Dioal.16G034800.1 EC:3.1.3.12 OG0002034 Genome Annotation Dioal.16G046000.1 EC:5.4.2.2 EC:5. OG0010442 Genome Annotation Dioal.17G003400.1 OG0000179 Orthogroups

sp|Q84ZN6|CESA8_ORYSJ Probable cellulose synthase A catalytic subunit 8 [UDP-

forming] OS=Oryza sativa subsp. japonica OX=39947 GN=CESA8 PE=1 SV=1 map00500 sp|P10538|AMYB_SOYBN Beta-amylase OS=Glycine max OX=3847 GN=BMY1

PE=1 SV=3 map00500

sp|O23617|TPS5_ARATH Alpha,alpha-trehalose-phosphate synthase [UDP-

forming] 5 OS=Arabidopsis thaliana OX=3702 GN=TPS5 PE=1 SV=2 map00500 sp|Q9FWQ2|TPP2_ORYSJ Probable trehalose-phosphate phosphatase 2

OS=Oryza sativa subsp. japonica OX=39947 GN=TPP2 PE=1 SV=1 map00500 sp|Q9SM59|PGMP_PEA Phosphoglucomutase, chloroplastic OS=Pisum sativum

OX=3888 GN=PGMP PE=2 SV=1 map00500

sp|A2Y0X2|CESA1_ORYSI Probable cellulose synthase A catalytic subunit 1 [UDP-

forming] OS=Oryza sativa subsp. indica OX=39946 GN=CESA1 PE=3 SV=1 map00500

Dioal.17G025500.1 EC:3.6.1.9 OG0004307 Genome Annotation Uncharacterized protein map00500

sp|Q0DEC8|SSY1_ORYSJ Soluble map00500 synthase 1, chloroplastic/amyloplastic OS=Oryza sativa subsp. japonica OX=39947 GN=SS1

Dioal.17G051800.1 EC:2.4.1.21 OG0007735 Genome Annotation PE=1 SV=1 map00500

Dioal.17G059600.1 EC:3.2.1.2 OG0011526 Genome Annotation Dioal.17G063000.1 EC:2.4.1.34 OG0005465 Genome Annotation Dioal.17G063100.1 EC:2.4.1.34 OG0000252 Genome Annotation Dioal.17G069300.1 EC:3.2.1.4 OG0005413 Genome Annotation Dioal.17G084700.1 EC:5.4.2.2 EC:5. OG0011236 Genome Annotation Dioal.17G112400.1 EC:2.7.7.27 OG0000839 Genome Annotation Dioal.17G115600.1 OG0000179 Orthogroups Dioal.18G009100.1 EC:3.2.1.4 OG0008586 Genome Annotation Dioal.18G014200.1 EC:5.3.1.9 OG0007362 Genome Annotation Dioal.18G019500.1 EC:3.2.1.68 OG0008855 Genome Annotation Dioal.18G032200.1 EC:2.4.1.34 OG0020612 Genome Annotation Dioal.18G053800.1 EC:2.4.1.15 EC: OG0000282 Genome Annotation Dioal.18G063800.1 EC:3.1.3.12 OG0000588 Genome Annotation

Dioal.18G067300.1 EC:2.4.1.25 OG0009767 Genome Annotation

sp|O23553|BAM3_ARATH Beta-amylase 3, chloroplastic OS=Arabidopsis thaliana

OX=3702 GN=BAM3 PE=1 SV=3 map00500

sp|Q9LUD7|CALS8_ARATH Putative callose synthase 8 OS=Arabidopsis thaliana

OX=3702 GN=CALS8 PE=3 SV=2 map00500

sp|Q9LUD7|CALS8_ARATH Putative callose synthase 8 OS=Arabidopsis thaliana

OX=3702 GN=CALS8 PE=3 SV=2 map00500

sp|Q42059|GUN6_ARATH Endoglucanase 6 OS=Arabidopsis thaliana OX=3702 GN=At1g64390 PE=2 SV=2 map00500

A0A6I9QXC6_ELAGV|phosphoglucomutase (alpha-D-glucose-1,6-bisphosphate-

dependent) map00500

sp|P55230|GLGL2_ARATH Glucose-1-phosphate adenylyltransferase large

subunit 2, chloroplastic OS=Arabidopsis thaliana OX=3702 GN=APL2 PE=1 SV=2 map00500 sp|Q941L0|CESA3_ARATH Cellulose synthase A catalytic subunit 3 [UDP-forming] OS=Arabidopsis thaliana OX=3702 GN=CESA3 PE=1 SV=2 map00500 sp|Q6K7G9|GUN8_ORYSJ Endoglucanase 8 OS=Oryza sativa subsp. japonica

OX=39947 GN=Os02g0778600 PE=2 SV=1 map00500

sp|Q8H103|G6PIP_ARATH Glucose-6-phosphate isomerase 1, chloroplastic

OS=Arabidopsis thaliana OX=3702 GN=PGI1 PE=1 SV=1 map00500 sp|B9G434|ISOA3_ORYSJ Isoamylase 3, chloroplastic OS=Oryza sativa subsp.

japonica OX=39947 GN=ISA3 PE=2 SV=1 map00500

sp|Q9SJM0|CALSA_ARATH Callose synthase 10 OS=Arabidopsis thaliana

OX=3702 GN=CALS10 PE=2 SV=5 map00500

sp|Q94AH8|TPS6_ARATH Alpha,alpha-trehalose-phosphate synthase [UDP-

forming] 6 OS=Arabidopsis thaliana OX=3702 GN=TPS6 PE=1 SV=2 map00500 sp|Q6ZAL2|TPP6_ORYSJ Probable trehalose-phosphate phosphatase 6 OS=Oryza

sativa subsp. japonica OX=39947 GN=TPP6 PE=2 SV=1 map00500 sp|Q8LI30|DPE1_ORYSJ 4-alpha-glucanotransferase DPE1,

chloroplastic/amyloplastic OS=Oryza sativa subsp. japonica OX=39947 GN=DPE1

PE=2 SV=2 map00500

Dioal.18G070300.1 EC:2.4.1.21 OG0004237 Genome Annotation Dioal.18G093900.1 EC:2.7.7.9 OG0009535 Genome Annotation

sp|Q43847|SSY2_SOLTU Granule-bound map00500 synthase 2,

chloroplastic/amyloplastic OS=Solanum tuberosum OX=4113 GN=SS2 PE=1 SV=3 map00500 sp|F4IY62|UGPA3_ARATH UTP--glucose-1-phosphate uridylyltransferase 3,

chloroplastic OS=Arabidopsis thaliana OX=3702 GN=UGP3 PE=1 SV=1 map00500

Dioal.18G106400.1 EC:3.2.1.4 OG0046014 Genome Annotation Uncharacterized protein map00500

sp|A5JTQ3|XYL2_MEDSV Beta-xylosidase/alpha-L-arabinofuranosidase 2

Dioal.18G118300.1 EC:3.2.1.58 OG0005039 Genome Annotation OS=Medicago sativa subsp. varia OX=36902 GN=Xyl2 PE=2 SV=1 map00500

Dioal.18G119600.1 EC:3.2.1.1 OG0004196 Genome Annotation A0A8K0ITS2_COCNU|CBM20 domain-containing protein map00500

sp|Q6ZAL2|TPP6_ORYSJ Probable trehalose-phosphate phosphatase 6 OS=Oryza

Dioal.19G036500.1 EC:3.1.3.12 OG0000588 Genome Annotation Dioal.19G065400.1 EC:3.2.1.58 OG0000457 Genome Annotation Dioal.19G065500.1 EC:3.2.1.58 OG0000457 Genome Annotation Dioal.19G091000.1 EC:2.4.1.34 OG0002788 Genome Annotation Dioal.19G149200.1 EC:2.7.7.23 EC: OG0005426 Genome Annotation Dioal.19G165600.1 EC:2.7.7.27 OG0000839 Genome Annotation

Dioal.20G036800.1 EC:2.7.7.27 OG0000839 Genome Annotation

sativa subsp. japonica OX=39947 GN=TPP6 PE=2 SV=1 map00500 sp|A5JTQ3|XYL2_MEDSV Beta-xylosidase/alpha-L-arabinofuranosidase 2

OS=Medicago sativa subsp. varia OX=36902 GN=Xyl2 PE=2 SV=1 map00500 sp|Q9FGY1|BXL1_ARATH Beta-D-xylosidase 1 OS=Arabidopsis thaliana OX=3702

GN=BXL1 PE=1 SV=1 map00500

sp|Q9SFU6|CALS9_ARATH Callose synthase 9 OS=Arabidopsis thaliana OX=3702

GN=CALS9 PE=2 SV=2 map00500

sp|O64765|UAP2_ARATH UDP-N-acetylglucosamine diphosphorylase 2

OS=Arabidopsis thaliana OX=3702 GN=GLCNAC1PUT2 PE=1 SV=1 map00500 sp|P55229|GLGL1_ARATH Glucose-1-phosphate adenylyltransferase large

subunit 1, chloroplastic OS=Arabidopsis thaliana OX=3702 GN=ADG2 PE=1 SV=3 map00500 sp|P55233|GLGL1_BETVU Glucose-1-phosphate adenylyltransferase large

subunit, chloroplastic/amyloplastic OS=Beta vulgaris OX=161934 GN=AGPS1 PE=2

SV=1 map00500

Dioal.20G037500.1 EC:2.4.1.21 OG0009507 Genome Annotation Dioal.20G041900.1 EC:2.7.1.1 OG0000334 Genome Annotation Dioal.20G057000.1 EC:2.4.1.15 EC: OG0000282 Genome Annotation Dioal.20G083600.1 EC:2.4.1.34 OG0003279 Genome Annotation Dioal.20G083900.1 EC:2.4.1.1 OG0013282 Genome Annotation Dioal.20G091400.1 EC:3.6.1.9 OG0004307 Genome Annotation

sp|Q0WVX5|SSY4_ARATH Probable map00500 synthase 4,

chloroplastic/amyloplastic OS=Arabidopsis thaliana OX=3702 GN=SS4 PE=1 SV=1 map00500 sp|P93834|HXK2_ARATH Hexokinase-2 OS=Arabidopsis thaliana OX=3702

GN=HXK2 PE=1 SV=1 map00500

sp|Q9LMI0|TPS7_ARATH Probable alpha,alpha-trehalose-phosphate synthase

[UDP-forming] 7 OS=Arabidopsis thaliana OX=3702 GN=TPS7 PE=1 SV=1 map00500 sp|Q9ZT82|CALSC_ARATH Callose synthase 12 OS=Arabidopsis thaliana

OX=3702 GN=CALS12 PE=2 SV=1 map00500

sp|Q9SD76|PHS2_ARATH Alpha-glucan phosphorylase 2, cytosolic

OS=Arabidopsis thaliana OX=3702 GN=PHS2 PE=1 SV=1 map00500 A0A6P4ASK0_ZIZJJ|ectonucleotide pyrophosphatase/phosphodiesterase family

member 1 map00500

|  | | | | sp\|Q6YYX9\|SLC1_ORYSJ Probable 2-oxoglutarate-dependent dioxygenase SLC1 |  |
| --- | --- | --- | --- | --- | --- |
| Dioal.01G016100.1 | EC:1.14.11.9 | OG0004472 | Genome Annotation | OS=Oryza sativa subsp. japonica OX=39947 GN=SLC1 PE=2 SV=1 | map00941 |
|  |  |  |  | sp\|Q6YYX9\|SLC1_ORYSJ Probable 2-oxoglutarate-dependent dioxygenase SLC1 |  |
| Dioal.01G016200.1 | EC:1.14.11.9 | OG0004472 |  | OS=Oryza sativa subsp. japonica OX=39947 GN=SLC1 PE=2 SV=1 | map00941 |
|  |  |  |  | sp\|Q9AT54\|SCGT_TOBAC Scopoletin glucosyltransferase OS=Nicotiana tabacum |  |
| Dioal.02G010500.1 |  | OG0000290 | Orthogroups | OX=4097 GN=TOGT1 PE=1 SV=1 | map00941 |
|  |  |  |  | sp\|Q2V6J9\|UFOG7_FRAAN UDP-glucose flavonoid 3-O-glucosyltransferase 7 |  |
| Dioal.02G010900.1 |  | OG0000290 | Orthogroups | OS=Fragaria ananassa OX=3747 GN=GT7 PE=1 SV=1 | map00941 |
|  |  |  |  | sp\|Q9AT54\|SCGT_TOBAC Scopoletin glucosyltransferase OS=Nicotiana tabacum |  |
| Dioal.02G011000.1 | EC:2.4.1.185 | OG0000290 |  | OX=4097 GN=TOGT1 PE=1 SV=1 | map00941 |
|  |  |  |  | sp\|O82054\|COMT1_SACOF Caffeic acid 3-O-methyltransferase OS=Saccharum |  |
| Dioal.02G026600.1 |  | OG0000213 | Orthogroups | officinarum OX=4547 GN=COMT PE=2 SV=1 | map00941 |
|  |  |  |  | sp\|Q8W013\|COMT1_CATRO Caffeic acid 3-O-methyltransferase |  |
| Dioal.02G073700.1 |  | OG0000213 | Orthogroups | OS=Catharanthus roseus OX=4058 GN=COMT1 PE=2 SV=1 | map00941 |
|  |  |  |  | sp\|Q94C57\|U73B2_ARATH UDP-glucosyl transferase 73B2 OS=Arabidopsis |  |
| Dioal.03G018700.1 |  | OG0001609 | Orthogroups | thaliana OX=3702 GN=UGT73B2 PE=1 SV=1 | map00941 |
|  |  |  |  | sp\|Q9AT54\|SCGT_TOBAC Scopoletin glucosyltransferase OS=Nicotiana tabacum |  |
| Dioal.03G018800.1 |  | OG0001609 | Orthogroups | OX=4097 GN=TOGT1 PE=1 SV=1 | map00941 |
|  |  |  |  | sp\|Q9AT54\|SCGT_TOBAC Scopoletin glucosyltransferase OS=Nicotiana tabacum |  |
| Dioal.03G018900.1 |  | OG0001609 | Orthogroups | OX=4097 GN=TOGT1 PE=1 SV=1 | map00941 |
|  |  |  |  | sp\|Q9AT54\|SCGT_TOBAC Scopoletin glucosyltransferase OS=Nicotiana tabacum |  |
| Dioal.03G019000.1 |  | OG0001609 | Orthogroups | OX=4097 GN=TOGT1 PE=1 SV=1 | map00941 |
|  |  |  |  | sp\|Q8GSM7\|HST_TOBAC Shikimate O-hydroxycinnamoyltransferase |  |
| Dioal.03G024200.1 |  | OG0000415 | Orthogroups | OS=Nicotiana tabacum OX=4097 GN=HST PE=1 SV=1 | map00941 |
|  |  |  |  | sp\|Q9FI78\|HST_ARATH Shikimate O-hydroxycinnamoyltransferase |  |
| Dioal.03G031200.1 |  | OG0000415 | Orthogroups | OS=Arabidopsis thaliana OX=3702 GN=HST PE=1 SV=1 | map00941 |
|  |  |  |  | sp\|Q8GSM7\|HST_TOBAC Shikimate O-hydroxycinnamoyltransferase |  |
| Dioal.03G031300.1 |  | OG0000415 | Orthogroups | OS=Nicotiana tabacum OX=4097 GN=HST PE=1 SV=1 | map00941 |
|  |  |  |  | sp\|Q9SND9\|Y3028_ARATH Uncharacterized acetyltransferase At3g50280 |  |
| Dioal.03G032100.1 |  | OG0000207 | Orthogroups | OS=Arabidopsis thaliana OX=3702 GN=At3g50280 PE=3 SV=1 | map00941 |
|  |  |  |  | sp\|O80449\|DIOX4_ARATH Probable 2-oxoglutarate-dependent dioxygenase ANS |  |
| Dioal.03G035900.1 |  | OG0000510 | Orthogroups | OS=Arabidopsis thaliana OX=3702 GN=ANS PE=2 SV=1 | map00941 |
|  |  |  |  | sp\|O80449\|DIOX4_ARATH Probable 2-oxoglutarate-dependent dioxygenase ANS |  |
| Dioal.03G036000.1 |  | OG0000510 | Orthogroups | OS=Arabidopsis thaliana OX=3702 GN=ANS PE=2 SV=1 | map00941 |

Dioal.03G036100.1 OG0000510 Orthogroups Dioal.03G036200.1 EC:1.14.11.23 E OG0000510 Genome Annotation Dioal.03G036300.1 OG0000510 Orthogroups

Dioal.03G036400.1 OG0000510 Orthogroups Dioal.03G042900.1 EC:2.3.1.133 OG0000207 Genome Annotation Dioal.03G043300.1 EC:2.3.1.133 OG0000207 Genome Annotation Dioal.03G043400.1 OG0000207 Orthogroups

Dioal.03G043600.1 OG0000207 Orthogroups

Dioal.04G073400.1 OG0000290 Orthogroups Dioal.04G075100.1 EC:1.1.1.219 OG0003437 Genome Annotation Dioal.04G171800.1 EC:2.3.1.133 EC OG0005029 Genome Annotation Dioal.04G172600.1 EC:1.14.11.9 OG0003307 Genome Annotation Dioal.05G128000.1 EC:2.4.1.185 OG0001609 Genome Annotation Dioal.05G139300.1 EC:2.3.1.74 OG0000150 Genome Annotation Dioal.05G141100.1 OG0000290 Orthogroups

Dioal.05G149100.1 OG0004604 Orthogroups Dioal.05G163700.1 EC:2.1.1.104 OG0000396 Genome Annotation

sp|O80449|DIOX4_ARATH Probable 2-oxoglutarate-dependent dioxygenase ANS OS=Arabidopsis thaliana OX=3702 GN=ANS PE=2 SV=1 map00941 sp|O80449|DIOX4_ARATH Probable 2-oxoglutarate-dependent dioxygenase ANS OS=Arabidopsis thaliana OX=3702 GN=ANS PE=2 SV=1 map00941 sp|O80449|DIOX4_ARATH Probable 2-oxoglutarate-dependent dioxygenase ANS OS=Arabidopsis thaliana OX=3702 GN=ANS PE=2 SV=1 map00941 sp|Q39224|SRG1_ARATH Protein SRG1 OS=Arabidopsis thaliana OX=3702

GN=SRG1 PE=2 SV=1 map00941

sp|Q9FH97|EPS1_ARATH Protein ENHANCED PSEUDOMONAS SUSCEPTIBILITY 1

OS=Arabidopsis thaliana OX=3702 GN=EPS1 PE=2 SV=1 map00941 sp|Q9SND9|Y3028_ARATH Uncharacterized acetyltransferase At3g50280

OS=Arabidopsis thaliana OX=3702 GN=At3g50280 PE=3 SV=1 map00941 sp|Q9SND9|Y3028_ARATH Uncharacterized acetyltransferase At3g50280

OS=Arabidopsis thaliana OX=3702 GN=At3g50280 PE=3 SV=1 map00941 sp|Q9SND9|Y3028_ARATH Uncharacterized acetyltransferase At3g50280

OS=Arabidopsis thaliana OX=3702 GN=At3g50280 PE=3 SV=1 map00941 sp|Q9AT54|SCGT_TOBAC Scopoletin glucosyltransferase OS=Nicotiana tabacum

OX=4097 GN=TOGT1 PE=1 SV=1 map00941

sp|P51110|DFRA_VITVI Dihydroflavonol 4-reductase OS=Vitis vinifera OX=29760

GN=DFR PE=1 SV=1 map00941

sp|Q9LIS1|CR26L_ARATH Protein ECERIFERUM 26-like OS=Arabidopsis thaliana

OX=3702 GN=CER26L PE=2 SV=1 map00941

sp|Q7XR84|FL3H3_ORYSJ Flavanone 3-dioxygenase 3 OS=Oryza sativa subsp.

japonica OX=39947 GN=F3H-3 PE=1 SV=1 map00941

sp|Q9ZVX4|U90A1_ARATH UDP-glycosyltransferase 90A1 OS=Arabidopsis

thaliana OX=3702 GN=UGT90A1 PE=2 SV=1 map00941

sp|P48387|CHS2_CAMSI Chalcone synthase 2 OS=Camellia sinensis OX=4442

GN=CHS2 PE=2 SV=1 map00941

sp|Q9AT54|SCGT_TOBAC Scopoletin glucosyltransferase OS=Nicotiana tabacum

OX=4097 GN=TOGT1 PE=1 SV=1 map00941

sp|Q9M1X2|FAP1_ARATH Fatty-acid-binding protein 1 OS=Arabidopsis thaliana

OX=3702 GN=FAP1 PE=1 SV=1 map00941

sp|A0A2H5AIZ6|NOMT_NARPS Norbelladine 4'-O-methyltransferase

OS=Narcissus pseudonarcissus OX=39639 GN=N4OMT PE=2 SV=1 map00941

|  | | | | sp\|A0A2H5AIZ6\|NOMT_NARPS Norbelladine 4'-O-methyltransferase |  |
| --- | --- | --- | --- | --- | --- |
| Dioal.05G163800.1 | EC:2.1.1.104 | OG0000396 | Genome Annotation | OS=Narcissus pseudonarcissus OX=39639 GN=N4OMT PE=2 SV=1 | map00941 |
|  |  |  |  | sp\|A0A2H5AIZ6\|NOMT_NARPS Norbelladine 4'-O-methyltransferase |  |
| Dioal.05G163900.1 | EC:2.1.1.104 | OG0000396 | Genome Annotation | OS=Narcissus pseudonarcissus OX=39639 GN=N4OMT PE=2 SV=1 | map00941 |
|  |  |  |  | sp\|A0A077EWA5\|NOMT_NARAP Norbelladine 4'-O-methyltransferase |  |
|  |  |  |  | OS=Narcissus aff. pseudonarcissus MK-2014 OX=1540222 GN=N4OMT PE=1 |  |
| Dioal.05G164000.1 | EC:2.1.1.104 | OG0000396 | Genome Annotation | SV=1 | map00941 |
|  |  |  |  | sp\|A0A077EWA5\|NOMT_NARAP Norbelladine 4'-O-methyltransferase |  |
|  |  |  |  | OS=Narcissus aff. pseudonarcissus MK-2014 OX=1540222 GN=N4OMT PE=1 |  |
| Dioal.05G164100.1 | EC:2.1.1.104 | OG0000396 | Genome Annotation | SV=1 | map00941 |
|  |  |  |  | sp\|Q9FFF6\|DIOX5_ARATH Probable 2-oxoglutarate-dependent dioxygenase |  |
| Dioal.07G112500.1 | EC:1.14.11.9 | OG0000931 | Genome Annotation | At5g05600 OS=Arabidopsis thaliana OX=3702 GN=At5g05600 PE=2 SV=1 | map00941 |
|  |  |  |  | sp\|Q5SMM8\|PHT1_ORYSJ Putrescine hydroxycinnamoyltransferase 1 OS=Oryza |  |
| Dioal.08G035700.1 |  | OG0000415 | Orthogroups | sativa subsp. japonica OX=39947 GN=PHT1 PE=2 SV=1 | map00941 |
|  |  |  |  | sp\|Q8GSM7\|HST_TOBAC Shikimate O-hydroxycinnamoyltransferase |  |
| Dioal.08G040100.1 | EC:2.3.1.133 | OG0000415 | Genome Annotation | OS=Nicotiana tabacum OX=4097 GN=HST PE=1 SV=1 | map00941 |
|  |  |  |  | sp\|Q43095\|CAMT_POPTM Caffeoyl-CoA O-methyltransferase OS=Populus |  |
| Dioal.08G085300.1 | EC:2.1.1.104 | OG0000396 | Genome Annotation | tremuloides OX=3693 PE=2 SV=1 | map00941 |
|  |  |  |  | sp\|Q5SMM8\|PHT1_ORYSJ Putrescine hydroxycinnamoyltransferase 1 OS=Oryza |  |
| Dioal.08G106200.1 | EC:2.3.1.133 | OG0000415 | Genome Annotation | sativa subsp. japonica OX=39947 GN=PHT1 PE=2 SV=1 | map00941 |
|  |  |  |  | sp\|Q5SMM8\|PHT1_ORYSJ Putrescine hydroxycinnamoyltransferase 1 OS=Oryza |  |
| Dioal.08G106400.1 |  | OG0000415 | Orthogroups | sativa subsp. japonica OX=39947 GN=PHT1 PE=2 SV=1 | map00941 |
|  |  |  |  | sp\|Q5SMM8\|PHT1_ORYSJ Putrescine hydroxycinnamoyltransferase 1 OS=Oryza |  |
| Dioal.08G106500.1 | EC:2.3.1.133 | OG0000415 | Genome Annotation | sativa subsp. japonica OX=39947 GN=PHT1 PE=2 SV=1 | map00941 |
|  |  |  |  | sp\|Q5SMM8\|PHT1_ORYSJ Putrescine hydroxycinnamoyltransferase 1 OS=Oryza |  |
| Dioal.08G106600.1 | EC:2.3.1.133 | OG0000415 | Genome Annotation | sativa subsp. japonica OX=39947 GN=PHT1 PE=2 SV=1 | map00941 |
|  |  |  |  | sp\|Q5SMM8\|PHT1_ORYSJ Putrescine hydroxycinnamoyltransferase 1 OS=Oryza |  |
| Dioal.08G106800.1 |  | OG0000415 | Orthogroups | sativa subsp. japonica OX=39947 GN=PHT1 PE=2 SV=1 | map00941 |
|  |  |  |  | sp\|Q5SMM8\|PHT1_ORYSJ Putrescine hydroxycinnamoyltransferase 1 OS=Oryza |  |
| Dioal.08G106900.1 |  | OG0000415 | Orthogroups | sativa subsp. japonica OX=39947 GN=PHT1 PE=2 SV=1 | map00941 |
|  |  |  |  | sp\|A0A2H5AIZ1\|HCT_NARPS Hydroxycinnamoyltransferase OS=Narcissus |  |
| Dioal.08G107000.1 | EC:2.3.1.133 | OG0000415 | Genome Annotation | pseudonarcissus OX=39639 GN=HCT PE=2 SV=1 | map00941 |
|  |  |  |  | sp\|A0A2H5AIZ1\|HCT_NARPS Hydroxycinnamoyltransferase OS=Narcissus |  |
| Dioal.08G107100.1 | EC:2.3.1.133 | OG0000415 | Genome Annotation | pseudonarcissus OX=39639 GN=HCT PE=2 SV=1 | map00941 |

Dioal.08G131200.1 EC:5.5.1.6 OG0004604 Genome Annotation Dioal.09G035500.1 EC:1.1.1.219 OG0008522 Genome Annotation Dioal.09G035600.1 EC:1.1.1.219 OG0008522 Genome Annotation Dioal.09G071500.1 EC:5.5.1.6 OG0004708 Genome Annotation Dioal.10G004500.1 EC:2.3.1.74 OG0000150 Genome Annotation

Dioal.10G018400.1 EC:2.1.1.104 OG0000396 Genome Annotation

Dioal.10G018600.1 EC:2.1.1.104 OG0000396 Genome Annotation Dioal.10G018700.1 EC:2.1.1.104 OG0000396 Genome Annotation

Dioal.10G018800.1 EC:2.1.1.104 OG0000396 Genome Annotation Dioal.10G037900.1 OG0004604 Orthogroups Dioal.11G005600.1 EC:2.1.1.104 OG0009244 Genome Annotation Dioal.11G008200.1 OG0000213 Orthogroups Dioal.11G008300.1 EC:2.1.1.104 EC OG0000213 Genome Annotation Dioal.11G036600.1 EC:2.1.1.104 EC OG0000213 Genome Annotation Dioal.11G053100.1 EC:1.14.11.9 OG0004599 Genome Annotation

sp|Q9M1X2|FAP1_ARATH Fatty-acid-binding protein 1 OS=Arabidopsis thaliana

OX=3702 GN=FAP1 PE=1 SV=1 map00941

sp|P51110|DFRA_VITVI Dihydroflavonol 4-reductase OS=Vitis vinifera OX=29760

GN=DFR PE=1 SV=1 map00941

sp|Q5XLY0|ANR_GINBI Putative anthocyanidin reductase OS=Ginkgo biloba

OX=3311 PE=2 SV=1 map00941

sp|Q45QI7|CFI_CAMSI Chalcone--flavonone isomerase OS=Camellia sinensis

OX=4442 GN=CHI PE=2 SV=2 map00941

sp|P51090|CHSY_VITVI Chalcone synthase OS=Vitis vinifera OX=29760 GN=CHS

PE=2 SV=1 map00941

sp|A0A077EWA5|NOMT_NARAP Norbelladine 4'-O-methyltransferase OS=Narcissus aff. pseudonarcissus MK-2014 OX=1540222 GN=N4OMT PE=1

SV=1 map00941

sp|A0A077EW86|NOMT2_NARAP Norbelladine 4'-O-methyltransferase 2 OS=Narcissus aff. pseudonarcissus MK-2014 OX=1540222 GN=N4OMT2 PE=2

SV=1 map00941

sp|A0A2H5AIZ6|NOMT_NARPS Norbelladine 4'-O-methyltransferase

OS=Narcissus pseudonarcissus OX=39639 GN=N4OMT PE=2 SV=1 map00941 sp|A0A077EWA5|NOMT_NARAP Norbelladine 4'-O-methyltransferase

OS=Narcissus aff. pseudonarcissus MK-2014 OX=1540222 GN=N4OMT PE=1

SV=1 map00941

sp|Q9M1X2|FAP1_ARATH Fatty-acid-binding protein 1 OS=Arabidopsis thaliana

OX=3702 GN=FAP1 PE=1 SV=1 map00941

sp|Q9XGP7|OMT15_ORYSJ Tricin synthase 1 OS=Oryza sativa subsp. japonica

OX=39947 GN=ROMT-15 PE=1 SV=1 map00941

sp|Q9FK25|OMT1_ARATH Flavone 3'-O-methyltransferase 1 OS=Arabidopsis

thaliana OX=3702 GN=OMT1 PE=1 SV=1 map00941

sp|Q38J50|FOMT2_WHEAT Tricetin 3',4',5'-O-trimethyltransferase OS=Triticum

aestivum OX=4565 GN=OMT2 PE=1 SV=1 map00941

sp|Q38J50|FOMT2_WHEAT Tricetin 3',4',5'-O-trimethyltransferase OS=Triticum

aestivum OX=4565 GN=OMT2 PE=1 SV=1 map00941

sp|A0A4D6Q4T7|FL3H2_CROXC Flavanone 3-dioxygenase F3H2 OS=Crocosmia x crocosmiiflora OX=1053288 GN=F3H-2 PE=1 SV=1 map00941

|  | | | | sp\|Q6YYX9\|SLC1_ORYSJ Probable 2-oxoglutarate-dependent dioxygenase SLC1 |  |
| --- | --- | --- | --- | --- | --- |
| Dioal.11G084800.1 | EC:1.14.11.9 | OG0004472 | Genome Annotation | OS=Oryza sativa subsp. japonica OX=39947 GN=SLC1 PE=2 SV=1 | map00941 |
|  |  |  |  | sp\|Q40062\|IDS3_HORVU 2'-deoxymugineic-acid 2'-dioxygenase OS=Hordeum |  |
| Dioal.11G089300.1 |  | OG0000313 | Orthogroups | vulgare OX=4513 GN=IDS3 PE=1 SV=3 | map00941 |
|  |  |  |  | sp\|Q9FFF6\|DIOX5_ARATH Probable 2-oxoglutarate-dependent dioxygenase |  |
| Dioal.12G070300.1 |  | OG0000931 | Orthogroups | At5g05600 OS=Arabidopsis thaliana OX=3702 GN=At5g05600 PE=2 SV=1 | map00941 |
|  |  |  |  | sp\|Q9ZRR8\|CHS1_CASGL Chalcone synthase OS=Casuarina glauca OX=3522 |  |
| Dioal.13G078400.1 | EC:2.3.1.74 | OG0000150 | Genome Annotation | GN=CHS1 PE=2 SV=1 | map00941 |
|  |  |  |  | sp\|Q00763\|COMT1_POPTM Caffeic acid 3-O-methyltransferase 1 OS=Populus |  |
| Dioal.14G034200.1 |  | OG0000213 | Orthogroups | tremuloides OX=3693 GN=OMT1 PE=1 SV=1 | map00941 |
|  |  |  |  | sp\|Q9AT54\|SCGT_TOBAC Scopoletin glucosyltransferase OS=Nicotiana tabacum |  |
| Dioal.14G059300.1 |  | OG0000290 | Orthogroups | OX=4097 GN=TOGT1 PE=1 SV=1 | map00941 |
|  |  |  |  | sp\|Q9AT54\|SCGT_TOBAC Scopoletin glucosyltransferase OS=Nicotiana tabacum |  |
| Dioal.14G059400.1 |  | OG0000290 | Orthogroups | OX=4097 GN=TOGT1 PE=1 SV=1 | map00941 |
|  |  |  |  | sp\|Q8W2X5\|FL3H2_ORYSJ Flavanone 3-dioxygenase 2 OS=Oryza sativa subsp. |  |
| Dioal.14G121900.1 | EC:1.14.11.9 | OG0000313 | Genome Annotation | japonica OX=39947 GN=F3H-2 PE=1 SV=1 | map00941 |
|  |  |  |  | sp\|Q9C899\|F6H2_ARATH Feruloyl CoA ortho-hydroxylase 2 OS=Arabidopsis |  |
| Dioal.15G024300.1 |  | OG0001137 | Orthogroups | thaliana OX=3702 GN=F6'H2 PE=1 SV=1 | map00941 |
|  |  |  |  | sp\|Q9FFF6\|DIOX5_ARATH Probable 2-oxoglutarate-dependent dioxygenase |  |
| Dioal.15G024400.1 |  | OG0001137 | Orthogroups | At5g05600 OS=Arabidopsis thaliana OX=3702 GN=At5g05600 PE=2 SV=1 | map00941 |
|  |  |  |  | sp\|Q9FFF6\|DIOX5_ARATH Probable 2-oxoglutarate-dependent dioxygenase |  |
| Dioal.15G024500.1 |  | OG0001137 | Orthogroups | At5g05600 OS=Arabidopsis thaliana OX=3702 GN=At5g05600 PE=2 SV=1 | map00941 |
|  |  |  |  | sp\|P41090\|FL3H_VITVI Naringenin,2-oxoglutarate 3-dioxygenase OS=Vitis |  |
| Dioal.15G024600.1 | EC:1.14.11.9 | OG0001137 | Genome Annotation | vinifera OX=29760 GN=F3H PE=2 SV=1 | map00941 |
|  |  |  |  | sp\|Q9C899\|F6H2_ARATH Feruloyl CoA ortho-hydroxylase 2 OS=Arabidopsis |  |
| Dioal.15G024700.1 |  | OG0001137 | Orthogroups | thaliana OX=3702 GN=F6'H2 PE=1 SV=1 | map00941 |
|  |  |  |  | sp\|Q9ZSA8\|DLO1_ARATH Protein DMR6-LIKE OXYGENASE 1 OS=Arabidopsis |  |
| Dioal.15G024800.1 |  | OG0001137 | Orthogroups | thaliana OX=3702 GN=DLO1 PE=1 SV=1 | map00941 |
|  |  |  |  | sp\|Q9FFF6\|DIOX5_ARATH Probable 2-oxoglutarate-dependent dioxygenase |  |
| Dioal.15G024900.1 |  | OG0001137 | Orthogroups | At5g05600 OS=Arabidopsis thaliana OX=3702 GN=At5g05600 PE=2 SV=1 | map00941 |
|  |  |  |  | sp\|Q9FFF6\|DIOX5_ARATH Probable 2-oxoglutarate-dependent dioxygenase |  |
| Dioal.15G025000.1 | EC:1.14.11.9 | OG0001137 | Genome Annotation | At5g05600 OS=Arabidopsis thaliana OX=3702 GN=At5g05600 PE=2 SV=1 | map00941 |
|  |  |  |  | sp\|D4N502\|DIOX3_PAPSO Codeine O-demethylase OS=Papaver somniferum |  |
| Dioal.15G025100.1 |  | OG0001137 | Orthogroups | OX=3469 GN=CODM PE=1 SV=1 | map00941 |

|  |  |  |  | sp\|Q9SND9\|Y3028_ARATH Uncharacterized acetyltransferase At3g50280 |  |
| --- | --- | --- | --- | --- | --- |
| Dioal.17G061600.1 | EC:2.3.1.133 | OG0000207 | Genome Annotation | OS=Arabidopsis thaliana OX=3702 GN=At3g50280 PE=3 SV=1 | map00941 |
|  |  |  |  | sp\|Q9SND9\|Y3028_ARATH Uncharacterized acetyltransferase At3g50280 |  |
| Dioal.17G061800.1 | EC:2.3.1.133 | OG0000207 | Genome Annotation | OS=Arabidopsis thaliana OX=3702 GN=At3g50280 PE=3 SV=1 | map00941 |
|  |  |  |  | sp\|Q9SND9\|Y3028_ARATH Uncharacterized acetyltransferase At3g50280 |  |
| Dioal.17G061900.1 | EC:2.3.1.133 | OG0000207 | Genome Annotation | OS=Arabidopsis thaliana OX=3702 GN=At3g50280 PE=3 SV=1 | map00941 |
|  |  |  |  | sp\|Q9FH97\|EPS1_ARATH Protein ENHANCED PSEUDOMONAS SUSCEPTIBILITY 1 |  |
| Dioal.17G062100.1 | EC:2.3.1.133 | OG0000207 | Genome Annotation | OS=Arabidopsis thaliana OX=3702 GN=EPS1 PE=2 SV=1 | map00941 |
|  |  |  |  | sp\|Q5XLY0\|ANR_GINBI Putative anthocyanidin reductase OS=Ginkgo biloba |  |
| Dioal.17G071000.1 | EC:1.3.1.77 | OG0002952 | Genome Annotation | OX=3311 PE=2 SV=1 | map00941 |
|  |  |  |  | sp\|Q5XLY0\|ANR_GINBI Putative anthocyanidin reductase OS=Ginkgo biloba |  |
| Dioal.17G071100.1 | EC:1.3.1.77 | OG0002952 | Genome Annotation | OX=3311 PE=2 SV=1 | map00941 |
|  |  |  |  | sp\|Q9C8L2\|FAP3_ARATH Fatty-acid-binding protein 3, chloroplastic |  |
| Dioal.17G082800.1 | EC:5.5.1.6 | OG0010193 | Genome Annotation | OS=Arabidopsis thaliana OX=3702 GN=FAP3 PE=1 SV=1 | map00941 |
|  |  |  |  | sp\|P22928\|CHSJ_PETHY Chalcone synthase J OS=Petunia hybrida OX=4102 |  |
| Dioal.17G104600.1 | EC:2.3.1.74 | OG0000150 | Genome Annotation | GN=CHSJ PE=2 SV=2 | map00941 |
|  |  |  |  | sp\|P22928\|CHSJ_PETHY Chalcone synthase J OS=Petunia hybrida OX=4102 |  |
| Dioal.17G104700.1 | EC:2.3.1.74 | OG0000150 | Genome Annotation | GN=CHSJ PE=2 SV=2 | map00941 |
|  |  |  |  | sp\|P22928\|CHSJ_PETHY Chalcone synthase J OS=Petunia hybrida OX=4102 |  |
| Dioal.17G104800.1 | EC:2.3.1.74 | OG0000150 | Genome Annotation | GN=CHSJ PE=2 SV=2 | map00941 |
|  |  |  |  | sp\|P22928\|CHSJ_PETHY Chalcone synthase J OS=Petunia hybrida OX=4102 |  |
| Dioal.17G104900.1 | EC:2.3.1.74 | OG0000150 | Genome Annotation | GN=CHSJ PE=2 SV=2 | map00941 |
|  |  |  |  | sp\|P22928\|CHSJ_PETHY Chalcone synthase J OS=Petunia hybrida OX=4102 |  |
| Dioal.17G105000.1 | EC:2.3.1.74 | OG0000150 | Genome Annotation | GN=CHSJ PE=2 SV=2 | map00941 |
|  |  |  |  | sp\|P48386\|CHS1_CAMSI Chalcone synthase 1 OS=Camellia sinensis OX=4442 |  |
| Dioal.17G105100.1 | EC:2.3.1.74 | OG0000150 | Genome Annotation | GN=CHS1 PE=2 SV=1 | map00941 |
|  |  |  |  | sp\|P48387\|CHS2_CAMSI Chalcone synthase 2 OS=Camellia sinensis OX=4442 |  |
| Dioal.17G105200.1 | EC:2.3.1.74 | OG0000150 | Genome Annotation | GN=CHS2 PE=2 SV=1 | map00941 |
|  |  |  |  | sp\|Q9ZRR8\|CHS1_CASGL Chalcone synthase OS=Casuarina glauca OX=3522 |  |
| Dioal.17G105300.1 | EC:2.3.1.74 | OG0000150 | Genome Annotation | GN=CHS1 PE=2 SV=1 | map00941 |
|  |  |  |  | sp\|Q9ZRR8\|CHS1_CASGL Chalcone synthase OS=Casuarina glauca OX=3522 |  |
| Dioal.17G105400.1 | EC:2.3.1.74 | OG0000150 | Genome Annotation | GN=CHS1 PE=2 SV=1 | map00941 |
|  |  |  |  | sp\|Q9ZRR8\|CHS1_CASGL Chalcone synthase OS=Casuarina glauca OX=3522 |  |
| Dioal.17G105500.1 | EC:2.3.1.74 | OG0000150 | Genome Annotation | GN=CHS1 PE=2 SV=1 | map00941 |

Dioal.17G105600.1 EC:2.3.1.74 OG0000150 Genome Annotation Dioal.17G105700.1 EC:2.3.1.74 OG0000150 Genome Annotation Dioal.17G106300.1 EC:2.3.1.74 OG0000150 Genome Annotation Dioal.17G106500.1 EC:2.3.1.74 OG0000150 Genome Annotation Dioal.17G106700.1 EC:2.3.1.74 OG0000150 Genome Annotation Dioal.17G106800.1 EC:2.3.1.74 OG0000150 Genome Annotation Dioal.17G106900.1 EC:2.3.1.74 OG0000150 Genome Annotation Dioal.17G107000.1 EC:2.3.1.74 OG0000150 Genome Annotation Dioal.17G107100.1 EC:2.3.1.74 OG0000150 Genome Annotation Dioal.17G107200.1 EC:2.3.1.74 OG0000150 Genome Annotation Dioal.17G109200.1 OG0000290 Orthogroups

Dioal.17G109300.1 OG0000290 Orthogroups

Dioal.17G109500.1 OG0000290 Orthogroups Dioal.18G009000.1 EC:5.5.1.6 OG0005831 Genome Annotation Dioal.18G031000.1 EC:1.14.11.23 E OG0003171 Genome Annotation Dioal.18G044100.1 EC:2.3.1.133 OG0000415 Genome Annotation Dioal.18G048800.1 OG0000290 Orthogroups

sp|Q9XJ58|CHS1_CITSI Chalcone synthase 1 OS=Citrus sinensis OX=2711

GN=CHS1 PE=2 SV=1 map00941

sp|Q9ZRR8|CHS1_CASGL Chalcone synthase OS=Casuarina glauca OX=3522

GN=CHS1 PE=2 SV=1 map00941

sp|Q9ZRR8|CHS1_CASGL Chalcone synthase OS=Casuarina glauca OX=3522

GN=CHS1 PE=2 SV=1 map00941

sp|Q9ZRR8|CHS1_CASGL Chalcone synthase OS=Casuarina glauca OX=3522

GN=CHS1 PE=2 SV=1 map00941

sp|Q9ZRR8|CHS1_CASGL Chalcone synthase OS=Casuarina glauca OX=3522

GN=CHS1 PE=2 SV=1 map00941

sp|Q9ZRR8|CHS1_CASGL Chalcone synthase OS=Casuarina glauca OX=3522

GN=CHS1 PE=2 SV=1 map00941

sp|Q9XJ58|CHS1_CITSI Chalcone synthase 1 OS=Citrus sinensis OX=2711

GN=CHS1 PE=2 SV=1 map00941

sp|Q9ZRR8|CHS1_CASGL Chalcone synthase OS=Casuarina glauca OX=3522

GN=CHS1 PE=2 SV=1 map00941

sp|Q9XJ58|CHS1_CITSI Chalcone synthase 1 OS=Citrus sinensis OX=2711

GN=CHS1 PE=2 SV=1 map00941

sp|Q9ZRR8|CHS1_CASGL Chalcone synthase OS=Casuarina glauca OX=3522

GN=CHS1 PE=2 SV=1 map00941

sp|Q9AT54|SCGT_TOBAC Scopoletin glucosyltransferase OS=Nicotiana tabacum

OX=4097 GN=TOGT1 PE=1 SV=1 map00941

sp|Q9AT54|SCGT_TOBAC Scopoletin glucosyltransferase OS=Nicotiana tabacum

OX=4097 GN=TOGT1 PE=1 SV=1 map00941

sp|Q9AT54|SCGT_TOBAC Scopoletin glucosyltransferase OS=Nicotiana tabacum

OX=4097 GN=TOGT1 PE=1 SV=1 map00941

sp|Q84RK2|FAP2_ARATH Fatty-acid-binding protein 2 OS=Arabidopsis thaliana

OX=3702 GN=FAP2 PE=2 SV=2 map00941

sp|Q9ZWQ9|FLS_CITUN Flavonol synthase/flavanone 3-hydroxylase OS=Citrus

unshiu OX=55188 GN=FLS PE=1 SV=1 map00941

sp|Q8GSM7|HST_TOBAC Shikimate O-hydroxycinnamoyltransferase

OS=Nicotiana tabacum OX=4097 GN=HST PE=1 SV=1 map00941 sp|Q9AT54|SCGT_TOBAC Scopoletin glucosyltransferase OS=Nicotiana tabacum

OX=4097 GN=TOGT1 PE=1 SV=1 map00941

|  | | | | sp\|Q9SRM3\|DIOX6_ARATH Probable 2-oxoglutarate-dependent dioxygenase |  |
| --- | --- | --- | --- | --- | --- |
| Dioal.18G096100.1 | EC:1.14.11.9 | OG0000931 | Genome Annotation | At3g111800 OS=Arabidopsis thaliana OX=3702 GN=At3g11180 PE=2 SV=1 | map00941 |
|  |  |  |  | sp\|Q9FFF6\|DIOX5_ARATH Probable 2-oxoglutarate-dependent dioxygenase |  |
| Dioal.18G096200.1 |  | OG0000931 | Orthogroups | At5g05600 OS=Arabidopsis thaliana OX=3702 GN=At5g05600 PE=2 SV=1 | map00941 |
|  |  |  |  | sp\|Q9FFF6\|DIOX5_ARATH Probable 2-oxoglutarate-dependent dioxygenase |  |
| Dioal.18G096800.1 |  | OG0000931 | Orthogroups | At5g05600 OS=Arabidopsis thaliana OX=3702 GN=At5g05600 PE=2 SV=1 | map00941 |
|  |  |  |  | sp\|Q84V83\|LAR_DESUN Leucoanthocyanidin reductase OS=Desmodium |  |
| Dioal.19G048600.1 | EC:1.17.1.3 | OG0010523 | Genome Annotation | uncinatum OX=225101 GN=LAR PE=1 SV=1 | map00941 |
|  |  |  |  | sp\|Q5FB34\|ANRCS_VITVI Anthocyanidin reductase ((2S)-flavan-3-ol-forming) |  |
| Dioal.19G053900.1 | EC:1.3.1.77 | OG0003217 | Genome Annotation | OS=Vitis vinifera OX=29760 GN=ANR PE=1 SV=1 | map00941 |
|  |  |  |  | sp\|Q8W2X5\|FL3H2_ORYSJ Flavanone 3-dioxygenase 2 OS=Oryza sativa subsp. |  |
| Dioal.19G083800.1 | EC:1.14.11.9 | OG0000313 | Genome Annotation | japonica OX=39947 GN=F3H-2 PE=1 SV=1 | map00941 |
|  |  |  |  | sp\|Q9ZSA7\|DLO2_ARATH Protein DMR6-LIKE OXYGENASE 2 OS=Arabidopsis |  |
| Dioal.19G084100.1 | EC:1.14.11.9 | OG0000313 | Genome Annotation | thaliana OX=3702 GN=DLO2 PE=2 SV=1 | map00941 |
|  |  |  |  | sp\|Q9ZSA7\|DLO2_ARATH Protein DMR6-LIKE OXYGENASE 2 OS=Arabidopsis |  |
| Dioal.19G084200.1 | EC:1.14.11.9 | OG0000313 | Genome Annotation | thaliana OX=3702 GN=DLO2 PE=2 SV=1 | map00941 |
|  |  |  |  | sp\|Q9ZSA7\|DLO2_ARATH Protein DMR6-LIKE OXYGENASE 2 OS=Arabidopsis |  |
| Dioal.19G084300.1 | EC:1.14.11.9 | OG0000313 | Genome Annotation | thaliana OX=3702 GN=DLO2 PE=2 SV=1 | map00941 |
|  |  |  |  | sp\|Q8VZW3\|CFI3_ARATH Probable chalcone--flavonone isomerase 3 |  |
| Dioal.19G120300.1 | EC:5.5.1.6 | OG0009534 | Genome Annotation | OS=Arabidopsis thaliana OX=3702 GN=CHI3 PE=1 SV=1 | map00941 |
|  |  |  |  | sp\|Q9XES5\|DFRA_MALDO Bifunctional dihydroflavonol 4-reductase/flavanone 4- |  |
| Dioal.20G036100.1 | EC:1.1.1.219 | OG0003437 | Genome Annotation | reductase OS=Malus domestica OX=3750 GN=DFR PE=1 SV=1 | map00941 |
|  |  |  |  | sp\|Q9XES5\|DFRA_MALDO Bifunctional dihydroflavonol 4-reductase/flavanone 4- |  |
| Dioal.20G036200.1 | EC:1.1.1.219 | OG0003437 | Genome Annotation | reductase OS=Malus domestica OX=3750 GN=DFR PE=1 SV=1 sp\|P51106\|DFRA_HORVU Dihydroflavonol 4-reductase OS=Hordeum vulgare | map00941 |
| Dioal.20G036300.1 | EC:1.1.1.219 | OG0003437 | Genome Annotation | OX=4513 GN=ANT18 PE=3 SV=1 | map00941 |

**Supplementary Table 3** : Summary of species used in comparative genomics, family, proteins, and BUSCO score based on the Viridiplantae Odb10 dataset

| **Code** | **Species** | **Dowloaded from** | **Proteins** | **Number of Prot** | **BUSCO Score** |
| --- | --- | --- | --- | --- | --- |
| Ahyp | *Arachis hypogaea* | Phytozome v13 | 67124 | 64743 | 96,4 |
| Atha | *Arabidopsis thaliana* | Phytozome v13 | 27416 | 26023 | 99,5 |
| Atri | *Amborella trichopoda* | Ensemble | 27313 | 23503 | 95,3 |
| Bdis | *Brachypodium distachyon* | Phytozome v13 | 32439 | 28283 | 99,5 |
| Bole | *Brassica oleracea* | Phytozome v13 | 35400 | 33741 | 79,1 |
| Brap | *Brassica rapa* | Phytozome v13 | 40492 | 39467 | 97,2 |
| Bvul | *Beta vulgaris* | Phytozome v13 | 24255 | 22686 | 95 |
| Cari | *Cicer arietinum* | Phytozome v13 | 28269 | 27790 | 95 |
| Ccle | *Citrus clementina* | Phytozome v13 | 24533 | 24040 | 97,4 |
| Cesc | *Colocasia esculenta* | NCBI | 56238 | 23669 | 69,9 |
| Ccan | *Coffea canephora* | Ensemble | 25574 | 53623 | 93,6 |
| Csat | *Cucumis sativus* | Phytozome v13 | 21503 | 20090 | 95,5 |
| Csin | *Camellia sinensis* | Phytozome v13 | 25379 | 24424 | 88,7 |
| Dala | *Dioscorea alata* | Phytozome v13 | 25189 | 24577 | 97,4 |
| Dcar | *Daucus carota* | Phytozome v13 | 32113 | 30350 | 87,1 |
| Ddum | *Dioscorea dumetorum* | bielefeld.de/record/2941469 | 35681 | 32193 | 87,3 |
| Drot | *Dioscorea rotundata* | [http://genome-e.ibrc.or.jp](http://genome-e.ibrc.or.jp/) | 64831 | 30516 | 79,3 |
| Dzin | *Dioscorea zingiberensis* | baidu | 26033 | 24551 | 92,7 |
|  |  | [http://palmxplore.mpob.gov.my](http://palmxplore.mpob.gov.my/) |  |  |  |
| Egui | *Elaeis guinnensis* | /palmXplore/download | 26059 | 25625 | 88,2 |
| Fves | *Fragaria vesca* | Phytozome v13 | 34006 | 31815 | 98,6 |
| Ghir | *Gossypium hirsutum* | Phytozome v13 | 75376 | 73584 | 99,7 |
| Gmax | *Glycine max* | Phytozome v13 | 52872 | 48610 | 99,5 |
| Grai | *Gossypium raimondii* | Phytozome v13 | 37505 | 37189 | 99,1 |
| Hvul | *Hordeum vulgare* | Phytozome v13 | 39734 | 38292 | 81,4 |
| Lalb | *Lupinus albus* | Phytozome v13 | 38258 | 35724 | 96,7 |
| Macu | *Musa acuminata* | banana-hub | 32692 | 28712 | 71,3 |
| Mdom | *Malus domestica* | Phytozome v13 | 45116 | 41542 | 97,2 |
| Mesc | *Manihot esculenta* | Phytozome v13 | 32805 | 31482 | 99,8 |
| Mtru | *Medicago truncatula* | Phytozome v13 | 50894 | 45551 | 97 |
| Osat | *Oryza sativa* | Phytozome v13 | 42189 | 36101 | 93,2 |
| Otho | *Oropetium thomaeum* | Phytozome v13 | 28437 | 23956 | 79,3 |
| Pdat | *Phoenix dactylifera* | Phytozome v13 | 57599 | 55916 | 94,8 |
| Pequi | *Phalaenopsis equestris* | Phytozome v13 | 56343 | 53207 | 96,5 |
| Ptri | *Populus trichocarpa* | Phytozome v13 | 34699 | 32991 | 99,6 |
| Pvul | *Phaseolus vulgaris* | Phytozome v13 | 27433 | 26884 | 99 |
| Sbic | *Sorghum bicolor* | Phytozome v13 | 34129 | 30021 | 99,5 |
| Sita | *Setaria italica* | Phytozome v13 | 34584 | 31787 | 98,8 |
| Slyc | *Solanum lycopersicum* | Phytozome v13 | 35768 | 32092 | 94,8 |
| Stub | *Solanum tuberosum* | Phytozome v13 | 39028 | 36990 | 76,7 |
| Taes | *Triticum aestivum* | Phytozome v13 | 99386 | 93318 | 88,2 |
| Tcac | *Theobroma cacao* | Phytozome v13 | 27379 | 24341 | 99,7 |
| Tzey | *Trichopus zeylanicus* | NCBI | 34452 | 31435 | 63 |
| Vung | *Vigna unguiculata* | Phytozome v13 | 31948 | 30542 | 99,5 |
| Vvin | *Vitis vinifera* | Phytozome v13 | 31845 | 28571 | 96,3 |

Zmay *Zea mays* Phytozome v13 39498 37969 94,3

**Supplementary Table 4** : Orthogroups specific of *D. alata* not found in other Dioscorea

| **OG** | **Gene Model** | **PFAM** | **EC Number** | **Panther** | **KOG** | **Kegg** | **Gene Ontology Oryza sativa Orthologous** |
| --- | --- | --- | --- | --- | --- | --- | --- |
|  |  | PF00082  PF02225 |  | PTHR10795 PTHR10795 |  |  | GO:0004252 |
| OG0010389: | Dioal.02G093700.1.p | PF05922 | EC:3.4.21.25 | :SF329 |  |  | GO:0006508 |
|  |  | PF00082  PF02225 |  | PTHR10795 PTHR10795 |  |  | GO:0004252 |
| OG0010389: | Dioal.02G093800.1.p | PF05922 | EC:3.4.21.25 | :SF370 |  |  | GO:0006508 |
|  |  | PF00082  PF02225 |  | PTHR10795 PTHR10795 |  |  | GO:0004252 |
| OG0010389: | Dioal.02G094000.1.p | PF05922 | EC:3.4.21.25 | :SF329 |  |  | GO:0006508 |
|  |  | PF00082  PF02225 |  | PTHR10795 PTHR10795 |  |  | GO:0004252 |
| OG0010389: | Dioal.02G094100.1.p | PF05922 | EC:3.4.21.25 | :SF370 |  |  | GO:0006508 |
|  |  | PF00082  PF02225 |  | PTHR10795 PTHR10795 |  |  | GO:0004252 |
| OG0010389: | Dioal.02G094300.1.p | PF05922 | EC:3.4.21.25 | :SF329 |  |  | GO:0006508 |
|  |  | PF00082  PF02225 |  | PTHR10795 PTHR10795 |  |  | GO:0004252 |
| OG0018487: | Dioal.02G096700.1.p | PF05922 | EC:3.4.21.25 | :SF370 |  |  | GO:0006508 |
|  |  | PF00082  PF02225 |  | PTHR10795 PTHR10795 |  |  | GO:0004252 |
| OG0018487: | Dioal.02G096800.1.p | PF05922 | EC:3.4.21.25 | :SF370 |  |  | GO:0006508 |
|  |  | PF00082  PF02225 |  | PTHR10795 PTHR10795 |  |  | GO:0004252 |
| OG0018487: | Dioal.02G096900.1.p | PF05922 | EC:3.4.21.25 | :SF370 |  |  | GO:0006508 |

| OG0023926: | Dioal.04G083300.1.p | PF00320 |  | PTHR10071 PTHR10071  :SF208 |  | GO:0003700  GO:0006355  GO:0008270  GO:0043565 |  |
| --- | --- | --- | --- | --- | --- | --- | --- |
|  |  |  |  | PTHR11804 PTHR11804 |  | GO:0004222 |  |
| OG0020693: | Dioal.05G157700.1.p | PF01432 | EC:3.4.24.70 | :SF49  PTHR31673 | KOG2089 | GO:0006508  GO:0004553  GO:0010215  GO:0016049 |  |
| OG0026507: | Dioal.05G166300.1.p | PF04833 |  | PTHR31673  :SF3 |  | GO:0030247  GO:0031225 |  |
| OG0014785: | Dioal.06G069500.1.p |  |  | PTHR33088 PTHR33088  :SF5 |  |  | LOC_Os08g37300 |
| OG0014785: | Dioal.06G069700.1.p |  |  | PTHR33088 PTHR33088  :SF5 |  |  | LOC_Os08g37300 |
| OG0014785: | Dioal.06G069800.1.p |  |  | PTHR33088 |  |  | LOC_Os08g37300 |
| OG0014785: | Dioal.06G069900.1.p |  |  | PTHR33088 PTHR33088  :SF5 |  |  | LOC_Os08g37300 |
| OG0014785: | Dioal.06G070000.1.p |  |  | PTHR33088 PTHR33088  :SF5 |  |  | LOC_Os08g37300 |

| OG0014785: | Dioal.06G070100.1.p |  |  | PTHR33088 PTHR33088  :SF5 |  | LOC_Os08g37300 |
| --- | --- | --- | --- | --- | --- | --- |
| OG0014785: | Dioal.06G070200.1.p |  |  | PTHR33088 PTHR33088  :SF5 |  | LOC_Os08g37300 |
| OG0014785: | Dioal.06G070400.1.p |  |  | PTHR33088 PTHR33088  :SF13 |  |  |
|  |  | PF00082 |  | PTHR10795 PTHR10795 | GO:0004252 |  |
| OG0010389: | Dioal.07G079500.1.p | PF02225 | EC:3.4.14.10 | :SF329 | GO:0006508 |  |
|  |  | PF00082  PF02225 |  | PTHR10795 PTHR10795 | GO:0004252 |  |
| OG0010389: | Dioal.07G079600.1.p | PF05922 | EC:3.4.14.10 | :SF329 | GO:0006508 |  |
|  |  | PF00082  PF02225 |  | PTHR10795 PTHR10795 | GO:0004252 |  |
| OG0010389: | Dioal.09G036400.1.p | PF05922 | EC:3.4.21.25 | :SF329 | GO:0006508 |  |
|  |  | PF00082  PF02225 |  | PTHR10795 PTHR10795 | GO:0004252 |  |
| OG0010389: | Dioal.09G036700.1.p | PF05922 | EC:3.4.21.25 | :SF329 | GO:0006508 |  |
|  |  | PF00082  PF02225 |  | PTHR10795 PTHR10795 | GO:0004252 |  |
| OG0010389: | Dioal.09G037100.1.p | PF05922 | EC:3.4.21.25 | :SF329 | GO:0006508 |  |

| OG0023931: | Dioal.09G053500.1.p | PF03357 |  | PTHR22761 PTHR22761  :SF8 | KOG1656 |  | GO:0007034 |
| --- | --- | --- | --- | --- | --- | --- | --- |
|  |  | PF00082  PF02225 |  | PTHR10795 PTHR10795 |  |  | GO:0004252 |
| OG0010389: | Dioal.10G008900.1.p | PF05922 | EC:3.4.21.25 | :SF329 |  |  | GO:0006508 |
|  |  | PF00082 |  | PTHR10795 PTHR10795 |  |  | GO:0004252 |
| OG0010389: | Dioal.10G009100.1.p | PF05922 | EC:3.4.21.25 | :SF370 |  |  | GO:0006508 |
| OG0023905: | Dioal.10G042900.1.p | PF06521 |  | PTHR33649 |  |  |  |
|  |  | PF00082 |  | PTHR10795 PTHR10795 |  |  | GO:0004252 |
| OG0010389: | Dioal.13G064900.1.p | PF02225 | EC:3.4.21.25 | :SF370 |  |  | GO:0006508 |
| OG0022130: | Dioal.14G044500.1.p |  |  |  |  |  |  |
|  |  |  |  | PTHR11926 PTHR11926 |  |  | GO:0008152 |
| OG0018480: | Dioal.14G113600.1.p | PF00201 |  | :SF381 |  | K08237 | GO:0016758 |
|  |  |  |  | PTHR11926 PTHR11926 |  |  | GO:0008152 |
| OG0018480: | Dioal.17G071300.1.p | PF00201 |  | :SF381 |  | K08237 | GO:0016758 |
|  |  |  |  | PTHR11926 PTHR11926 |  |  | GO:0008152 |
| OG0018480: | Dioal.17G071400.1.p | PF00201 |  | :SF381 |  |  | GO:0016758 |

|  | | | | | GO:0003824 |
| --- | --- | --- | --- | --- | --- |
|  |  |  |  |  | GO:0006633 |
|  |  |  |  |  | GO:0008152 |
|  |  |  |  |  | GO:0008610 |
|  |  |  |  |  | GO:0016020 |
| OG0018476: | Dioal.18G049100.1.p | PF08541 | EC:2.3.1.199 | PTHR31561 | GO:0016747 |
|  |  |  |  |  | GO:0003824 |
|  |  |  |  |  | GO:0006633 |
|  |  |  |  |  | GO:0008152 |
|  |  |  |  |  | GO:0008610 |
|  |  |  |  |  | GO:0016020 |
| OG0018476: | Dioal.18G049300.1.p | PF08541 | EC:2.3.1.199 | PTHR31561 | GO:0016747 |
|  |  |  |  | PTHR31791 | GO:0003677 |
|  |  |  |  | PTHR31791 | GO:0005634 |
| OG0026498: | Dioal.19G018100.1.p | PF07899 |  | :SF4 | GO:0006351 |
|  |  | PF03080 |  |  |  |
| OG0019480: | Dioal.19G037400.1.p | PF14365 |  | PTHR31589 |  |
|  |  |  |  |  | GO:0004097 |
|  |  | PF00264 |  | PTHR11474 | GO:0008152 |
|  |  | PF12142 |  | PTHR11474 | GO:0016491 |
| OG0020689: | Dioal.19G089300.1.p | PF12143 | EC:1.10.3.1 | :SF33 | GO:0055114 |
| OG0020689: | Dioal.19G089400.1.p | PF00264 | EC:1.10.3.1 | PTHR11474 | GO:0004097 |

**Supplementary Table 5** : Genes found on the Fst analysis. Gene models under selection, functional annotation, cluster were the gene was found, number of map Kegg Pathway and the method by which the gene was found

| **Gene Model** | **Function Annotation** | **Clusters** | **KEGG Pathway** | **Found by** |
| --- | --- | --- | --- | --- |
| Dioal.01G016100 | Probable 2-oxoglutarate-dependent dioxygenase | Cluster2xCluster3 | map00941 | Keyword |
| Dioal.01G016200 | Probable 2-oxoglutarate-dependent dioxygenase | Cluster2xCluster3 | map00941 | Keyword |
| Dioal.01G025700 | Probable pectate lyase | Cluster2xCluster3 | map00040 | Keyword |
| Dioal.01G025800 | Probable pectate lyase | Cluster2xCluster3 | map00040 | Keyword |
| Dioal.01G035100 | Probable pectate lyase | Cluster2xCluster3 | map00040 | Keyword |
| Dioal.02G074500 | Putative pentatricopeptide repeat-containing protein | Cluster2xCluster3 | map00040 | Orthology |
| Dioal.04G168200 | Beta-amylase | Cluster1xCluster3 | map00500 | Keyword |
| Dioal.04G184200 | Probable trehalose-phosphate | Cluster2xCluster3 | map00500 | Keyword |
| Dioal.06G005800 | Polygalacturonase | Cluster2xCluster3 | map00040 | Keyword |
| Dioal.08G105700 | Pectine methylsterase | Cluster2xCluster3 | map00040 | Keyword |
| Dioal.08G106000 | Cellulose synthase | Cluster2xCluster3 | map00500 | Orthology |
| Dioal.08G106500 | Putrescine hydroxycinnamoyltransferase | Cluster2xCluster3 | map00941 | Keyword |
| Dioal.08G106600 | Putrescine hydroxycinnamoyltransferase | Cluster2xCluster3 | map00941 | Keyword |
| Dioal.08G106800 | Putrescine hydroxycinnamoyltransferase | Cluster2xCluster3 | map00941 | Orthology |
| Dioal.08G106900 | Putrescine hydroxycinnamoyltransferase | Cluster2xCluster3 | map00941 | Orthology |
| Dioal.08G107000 | Hydroxycinnamoyltransferase | Cluster2xCluster3 | map00941 | Keyword |
| Dioal.08G107100 | Hydroxycinnamoyltransferase | Cluster2xCluster3 | map00941 | Keyword |
| Dioal.08G130500 | Probable cellulose synthase | Cluster2xCluster3 | map00500 | Orthology |
| Dioal.08G131200 | Fatty-acid-binding protein | Cluster2xCluster3 | map00941 | Keyword |
| Dioal.09G032200 | Soluble map00500 synthase | Cluster2xCluster3 | map00500 | Keyword |
| Dioal.09G032200 | Soluble map00500 synthase | Cluster1xCluster3 | map00500 | Keyword |
| Dioal.10G014800 | 1,3-beta-Glucan synthase | Cluster1xCluster2 | map00500 | Keyword |
| Dioal.10G018800 | Norbelladine 4'-O-methyltransferase | Cluster1xCluster2 | map00941 | Keyword |
| Dioal.11G000200 | Nudix hydrolase | Cluster2xCluster3 | map00500 | Keyword |
| Dioal.11G000200 | Nudix hydrolase | Cluster1xCluster2 | map00500 | Keyword |
| Dioal.20G055000 | Malectin | Cluster2xCluster3 | map00040 | Orthology |
| Dioal.20G055100 | Malectin | Cluster2xCluster3 | map00040 | Orthology |
| Dioal.20G055200 | Malectin | Cluster2xCluster3 | map00040 | Orthology |
| Dioal.20G055300 | Malectin | Cluster2xCluster3 | map00040 | Orthology |

**Supplementary Table 6** : Descriptive statistics of tuber quality traits of the association population

**Traits Mean±SDa Range CV (%)b Skewness Kurtosis**

| **Color BI** | 41.06±25.44 12.57-93.00 61.96 1.92 4.15 | | | | |
| --- | --- | --- | --- | --- | --- |
| **HI** | 81.37±23.45 | -11.99-99.92 | 0.29 | -2.35 | 4,6 |
| **Color changes BI_dif** | 4.86±7.84 | -14.66-54.45 | 161.25 | 2.60 | 15.03 |
| **HI_dif** | 0,38±2,41 | -21.35-8.9 | 642.8 | -3.67 | 29.10 |
| **SC (%)** | 77.93±2.10 | 69.46-81.26 | 2.60 | -1.28 | 1.91 |
| **Total area (N.sec) Hardness** | 45.06±16.91  28.64±16.80 | 14.14-90.69  6.39-85.73 | 37.52  58.64 | 0.88  1.33 | 0.77  1.99 |
| **Texture Cohesiveness** | 0.20±0.04 | 0.13-0.35 | 20.91 | 1.25 | 2.81 |
| **Gumminess** | 5.90±4.88 | 1.27-31.06 | 82.78 | 3.33 | 15.01 |
| **Springiness** | 1.02±0.22 | 0.66-1.63 | 21.46 | 0.54 | -0.16 |

a mean±standard deviation of single year/location phenotypic data. b coefficient of variation. c BI = brown index and HI = Hue index; SC = map00500 content.

**Supplemental Table 7** : Normalized TPM values for all genes found in the three KEGG pathways for the six genotypes of *D. alata* . The gene model, the normalized expression values (-LOG2) and the metabolic kegg pathway for each gene identified.

| **Gene** | **CRB47** | **CRB96** | **Roujol49** | **Roujol62** | **Roujol75** | **Roujol9** | **Metabolic Pathway** |
| --- | --- | --- | --- | --- | --- | --- | --- |
| Dioal.01G024300.1 | -3,12247 | -3,12247 | -4,21258 | 0 | -4,48136 | 0 | map00040 |
| Dioal.01G024400.1 | 0 | 0 | 0 | 0 | 0 | 0 | map00040 |
| Dioal.01G024600.1 | 0 | 0 | -3,48166 | 0 | 0 | 0 | map00040 |
| Dioal.01G024700.1 | 0 | 0 | 0 | 0 | 0 | 0 | map00040 |
| Dioal.01G024800.1 | 0 | 0 | 0 | 0 | 0 | 0 | map00040 |
| Dioal.01G025700.1 | 0 | 0 | -1,89264 | 0 | 0 | 0 | map00040 |
| Dioal.01G025800.1 | -1,29433 | -1,29433 | -1,89264 | -1,45034 | 0 | 0 | map00040 |
| Dioal.01G032100.1 | -2,29429 | -2,29429 | -1,64687 | 0 | 0 | 0 | map00040 |
| Dioal.01G035100.1 | -0,99044 | -0,99044 | 0 | 0 | 0 | 0 | map00040 |
| Dioal.01G036500.1 | -1,64321 | -1,64321 | -0,689053 | -0,670397 | -0,991017 | -1,53791 | map00040 |
| Dioal.01G036800.1 | -3,97208 | -3,97208 | -2,6554 | -1,63492 | -2,33842 | -2,16642 | map00040 |
| Dioal.01G079100.1 | -1,23844 | -1,23844 | -0,71809 | -0,859487 | 2,0183 | -0,0601237 | map00040 |
| Dioal.01G079500.1 | 0,220493 0,220493 | | 0,240697 0,199836 1,12014 | | | 1,14355 | map00040 |
| Dioal.01G084900.1 | -4,1644 -4,1644 | | -2,79186 0 0 | | | 0 | map00040 |
| Dioal.01G090800.1 | -1,00186 | -1,00186 | -0,869874 | -1,72271 | -0,533383 | -1,34541 | map00040 |
| Dioal.02G029400.1 | -4,79753 | -4,79753 | -3,28598 | -3,2394 | -2,2534 | -1,92006 | map00040 |
| Dioal.02G041200.1 | -2,13338 | -2,13338 | 0 | -2,07794 | 0 | -2,37404 | map00040 |
| Dioal.02G059800.1 | 2,82718 | 2,82718 | 2,13011 | 2,43605 | 2,51855 | 2,68107 | map00040 |
| Dioal.02G061100.1 | -5,35614 | -5,35614 | -5,91983 | -1,84662 | -4,80384 | -5,94479 | map00040 |
| Dioal.02G061200.1 | 0 | 0 | 0 | -3,12009 | 0 | -2,13663 | map00040 |
| Dioal.02G061300.1 | -0,98005 | -0,98005 | -1,8123 | -2,96468 | -2,95116 | -3,02003 | map00040 |
| Dioal.02G061400.1 | -3,53913 | -3,53913 | -1,7142 | -2,1187 | -3,82262 | -1,76921 | map00040 |
| Dioal.02G061500.1 | 0 0 | | -5,06918 | 0 | -2,03635 | -1,74795 | map00040 |
| Dioal.02G061600.1 | -3,36309 | -3,36309 | -2,50268 | 0 | -5,17535 | -5,12726 | map00040 |
| Dioal.02G061800.1 | 0 | 0 | 0 | 0 | 0 | 0 | map00040 |
| Dioal.02G061900.1 | 0 | 0 | 0 | 0 | 0 | 0 | map00040 |
| Dioal.02G062000.1 | -7,32753 | -7,32753 | -3,55999 | -1,60248 | -3,65638 | -1,46267 | map00040 |
| Dioal.02G062100.1 | -2,00911 | -2,00911 | -4,94654 | -5,62536 | -5,38914 | -2,69316 | map00040 |

| Dioal.02G062200.1 | 0 | 0 | -2,09239 | 0 | -1,7163 | -3,7836 | map00040 |
| --- | --- | --- | --- | --- | --- | --- | --- |
| Dioal.02G062400.1 | -3,62498 | -3,62498 | 0 | -2,04195 | 0 | 0 | map00040 |
| Dioal.02G062500.1 | -3,51784 | -3,51784 | -1,1901 | -1,60056 | -2,99012 | -1,96116 | map00040 |
| Dioal.02G062700.1 | -4,64868 | -4,64868 | -3,65903 | 0 | -5,413 | -4,06364 | map00040 |
| Dioal.02G062800.1 | -2,05416 | -2,05416 | -5,08796 | -3,50561 | -0,333295 | -3,67071 | map00040 |
| Dioal.02G064200.1 | -1,5255 | -1,5255 | 0 | -2,02491 | -4,22305 | -3,79178 | map00040 |
| Dioal.02G064300.1 | 4,62774 | 4,62774 | 3,98522 | -0,85763 | -0,587017 | -1,18531 | map00040 |
| Dioal.02G064400.1 | -2,10442 | -2,10442 | -1,45792 | -2,53784 | -3,67919 | -2,89175 | map00040 |
| Dioal.02G064600.1 | -2,37366 | -2,37366 | -3,08464 | -1,83078 | -1,31463 | -2,36754 | map00040 |
| Dioal.02G064800.1 | -2,43895 | -2,43895 | 0 | 0 | 0 | 0 | map00040 |
| Dioal.02G064900.1 | 0 | 0 | 0 | 0 | 0 | -1,84357 | map00040 |
| Dioal.02G065000.1 | 0 | 0 | 0 | 0 | -1,65725 | -2,3178 | map00040 |
| Dioal.02G065100.1 | -2,88662 | -2,88662 | 0 | -1,91125 | 0 | -1,79784 | map00040 |
| Dioal.02G065200.1 | -3,12085 | -3,12085 | 0 | -5,41612 | 0 | 0 | map00040 |
| Dioal.02G065300.1 | -0,156162 | -0,156162 | -5,15076 | 0,0618487 | -3,68018 | -1,26486 | map00040 |
| Dioal.02G066000.1 | -1,16742 | -1,16742 | -4,02429 | 0,482882 | -4,26075 | -0,108756 | map00040 |
| Dioal.02G066200.1 | 0 | 0 | -1,86723 | -1,21599 | -2,56281 | -1,17807 | map00040 |
| Dioal.02G066400.1 | -2,15186 | -2,15186 | -2,79767 | -0,806932 | -3,52153 | -1,29655 | map00040 |
| Dioal.02G066500.1 | -1,76892 | -1,76892 | -1,00861 | -0,08053 | -1,01019 | -0,110851 | map00040 |
| Dioal.02G066700.1 | -1,66041 | -1,66041 | -4,07122 | -1,26235 | -1,7045 | -0,88775 | map00040 |
| Dioal.02G066900.1 | -1,09837 | -1,09837 | -0,804612 | -0,070661 | -1,83528 | -0,303289 | map00040 |
| Dioal.02G067100.1 | -3,03355 | -3,03355 | -2,97157 | -2,24931 | -1,90554 | -2,57231 | map00040 |
| Dioal.02G067200.1 | 0 | 0 | 0 | 0,733213 | -0,246936 | 0,988443 | map00040 |
| Dioal.02G067400.1 | -5,21212 | -5,21212 | -3,24159 | -1,87985 | -3,9448 | -1,92214 | map00040 |
| Dioal.02G071200.1 | 0 | 0 | 0 | 0 | -1,74516 | 0 | map00040 |
| Dioal.02G071300.1 | 0 | 0 | 0 | 0 | 0 | 0 | map00040 |
| Dioal.02G074500.1 | 1,25778 | 1,25778 | 1,07618 | 1,1258 | 1,37197 | 0,893529 | map00040 |
| Dioal.03G015800.1 | 5,39587 | 5,39587 | 5,77647 | 5,58564 | 5,70906 | 5,97013 | map00040 |
| Dioal.03G035700.1 | 1,44092 | 1,44092 | 1,99012 | 2,84267 | 1,73971 | 2,41015 | map00040 |
| Dioal.03G049500.1 | -4,15541 | -4,15541 | -1,61462 | -2,01164 | 0 -4,04546 map00040 | | |
| Dioal.03G088200.1 | -2,73967 | -2,73967 | -0,290999 | -1,24055 | -2,63123 | 0,304854 | map00040 |
| Dioal.04G018700.1 | -1,62767 | -1,62767 | -0,9772 | -1,45034 | 0,196888 | 0 | map00040 |

| Dioal.04G027900.1 | -1,5202 | -1,5202 | 0,925418 | 0,801953 | 2,10171 | -0,689827 | map00040 |
| --- | --- | --- | --- | --- | --- | --- | --- |
| Dioal.04G096300.1 | 0,609472 | 0,609472 | -0,84784 | -1,07253 | -1,32493 | -0,996035 | map00040 |
| Dioal.04G101400.1 | 0 | 0 | 0 | 0 | -1,56599 | 0 | map00040 |
| Dioal.04G130000.1 | 0 | 0 | 0 | 0 | 0 | 0 | map00040 |
| Dioal.04G130600.1 | 0 | 0 | -0,875027 | -0,543897 | -1,78475 | -0,702361 | map00040 |
| Dioal.04G130700.1 | 1,65122 | 1,65122 | -2,80955 | 1,5929 | 0,489585 | 0,650483 | map00040 |
| Dioal.04G130800.1 | 0 | 0 | 0,252804 | -1,59898 | 0 | -1,57768 | map00040 |
| Dioal.04G130900.1 | 0 | 0 | 0 | -0,893117 | 0 | 0 | map00040 |
| Dioal.04G131000.1 | 0 | 0 | -0,317025 | 0 | 0 | -0,0790567 | map00040 |
| Dioal.04G141500.1 | 0 | 0 | 0 | 0 | 0 | 0 | map00040 |
| Dioal.04G180800.1 | -1,7331 | -1,7331 | -1,37114 | -3,13832 | -3,6438 | -1,92003 | map00040 |
| Dioal.05G026200.1 | 2,86128 | 2,86128 | 3,11172 | 4,61207 | 3,41796 | 3,90408 | map00040 |
| Dioal.05G026300.1 | -0,468681 | -0,468681 | 1,12694 | 1,50499 | 0,359553 | 0,647636 | map00040 |
| Dioal.05G044700.1 | 0 | 0 | 0 | -2,76706 | -1,4484 | 0 | map00040 |
| Dioal.05G046500.1 | 0 | 0 | 0 | 0,225747 | 0,0642793 | -0,694767 | map00040 |
| Dioal.05G064400.1 | 0 | 0 | -0,81347 | -2,11064 | -1,11439 | -2,75724 | map00040 |
| Dioal.05G072900.1 | 3,1561 | 3,1561 | 0,314863 | -3,31611 | -1,00977 | -1,99657 | map00040 |
| Dioal.05G087200.1 | 0 | 0 | 0 | -1,10472 | 0 | 0 | map00040 |
| Dioal.05G105900.1 | 0 | 0 | 0 | 0 | 0 | 0 | map00040 |
| Dioal.05G141700.1 | -1,76068 | -1,76068 | -1,73213 | 0 | -1,09737 | 0 | map00040 |
| Dioal.05G183500.1 | 0,864282 | 0,864282 | 0,664524 | 0,818729 | -0,284167 | 0,156268 | map00040 |
| Dioal.05G185100.1 | 0,144589 | 0,144589 | 0,800494 | -0,648032 | -0,483416 | -0,513506 | map00040 |
| Dioal.05G191700.1 | 4,31525 | 4,31525 | 5,78 | 5,31768 | 4,25932 | 4,77997 | map00040 |
| Dioal.05G197800.1 | 0 | 0 | 0 | -1,45059 | 0,0497457 | -1,58538 | map00040 |
| Dioal.05G197900.1 | 0 0 | | 0 | 0 | 0 | 0 | map00040 |
| Dioal.05G198000.1 | -1,85009 | -1,85009 | -3,26165 | -3,0063 | -1,20378 | 0 | map00040 |
| Dioal.05G213900.1 | 0 | 0 | 0 | 0 | 0 | 0 | map00040 |
| Dioal.05G232900.1 | 0 0 | | 0 | 0 | 0 0 map00040 | | |
| Dioal.05G234400.1 | -2,93242 -2,93242 | | -2,50007 | 0,103631 | 0,88926 -3,52316 map00040 | | |
| Dioal.05G234500.1 | 3,58611 | 3,58611 | 3,57284 | 4,26805 | 4,75276 | 4,20117 | map00040 |
| Dioal.05G234600.1 | 0 | 0 | 0 | -0,310498 | -4,35165 | -2,43254 | map00040 |
| Dioal.05G236800.1 | 0 | 0 | 0 | 0 | 0 | 0 | map00040 |

Dioal.06G005800.1 Dioal.06G011700.1 Dioal.06G013000.1 Dioal.06G024200.1 Dioal.06G033200.1 Dioal.06G057500.1 Dioal.06G072100.1 Dioal.06G072400.1 Dioal.06G093700.1 Dioal.06G094500.1 Dioal.06G096600.1 Dioal.06G096700.1 Dioal.06G096800.1 Dioal.07G008700.1 Dioal.07G012800.1 Dioal.07G015800.1 Dioal.07G018800.1 Dioal.07G019000.1 Dioal.07G026400.1 Dioal.07G026500.1 Dioal.07G026600.1 Dioal.07G027000.1 Dioal.07G037500.1 Dioal.07G045100.1 Dioal.07G068800.1 Dioal.07G101200.1 Dioal.08G021600.1 Dioal.08G045800.1 Dioal.08G050900.1 Dioal.08G080700.1 Dioal.08G080800.1 Dioal.08G081100.1

| 0 | 0 | -1,74849 | 0 | 0 | 0 | |
| --- | --- | --- | --- | --- | --- | --- |
| 0 | 0 | 0 | 0 | 0 | 0 | |
| 0 | 0 | -2,79264 | -2,06852 | 0,268747 | -2,27045 | |
| 2,74566 | 2,74566 | 2,57776 | 3,58139 | 3,46799 | 3,4505 | |
| 2,28036 | 2,28036 | 2,3593 | 1,59029 | 1,93682 | 1,65127 | |
| -4,79019 | -4,79019 | -0,217419 | -3,04384 | -2,78904 | -3,15717 | |
| 0 | 0 | 0 | 0 | 0 | 0 | |
| 0 | 0 | -3,23929 | 0 | 0 | 0 | |
| -0,947273 | -0,947273 | -0,211383 | 0,22241 | -0,556194 | 0,796207 | |
| 0 | 0 | 0 | 0 | 0 | 0 | |
| -1,1898 | -1,1898 | 1,25303 | 0,631218 | -0,931582 | -2,63198 | |
| 0,639728 | 0,639728 | 3,89616 | 2,479 | 1,86663 | 0,892602 | |
| 3,19483 | 3,19483 | 1,5686 | 4,89872 | 2,97797 | 3,5324 | |
| -1,78555 | -1,78555 | 0 | -1,64717 | -1,85467 | -0,950163 | |
| 0 | 0 | 0 | -1,53975 | 0 | 0 | |
| 0 | 0 | 0 | 0 | 0 | 0 | |
| 0 | 0 | 0 | 0 | 0 | 0 | |
| 0 | 0 | 0 | 0 | 0 | 0 | |
| 0 | 0 | 0 | 0 | 0 | 0 | |
| 0 | 0 | 0 | -2,18657 | -2,80384 | | 0 |
| -2,65758 | -2,65758 | -0,836437 | -4,12895 | -3,04957 | | -2,37186 |
| 0  1,08117  0,699697 | 0  1,08117  0,699697 | 0  0,272183  0,593542 | 0  1,62997  2,38631 | 0  0,638063  1,90135 | 0  1,48194  2,10503 | |
| -1,43377 -1,43377 | | 0 | -3,38124 | -2,95731 | -3,94055 | |
| -3,73443 | -3,73443 | -3,81597 | -3,87578 | -3,63808 | -3,80532 | |
| 1,33011  0  0,826161 | 1,33011  0  0,826161 | 2,41014  0  -1,13789 | 2,32449  0  0,198981 | 1,21666  0  1,08551 | 1,68487  0  0,59498 | |
| -2,22348 | -2,22348 | -3,78321 | -1,85595 | -0,619421 | -0,675188 | |
| -4,25375 | -4,25375 | -3,02704 | -3,05894 | -2,4489 -1,74291 | | |
| -2,14033 | -2,14033 | -1,77922 | -4,55469 | -4,27811 | | -1,45504 |

map00040 map00040 map00040 map00040 map00040 map00040 map00040 map00040 map00040 map00040 map00040 map00040 map00040 map00040 map00040 map00040 map00040 map00040 map00040 map00040 map00040 map00040 map00040 map00040 map00040 map00040 map00040 map00040 map00040 map00040 map00040 map00040

Dioal.08G081200.1 Dioal.08G089400.1 Dioal.08G105700.1 Dioal.08G114300.1 Dioal.09G009200.1 Dioal.09G066900.1 Dioal.09G071100.1 Dioal.09G071800.1 Dioal.10G020100.1 Dioal.10G043000.1 Dioal.10G055200.1 Dioal.10G055300.1 Dioal.11G009600.1 Dioal.11G019600.1 Dioal.11G025100.1 Dioal.11G040500.1 Dioal.11G061100.1 Dioal.11G072500.1 Dioal.11G077600.1 Dioal.11G077700.1 Dioal.11G077800.1 Dioal.11G077900.1 Dioal.11G078100.1 Dioal.11G078200.1 Dioal.11G078300.1 Dioal.11G078400.1 Dioal.11G078500.1 Dioal.11G078600.1 Dioal.11G078800.1 Dioal.11G079700.1 Dioal.11G079800.1 Dioal.11G080000.1

| -4,69899 -4,69899 -3,85693 | | -1,37044 -1,99756 -1,32785 | | |
| --- | --- | --- | --- | --- |
| 2,7859 2,7859 2,70643 2,97408 2,6457 2,84914  0 0 0 0 0 0  1,63866 1,63866 1,77658 1,80383 2,28638 -0,111023 | | | | |
| 3,33771 3,33771 3,12874 3,92869 2,69018 4,10524 | | | | |
| 0 0 0 0 0 0 | | | | |
| 3,96882 3,96882 3,53477 4,02673 4,45767 4,18204 | | | | |
| 0 0 0 0 0 0 | | | | |
| 5,57206 5,57206 6,17143 5,94919 6,07288 5,98927 | | | | |
| 0 0 | -3,87974 | -1,56062 -1,63106 -1,88576 | | |
| -2,3904 -2,3904 | 0 -0,273305 0 0 | | | |
| 0 0 0 0 0 0 | | | | |
| 0 0 0 0 | | | -1,36477 -2,63026 | |
| 0 0 0 0 0 0 | | | | |
| -1,24171 -1,24171 -1,60676 -1,69773 | | | -0,113615 -1,79689 | |
| 5,64452 5,64452 5,60133 6,48973 5,80127 6,67938 | | | | |
| 0,10217 0,10217 | -2,56707 -2,43066 | | 1,07727 | -1,72932 |
| 8,62601 8,62601 9,23169 9,37208 8,5114 8,94537 | | | | |
| 1,01565 1,01565 1,12981 1,68479 1,28964 1,75898 | | | | |
| -2,73338 -2,73338 -4,10133 -1,78652 -2,38009 -3,04337 | | | | |
| -2,02064 -2,02064  -2,64961 -2,64961  -3,33567 -3,33567 | 0 -2,16357 0  0 -3,34088 0  -5,01608 0 -2,12296  0 0 -2,23009 | | | -11,4923 |
|  |  |  |  | -7,98858 |
|  |  |  |  | -4,23819 |
| -8,63987 -8,63987 |  |  |  | -2,6476 |
| -5,58903 -5,58903 | 0 -1,88144 | | -3,71559 | -6,49721 |
| -8,48144 -8,48144 | 0  0 | -4,29289 | -10,3864 | -12,5744 |
| -10,2532 -10,2532 |  | -7,38933 | 0 | -15,0386 |
| -3,64836 -3,64836 -4,55813 | | -4,90373 -3,70153 | | -2,35308 |
| -3,05096 -3,05096 | -1,62654 | -3,43948  -2,58437  -2,07794 | -2,00226 -2,19392 | |
| -1,45802 -1,45802 | 0 |  | 0  0 | -3,92271 |
| -2,9117 -2,9117 -3,55434 | |  |  | -5,61666 |
| -3,44832 -3,44832 | 0 0 | | -1,87651 -1,73864 | |

map00040 map00040 map00040 map00040 map00040 map00040 map00040 map00040 map00040 map00040 map00040 map00040 map00040 map00040 map00040 map00040 map00040 map00040 map00040 map00040 map00040 map00040 map00040 map00040 map00040 map00040 map00040 map00040 map00040 map00040 map00040 map00040

| Dioal.11G080100.1 | 0,696916 | 0,696916 | -2,06116 | -2,82982 | -5,65209 | -4,0569 | map00040 |
| --- | --- | --- | --- | --- | --- | --- | --- |
| Dioal.11G080200.1 | 0 | 0 | 0 | -4,90271 | 0 | 0 | map00040 |
| Dioal.11G080300.1 | 0 | 0 | -1,75127 | 0 | 0 | 0 | map00040 |
| Dioal.11G080400.1 | -5,46179 | -5,46179 | -5,69784 | -4,20231 | -6,30363 | -5,02061 | map00040 |
| Dioal.11G080500.1 | 0 | 0 | 0 | 0 | 0 | 0 | map00040 |
| Dioal.11G081900.1 | -1,70273 | -1,70273 | 0,680271 | -1,28703 | -0,9308 | -1,77809 | map00040 |
| Dioal.11G082000.1 | 1,34904 | 1,34904 | -2,08737 | 1,24692 | -3,44372 | -0,312228 | map00040 |
| Dioal.11G082200.1 | -3,92469 | -3,92469 | -0,611873 | -3,8572 | -3,13169 | -3,74652 | map00040 |
| Dioal.11G082300.1 | 0 | 0 | 0 | -3,41051 | -1,92611 | -5,60765 | map00040 |
| Dioal.11G082400.1 | -2,02019 | -2,02019 | -4,14752 | -2,81764 | -3,14063 | -2,36913 | map00040 |
| Dioal.11G093000.1 | -1,39405 | -1,39405 | -1,74477 | -1,21749 | -1,31595 | -2,27992 | map00040 |
| Dioal.11G103300.1 | 4,35225 | 4,35225 | 3,981 | 4,09759 | 4,27949 | 4,51975 | map00040 |
| Dioal.12G004700.1 | -1,38891 | -1,38891 | -2,38481 | -3,2864 | -4,0874 | -1,68438 | map00040 |
| Dioal.12G006500.1 | 0 0 0 0 0 | | | | | 0 | map00040 |
| Dioal.12G012800.1 | 1,77236 | 1,77236 | 0,241554 | 2,91206 | 1,78234 | 3,17121 | map00040 |
| Dioal.12G014900.1 | 3,21908 | 3,21908 | 2,81495 | 2,91104 | 2,075 | 2,41234 | map00040 |
| Dioal.12G015500.1 | 4,2102 | 4,2102 | 4,56214 | 4,49236 | 5,39139 | 4,79926 | map00040 |
| Dioal.12G025500.1 | 2,95302 | 2,95302 | 3,48279 | 2,85902 | 1,91478 | 3,02906 | map00040 |
| Dioal.12G079400.1 | -0,372983 | -0,372983 | -0,861175 | -1,76126 | -2,97744 | -1,58171 | map00040 |
| Dioal.12G079700.1 | 4,06563 | 4,06563 | 2,74445 | 3,23865 | 2,89495 | 3,40352 | map00040 |
| Dioal.13G014400.1 | -2,39201 | -2,39201 | 1,05692 | -0,114968 | -0,94129 -3,34702 map00040 | | |
| Dioal.13G021200.1 | 0 | 0 | 0 | 0 | 0 | 0 | map00040 |
| Dioal.13G044000.1 | -1,72216 | -1,72216 | 0 | 0 | 0 | -1,57554 | map00040 |
| Dioal.13G045800.1 | 0 | 0 | 0 | -2,08166 | -2,2604 | -2,37778 | map00040 |
| Dioal.13G046000.1 | 0 | 0 | 0 | 0 | 0 | 0 | map00040 |
| Dioal.13G046100.1 | 0 | 0 | 0 | -3,58124 | -2,03994 | -2,36757 | map00040 |
| Dioal.13G053400.1 | -2,45086 | -2,45086 | -3,64495 | -2,14161 | -3,93345 | -0,333886 | map00040 |
| Dioal.13G055900.1 | -1,09606 | -1,09606 | -0,871281 | -1,22831 | -1,14779 | -1,04034 | map00040 |
| Dioal.13G075500.1 | -4,63564 | -4,63564 | 0 | 0 | -6,30427 | -2,31356 | map00040 |
| Dioal.14G015100.1 | -0,220895 | -0,220895 | -0,453222 | 0,471144 | -0,449475 | -0,013866 | map00040 |
| Dioal.14G020300.1 | -2,40971 | -2,40971 | -3,66421 | -4,44368 | -3,51273 | -4,22396 | map00040 |
| Dioal.14G020400.1 | 0 | 0 | 0 | 0 | -2,93102 0 map00040 | | |

Dioal.14G020500.1 Dioal.14G020700.1 Dioal.14G020800.1 Dioal.14G020900.1 Dioal.14G021000.1 Dioal.14G021100.1 Dioal.14G021200.1 Dioal.14G022100.1 Dioal.14G022400.1 Dioal.14G023000.1 Dioal.14G023100.1 Dioal.14G023300.1 Dioal.14G023400.1 Dioal.14G023500.1 Dioal.14G023700.1 Dioal.14G078000.1 Dioal.14G083600.1 Dioal.14G118200.1 Dioal.14G122300.1 Dioal.14G140600.1 Dioal.14G142100.1 Dioal.15G010900.1 Dioal.15G011000.1 Dioal.15G084300.1 Dioal.15G084400.1 Dioal.15G084500.1 Dioal.15G084600.1 Dioal.15G114300.1 Dioal.15G114400.1 Dioal.15G114500.1 Dioal.16G016100.1 Dioal.16G076300.1

| -1,27752 | -1,27752 | -0,221984 | -1,70263 1,24249 | | | -1,72591 |
| --- | --- | --- | --- | --- | --- | --- |
| -1,59942  -0,403668  -2,31303 | -1,59942  -0,403668  -2,31303 | -4,94411 -3,64457 | | | -2,72641 | -4,58448 |
|  |  | -2,36348 -1,86337 | | | -1,11243 | -2,28633 |
|  |  | -3,91944 | | -4,78883 | -1,96067 | -4,46281 |
| -2,80423  -1,02517 | -2,80423  -1,02517 | -5,4603 | | -3,36787 -3,50527 | | -3,78283 |
|  |  | -3,83322 -3,05792 | | | -2,88223 | -3,28408 |
| -2,11627 | -2,11627 | -4,73747  -4,20152 | | 0,546054 | -2,38277 | -1,70794 |
| -2,94724 | -2,94724 |  |  | -0,676583 0,940177 | | -3,00995 |
| 1,78622 | 1,78622 | 1,19784 | 2,90732 1,60837 | | | 2,40791 |
| 0 | 0 | 0  -3,84524  0  0 | 0 -3,1446  -2,19676 2,15564  -2,93249 -2,13846  -2,90341 -2,02073 | | | 0  1,51778  -3,39575  0 |
| -2,11224  -0,960303  -1,47848 | -2,11224  -0,960303  -1,47848 |  |  |  |  |  |
| 0  0 | 0  0 | 0  0 | 0 0  -1,30102 -1,59903 | | | 0  -2,04974 |
| -1,1666 -1,1666 1,43696 1,68387 1,56394  0 0 0 -1,13208 -0,915783 | | | | | | 2,29639  0 |
| 3,85842 | 3,85842 | 3,6144 | 4,32132 4,20273 | | | 3,60079 |
| 0,714465 | 0,714465 | 0,91498 | -0,0012678 | | -2,37915 0,0104093 | |
| 2,89775 | 2,89775 | 2,8156 | 3,07756 4,93023 | | | 3,32294 |
| -0,428156 -0,428156 0,774867 | | | | 0,980697 2,51714 | | 1,62624 |
| -1,79408 | -1,79408 | -2,80174 | -1,84136 | | -4,37252 | -1,03501 |
| -2,48134  -1,49569 | -2,48134  -1,49569 | -0,747876  1,59987 | 0,287349 -0,954873  -0,0932262 0,941664 | | | -0,176182  -1,4191 |
| -4,38065  -4,38065 | -4,38065  -4,38065 | 0  0 | -1,77152 0  0 0 | | | -1,12186  0 |
| 0  -1,4027  -1,81264  -0,924454  0  -1,29071 | 0  -1,4027  -1,81264  -0,924454  0  -1,29071 | 0  0,385537  -1,11758  2,06602  0  0 | 0 0  -1,49345 -0,536253  -4,16256 -1,10627  -1,49615 -1,33182  0 0  -0,3664 -1,43033 | | | 0  -1,81236  -1,66768  -0,358227  0  -0,831393 |

map00040 map00040 map00040 map00040 map00040 map00040 map00040 map00040 map00040 map00040 map00040 map00040 map00040 map00040 map00040 map00040 map00040 map00040 map00040 map00040 map00040 map00040 map00040 map00040 map00040 map00040 map00040 map00040 map00040 map00040 map00040 map00040

| Dioal.16G090700.1 | 0 | 0 | -1,64078 | 0 | 0 | 0 | map00040 |
| --- | --- | --- | --- | --- | --- | --- | --- |
| Dioal.17G001900.1 | -0,534281 | -0,534281 | 0,471115 | -0,828976 | 0,873375 | -0,41824 | map00040 |
| Dioal.17G002000.1 | 0 | 0 | 0 | 0 | 0 | 0 | map00040 |
| Dioal.17G002100.1 | 0 | 0 | 0 | 0 | 0 | 0 | map00040 |
| Dioal.17G007700.1 | -0,06805 | -0,06805 | 0,239255 | 0,329693 -2,00682 -0,754426 map00040 | | | |
| Dioal.17G007800.1 | -3,05356 | -3,05356 | -3,99724 | 0 | -3,36161 | -5,39301 | map00040 |
| Dioal.17G020200.1 | 0 | 0 | 0 | 0 | 0 | -1,67931 | map00040 |
| Dioal.17G048900.1 | 4,00024 4,00024 | | 3,94897 | 4,83705 | 3,84051 | 4,6134 | map00040 |
| Dioal.17G121600.1 | 0,574967 | 0,574967 | -0,0759111 | 1,21778 | 0,413352 | -1,45184 | map00040 |
| Dioal.18G002100.1 | 1,07195 | 1,07195 | -2,45939 | 3,07418 | -2,13727 | 1,43091 | map00040 |
| Dioal.18G002200.1 | 0 | 0 | 0 | -1,97818 | -2,33347 | -2,8264 | map00040 |
| Dioal.18G002700.1 | 0 | 0 | 0 | 0 | 0 | 0 | map00040 |
| Dioal.18G002800.1 | -1,25469 | -1,25469 | -3,86333 | -0,913634 | -1,93317 | -0,726622 | map00040 |
| Dioal.18G002900.1 | 3,28489 | 3,28489 | 1,81608 | 1,74559 | 1,45784 | 2,28136 | map00040 |
| Dioal.18G021200.1 | -3,3848 | -3,3848 | -0,679805 | 0,262477 | 1,15579 | -0,722881 | map00040 |
| Dioal.18G034400.1 | -2,29517 | -2,29517 | -1,4479 | -2,03995 | -4,0939 | -2,34644 | map00040 |
| Dioal.18G035000.1 | 1,60388 | 1,60388 | 1,81285 | 1,31989 | -0,327681 | -3,28704 | map00040 |
| Dioal.18G035700.1 | -0,907479 | -0,907479 | -1,97147 | -0,573369 | 0 | -1,04286 | map00040 |
| Dioal.18G036300.1 | -1,71042 | -1,71042 | 0,11847 | 1,28764 | -2,17699 | 2,36724 | map00040 |
| Dioal.18G040500.1 | -0,769233 | -0,769233 | -0,582044 | -1,18407 | -2,73307 | -0,259983 | map00040 |
| Dioal.18G047000.1 | 0 | 0 | 0 | 0 | 0 | -1,41269 | map00040 |
| Dioal.18G051400.1 | -2,49439 | -2,49439 | 0 | -2,46228 | 1,07114 | -1,49876 | map00040 |
| Dioal.18G051500.1 | 0 | 0 | 0 | 0 | 0 | 0 | map00040 |
| Dioal.18G051600.1 | -5,33592 | -5,33592 | -3,85858 | -1,6763 | -1,49198 | -0,640776 | map00040 |
| Dioal.18G067500.1 | 4,77034 4,77034 | | 4,73631 | 5,18237 | 5,60718 | 5,73145 | map00040 |
| Dioal.18G067600.1 | -1,25103 | -1,25103 | -0,792015 | 1,92946 | -2,89535 | 2,47121 | map00040 |
| Dioal.18G074000.1 | -1,75928 | -1,75928 | -1,8186 | 0 | 0 | 0 | map00040 |
| Dioal.18G093900.1 | 1,36494 | 1,36494 | 1,0919 | 1,35799 | 1,16289 | 1,54461 | map00040 |
| Dioal.19G014900.1 | 2,87282 | 2,87282 | 2,71935 | 3,14694 | 2,74852 | 3,35094 | map00040 |
| Dioal.19G022000.1 | 3,5532 | 3,5532 | 3,39005 | 3,32989 | 3,01591 | 3,61818 | map00040 |
| Dioal.19G045900.1 | 7,1389 | 7,1389 | 5,17488 | 8,68754 | 6,76732 | 6,66795 | map00040 |
| Dioal.19G078300.1 | 1,99015 | 1,99015 | 3,42317 | 3,52501 | 3,35641 | 3,36384 | map00040 |

Dioal.19G105500.1 Dioal.19G105600.1 Dioal.19G134100.1 Dioal.19G140100.1 Dioal.19G149200.1 Dioal.19G149600.1 Dioal.19G149900.1 Dioal.19G150200.1 Dioal.19G150300.1 Dioal.19G150500.1 Dioal.19G150600.1 Dioal.19G150700.1 Dioal.19G181200.1 Dioal.19G187400.1 Dioal.20G018900.1 Dioal.20G033700.1 Dioal.20G035100.1 Dioal.20G035200.1 Dioal.20G035900.1 Dioal.20G042200.1 Dioal.20G055000.1 Dioal.20G055100.1 Dioal.20G055200.1 Dioal.20G055300.1 Dioal.20G055400.1 Dioal.20G055500.1 Dioal.20G055700.1 Dioal.20G055800.1 Dioal.20G055900.1 Dioal.20G056000.1 Dioal.20G056100.1 Dioal.20G056200.1

| -2,28373 | -2,28373 | 0 | -1,96055 | -0,21611  -1,63592 | -1,89475 |
| --- | --- | --- | --- | --- | --- |
| 1,29431 | 1,29431 | -2,27012 | -0,0739693 |  | 0,287859 |
| -2,38148 | -2,38148 | -1,58854 | -2,36874 -3,20019 | | -3,90364 |
| 0,897574 | 0,897574 | 0,154265 2,22868 | | 1,01091 | 1,70566 |
| 4,72601 | 4,72601 | 2,6774 5,49452 | | 4,07627 | 5,7335 |
| 0 | 0 | -1,58842 -1,559 | | -3,50485 | -1,85045 |
| 2,32366 | 2,32366 | 3,15646 2,79221 | | 2,95871 | 2,90705 |
| 2,60531 | 2,60531 | 1,72566 2,55952 | | 2,21421 | 2,40139 |
| 2,46373 | 2,46373 | 1,68252 2,28239 | | 2,00499 | 1,96732 |
| -0,565452 | -0,565452 | 1,10422 0,453482 | | -2,81325 | -0,950098 |
| 0,387805 | 0,387805 | -1,66756 0,762596 | | -2,07357 | 0,106548 |
| -1,03952 | -1,03952 | -1,83745 1,27217 | | -2,23168 | -0,546021 |
| -3,54332 | -3,54332 | -2,20513 -1,93219 | | -2,71744 | -1,19147 |
| -0,793551 | -0,793551 | -1,08321 0,0414146 | | -0,476839 | 0,316195 |
| -1,36846 | -1,36846 | -1,37913 -0,571863 | | 0 | 0,369727 |
| 0,256086 | 0,256086 | 0,680751 0,903116 | | 0,458139 | 0,381457 |
| 0 | 0 | 0 0 | | 0 | 0 |
| 0 | 0 | 0 0 | | 0 | 0 |
| -3,70676 | -3,70676 | 0 -0,325965 | | -4,59699 | -5,80307 |
| -3,30644 | -3,30644 | 0 -1,20336 | | -1,01246 | -2,88996 |
| -3,3078 | -3,3078 | 0 -4,02716 | | -3,40652 | -1,76263 |
| -2,83351 | -2,83351 | -3,19546 -1,84705 | | -2,66054 | -3,99256 |
| -2,80359 | -2,80359 | 0 -1,34762 | | -1,32079 | -1,82691 |
| 0 | 0 | -0,997623 -1,80689 | | -2,32404 | -2,36545 |
| -1,27907 | -1,27907 | -2,17534 -1,10984 | | -4,80219 | -0,64127 |
| -2,01571 | -2,01571 | 0 -2,04994 | | -1,54541 | 0 |
| -2,8141 | -2,8141 | -1,32073 -3,5657 | | 0 | -3,05278 |
| 2,10026 | 2,10026 | 1,72258 1,66564 | | 1,86551 | 1,74113 |
| -2,44527 | -2,44527 | -2,77302 0,856753 | | -3,48053 | -2,97161 |
| 0 | 0 | 0 -0,847647 | | -1,7969 | -3,20361 |
| -1,20988 | -1,20988 | -2,23847 | 2,07366 | -1,24052 | -1,4676 |
| 2,44639 | 2,44639 | 3,06757 | 2,19406 3,4726 | | 2,04295 |

map00040 map00040 map00040 map00040 map00040 map00040 map00040 map00040 map00040 map00040 map00040 map00040 map00040 map00040 map00040 map00040 map00040 map00040 map00040 map00040 map00040 map00040 map00040 map00040 map00040 map00040 map00040 map00040 map00040 map00040 map00040 map00040

| Dioal.20G056300.1 | 3,47592 | 3,47592 | 2,70377 | 4,40085 | 4,00352 | 3,37793 | map00040 |
| --- | --- | --- | --- | --- | --- | --- | --- |
| Dioal.20G056400.1 | 3,12161 | 3,12161 | 2,80571 | 4,49744 | 3,07491 | 3,36033 | map00040 |
| Dioal.20G103100.1 | 1,6454 | 1,6454 | 1,99552 | 2,32361 | 2,22739 | 1,73352 | map00040 |
| Dioal.01G018700.1 | 3,62311 | 3,25206 | 3,02819 | 3,33157 | 2,67139 | 3,1238 | map00500 |
| Dioal.01G070900.1 | 3,14145 | 2,45587 | 2,35454 | 2,81313 | 1,88463 | 3,4304 | map00500 |
| Dioal.02G090200.1 | -3,03111 | -1,66288 | -2,53788 | -2,24869 | -2,89061 | -2,19826 | map00500 |
| Dioal.02G098800.1 | 6,63014 | 6,64583 | 7,2633 | 6,58206 | 6,16077 | 6,63116 | map00500 |
| Dioal.03G018100.1 | 1,70488 | 1,68313 | 1,89436 | 2,33768 | 2,48645 | 2,17667 | map00500 |
| Dioal.03G020000.1 | 3,62269 | 3,82541 | 4,20026 | 3,90428 | 3,57437 | 3,84405 | map00500 |
| Dioal.03G027600.1 | 1,28882 | 1,8332 | -0,407658 | 2,50527 | 1,05871 | 2,79144 | map00500 |
| Dioal.03G073600.1 | 6,2811 | 7,26974 | 7,17964 | 7,05915 | 7,70113 | 7,13528 | map00500 |
| Dioal.04G001400.1 | 6,32726 | 6,8642 | 6,59346 | 7,09753 | 7,08253 | 6,78208 | map00500 |
| Dioal.04G013300.1 | 0,754462 | 1,85932 | 0,908923 | 2,10182 | 4,46699 | 3,2303 | map00500 |
| Dioal.04G020700.1 | 0,657232 | -2,9661 | 0,489591 | 0 | -1,46955 | -2,58356 | map00500 |
| Dioal.04G021700.1 | 0 | 0 | 0 | 0 | 0 | 0 | map00500 |
| Dioal.04G091400.1 | 0,412366 | 1,53487 | 0,141836 | 0,854711 | -0,197501 | -0,0423316 | map00500 |
| Dioal.04G092600.1 | 0 | -4,30115 | 0 | 0 | -3,01436 | -2,74528 | map00500 |
| Dioal.04G095100.1 | 3,3845 | 3,10193 | 2,53568 | 3,28546 | 2,50069 | 2,3905 | map00500 |
| Dioal.04G101300.1 | 2,39898 | 0,191701 | 0,265367 | 0,788439 | 0,992421 | 2,08491 | map00500 |
| Dioal.04G130300.1 | 0 | 0 | 0 | 0 | 0 | 0 | map00500 |
| Dioal.04G158700.1 | 6,30945 | 6,25891 | 6,14656 | 6,63332 | 5,69651 | 6,2643 | map00500 |
| Dioal.04G168200.1 | 4,18173 | 2,60061 | 4,50162 | 4,24547 | 4,48214 | 2,62708 | map00500 |
| Dioal.04G184200.1 | -1,04638 | -0,77944 | -1,32567 | -1,05367 | -1,52684 | -2,55693 | map00500 |
| Dioal.05G025500.1 | 0 | 0 | 0 | -1,70831 | -1,5141 | -3,46409 | map00500 |
| Dioal.05G053000.1 | 0 | 0 | 0 | 0 | 0 | 0 | map00500 |
| Dioal.05G053400.1 | 0 | 0 | 0 | 0 | -1,67243 | 0 | map00500 |
| Dioal.05G055600.1 | 0,276297 | 0,890374 | 0,515794 | -0,518578 | -0,0841507 | -0,218867 | map00500 |
| Dioal.05G075200.1 | 0 | 0 | -2,43554 | 0 | 0 | 0 | map00500 |
| Dioal.05G075500.1 | 0 | -1,71311 | 0 | 0 | 0 | 0 | map00500 |
| Dioal.05G075600.1 | 2,21915 | 2,24143 | 2,30556 | 3,07657 | 2,5998 | 2,79844 | map00500 |
| Dioal.05G096100.1 | 5,13048 | 4,9467 | 5,16839 | 5,4498 | 5,19749 | 5,53187 | map00500 |
| Dioal.05G113700.1 | -0,473007 | -0,40209 | 1,29921 | -0,698515 | 2,13182 | 0,545201 | map00500 |

| Dioal.05G114400.1 | 0 | 0 | 0 | -3,00641 | -1,40115 | 0 | map00500 |
| --- | --- | --- | --- | --- | --- | --- | --- |
| Dioal.05G128700.1 | 1,87536 | -1,07306 | 1,88153 | -0,946438 | -3,026 | -0,109279 | map00500 |
| Dioal.05G145300.1 | 4,76096 | 4,33735 | 4,47751 | 4,96304 | 4,59974 | 5,1305 | map00500 |
| Dioal.05G175900.1 | -2,93996 | -4,68667 | -4,25368 | -3,17709 | -3,88564 | -3,25584 | map00500 |
| Dioal.05G185800.1 | 6,18618 | 6,72062 | 6,46912 | 7,24284 | 6,54867 | 6,9359 | map00500 |
| Dioal.05G188500.1 | 2,48721 | 2,24145 | 2,25032 | 3,8873 | 3,29863 | 3,90726 | map00500 |
| Dioal.05G189200.1 | 0,392296 | 1,04605 | 0,63319 | 0,346908 | 1,69387 | 0,40851 | map00500 |
| Dioal.05G210400.1 | -1,18378 | -0,0971987 | -1,16032 | -0,866052 | -0,891277 | -0,433315 | map00500 |
| Dioal.05G211400.1 | -1,18378 | -0,0971987 | -1,16032 | -0,866052 | -0,891277 | -0,433315 | map00500 |
| Dioal.06G018300.1 | 4,3193 | 4,64873 | 4,16109 | 5,02553 | 5,26604 | 5,24371 | map00500 |
| Dioal.06G019000.1 | -1,3819 | 5,22613 | -1,15199 | 4,74106 | -2,4887 | 4,73241 | map00500 |
| Dioal.06G026700.1 | 0 | 3,55445 | 0,299547 | 3,93144 | 0,413765 | 3,91445 | map00500 |
| Dioal.06G027400.1 | 0 | -1,54071 | 0,145563 | 1,92051 | -1,50333 | 2,13037 | map00500 |
| Dioal.06G028600.1 | -1,20002 | 1,35062 | 0 | 1,77309 | 0 | 0,836062 | map00500 |
| Dioal.06G028900.1 | 0,895115 | 2,03038 | 0 | 2,4135 | 0,13399 | 0,378007 | map00500 |
| Dioal.06G029200.1 | 0 | 1,02028 | 0 | 0,792572 | 0 | 1,13514 | map00500 |
| Dioal.06G033200.1 | 2,75082 | 2,28036 | 2,3593 | 1,59029 | 1,93682 | 1,65127 | map00500 |
| Dioal.06G045200.1 | 8,63661 | 7,32122 | 8,09907 | 9,06119 | 7,73956 | 8,06084 | map00500 |
| Dioal.06G045800.1 | -0,011211 | -0,256987 | 0,147578 | 0,555405 | 2,69109 | -0,629946 | map00500 |
| Dioal.06G055800.1 | 3,66986 | 3,21647 | 3,87658 | 3,88789 | 3,46819 | 3,83264 | map00500 |
| Dioal.06G067700.1 | 0 | 0 | 0 | 0 | 0 | 0 | map00500 |
| Dioal.06G072700.1 | 1,41611 | 2,35306 | 1,86994 | 1,58115 | 1,64317 | 1,60719 | map00500 |
| Dioal.06G081700.1 | -1,86065 | -1,44989 | 0 | -2,63174 | -2,64487 | 0 | map00500 |
| Dioal.06G084000.1 | 3,12085 | 3,20279 | 3,58832 | 2,98928 | 3,06386 | 2,96295 | map00500 |
| Dioal.07G000900.1 | -1,16836 | -0,528039 | -2,05814 | -0,382133 | 0,514017 | -0,150662 | map00500 |
| Dioal.07G026700.1 | -0,804007 | 0 | 0 | 0 | -1,69453 | 0 | map00500 |
| Dioal.07G051400.1 | 4,0786 | 4,56483 | 4,18226 5,23215 | | 3,86654 5,34162 map00500 | | |
| Dioal.07G064700.1 | 3,03811 | 3,35457 | 3,43229 | 4,0348 | 3,01449 | 3,80372 | map00500 |
| Dioal.07G067100.1 | 6,80986 | 5,7646 | 7,01128 | 7,22393 | 6,96463 | 6,68731 | map00500 |
| Dioal.07G095500.1 | -0,640191 | -2,13405 | -0,629757 | -0,297439 | -2,64048 | -1,12782 | map00500 |
| Dioal.08G008000.1 | -0,0583738 | -2,85902 | -0,081364 | -2,04383 | 0,743534 | -0,451401 | map00500 |
| Dioal.08G030400.1 | -1,66471 | 0 | -1,59195 | -1,77287 | -0,749797 | 0 | map00500 |

Dioal.08G041200.1 Dioal.08G081500.1 Dioal.08G088800.1 Dioal.08G089400.1 Dioal.08G106000.1 Dioal.08G124800.1 Dioal.08G130500.1 Dioal.09G032200.1 Dioal.09G085500.1 Dioal.10G014800.1 Dioal.10G054800.1 Dioal.11G000200.1 Dioal.11G003300.1 Dioal.11G015700.1 Dioal.11G019100.1 Dioal.11G027600.1 Dioal.11G039500.1 Dioal.11G051100.1 Dioal.11G072300.1 Dioal.11G072500.1 Dioal.12G022300.1 Dioal.12G034900.1 Dioal.12G044100.1 Dioal.12G056900.1 Dioal.12G069200.1 Dioal.12G069300.1 Dioal.12G094700.1 Dioal.13G021600.1 Dioal.13G021700.1 Dioal.13G082100.1 Dioal.13G093000.1 Dioal.13G093100.1

| 4,67527 | 4,82654 | 4,62713 | 6,06765 | 5,1468 | 5,67096 |
| --- | --- | --- | --- | --- | --- |
| -4,44614 | -2,68126 | -2,27007 | -4,52441 | 0,924255 | 0 |
| 4,79836 | 6,34102 | 7,04125 | 6,3594 | 5,07782 | 5,95646 |
| 2,3398 | 2,7859 | 2,70643 | 2,97408 | 2,6457 | 2,84914 |
| -4,19005 | -1,55388 | -2,14293 | -1,14764 | 0 | -0,47737 |
| -3,26653 | -3,44464 | -3,19027 | -3,11989 | -3,0394 | -4,62139 |
| 5,15733 | 5,68138 | 5,26032 | 6,57294 | 5,92401 | 6,22561 |
| 4,13996 | 3,57829 | 3,71834 | 4,91587 | 4,70157 | 5,059 |
| 8,02229 | 7,90349 | 8,33705 | 8,13503 | 7,42055 | 6,82756 |
| 4,44571 | 3,46865 | 3,42547 | 2,25062 | 0  0,0675413 | 2,15527 |
| -1,641 | 0 | -1,57831 | -2,1941 |  | -2,54627 |
| 3,27094 | 3,86069 | 2,80214 | 3,91749 | 3,66774 | 4,08116 |
| 5,68043 | 6,43673 | 6,73853 | 6,91903 | 7,70452 | 7,47843 |
| 0 | 0 | 0 | 0 | 0 | 0 |
| 3,19186  3,91052  3,5921  4,63507  5,18857 | 3,81042  3,68278  3,70174  4,49976  4,911 | 4,12092  3,67989  3,18518  4,46253  4,90323 | 3,39101  4,25438  3,88692  5,50664  5,50924 | 2,67223  3,43466  3,31531  4,70423  4,7998 | 3,2806  3,88089  3,57534  5,1671  5,11243 |
| 9,01363 | 8,62601 | 9,23169 | 9,37208 | 8,5114 | 8,94537 |
| 7,7122 | 6,21158 | 7,18578 | 7,73073 | 5,97911 | 7,26016 |
| 0 | 0 | 0 | 0 | 0 | 0 |
| 1,18138 | 1,28256 | 1,13088 | 2,06339 | 2,00649 | 1,00857 |
| 5,31182 | 5,24647 | 4,57011 | 5,63934 | 4,47657 | 5,34966 |
| 0  0 | 0  0 | 0  0 | 0  0 | 0  0 | 0  0 |
| 3,78957 | 4,14706 | 5,00994 | 4,4152 | 4,12514 | 4,13287 |
| 8,94418  9,83467 | 8,50974  9,51763 | 8,93406  9,99517 | 8,42153  9,36977 | 6,76905 | 7,13531 |
|  |  |  |  | 7,81109 | 8,28664 |
| 4,09511 | 2,8588 | 3,22846 | 6,15412 | 4,59493 | 5,97936 |
| 0  0 | 0  0 | 0  0 | 0  0 | 0  0 | 0  0 |

map00500 map00500 map00500 map00500 map00500 map00500 map00500 map00500 map00500 map00500 map00500 map00500 map00500 map00500 map00500 map00500 map00500 map00500 map00500 map00500 map00500 map00500 map00500 map00500 map00500 map00500 map00500 map00500 map00500 map00500 map00500 map00500

| Dioal.13G093500.1 | 1,59229 | -1,7189 | 1,40039 | 0,581377 | 0,521348 | -0,225633 | map00500 |
| --- | --- | --- | --- | --- | --- | --- | --- |
| Dioal.13G093600.1 | 0 | -3,39354 | -5,2671 | 0 | 0 | -1,61742 | map00500 |
| Dioal.14G009900.1 | 0,0072207 | 1,03032 | 2,96666 | 1,56074 | 2,44101 | 0,677013 | map00500 |
| Dioal.14G031600.1 | -4,75855 | -2,69883 | -1,94319 | 0,622272 | -2,46026 | -1,08897 | map00500 |
| Dioal.14G047200.1 | 8,03729 | 8,32324 | 8,12208 | 8,26307 | 7,15999 | 7,3596 | map00500 |
| Dioal.14G047300.1 | 3,97182 | 4,12106 | 3,80402 | 4,24807 | 3,50848 | 3,85895 | map00500 |
| Dioal.14G066900.1 | 4,46565 | 3,27798 | 3,65605 | 4,65963 | 3,66246 | 4,14118 | map00500 |
| Dioal.14G081700.1 | 5,3013 | 4,41058 | 3,40368 | 4,74016 | 5,75077 | 4,86021 | map00500 |
| Dioal.14G127500.1 | 5,13177 | 4,84744 | 4,84387 | 5,37864 | 4,45975 | 4,94779 | map00500 |
| Dioal.14G143700.1 | 3,62553 | 3,05721 | 2,96554 | 4,31722 | 3,40808 | 4,54729 | map00500 |
| Dioal.15G006700.1 | 1,98798 | 2,33788 | 2,57767 | 2,32998 | 2,60811 | 1,70765 | map00500 |
| Dioal.15G034100.1 | -2,81847 | -3,7002 | -1,39324 | -2,7177 | -2,03841 | -1,21763 | map00500 |
| Dioal.15G046000.1 | 1,65896 | 1,82294 | 1,90354 | 2,0899 | 2,50576 | 2,23403 | map00500 |
| Dioal.15G075400.1 | 0 | -2,77475 | 0 | -1,75681 | -2,09129 | -1,91587 | map00500 |
| Dioal.15G084200.1 | 4,74117 | 5,27026 | 3,87634 | 4,44474 | 2,23601 | 3,80103 | map00500 |
| Dioal.15G119500.1 | 2,11589 | 1,89257 | 2,62075 | -0,345737 | 0,379427 | 0,790191 | map00500 |
| Dioal.16G010200.1 | 2,04973 | 2,75933 | 2,97533 | 3,67705 | 2,55601 | 3,65154 | map00500 |
| Dioal.16G034800.1 | 4,20899 | 5,54828 | 3,52203 | 5,92877 | 4,50827 | 4,48321 | map00500 |
| Dioal.16G046000.1 | 7,33749 | 8,25465 | 7,75392 | 8,00628 | 6,9863 7,82543 map00500 | | |

| Dioal.17G003400.1 | 6,533 | 5,70855 | 5,99146 | 6,70063 | 6,2372 | 6,21247 | map00500 |
| --- | --- | --- | --- | --- | --- | --- | --- |
| Dioal.17G025500.1 | -0,185228 | -1,73285 | -0,716435 | -1,73647 | -0,009012 | 0,253289 | map00500 |
| Dioal.17G051800.1 | 1,23371 | 1,29705 | 1,00542 | 1,00807 | 0,106619 | 0,667389 | map00500 |
| Dioal.17G059600.1 | 0,906244 | 0,593738 | 0,683656 | 1,65899 | 1,51031 | 1,35851 | map00500 |
| Dioal.17G063000.1 | -2,79411 | -1,91618 | -3,32109 | -2,26282 | -2,23005 | -1,7242 | map00500 |
| Dioal.17G063100.1 | 0,308527 | 0,529762 | -0,192624 0,886516 | | -0,0369037 1,13521 map00500 | | |
| Dioal.17G069300.1 | -1,91593 | -3,24461 | -1,7712 | -1,48324 | 0,36349 | 0,0117233 | map00500 |
| Dioal.17G084700.1 | 0 | 0 | 0 | 0 | 0 | 0 | map00500 |
| Dioal.17G112400.1 | -5,0119 | -4,46127 | -3,52801 | 0 | -1,9458 | -2,93646 | map00500 |
| Dioal.17G115600.1 | 2,07732 | 2,61956 | -1,74684 | -1,82965 | -0,115129 | -1,9597 | map00500 |
| Dioal.18G009100.1 | 0 | 0 | 0 | 0 | 0 | 0 | map00500 |
| Dioal.18G014200.1 | 5,37354 | 5,47219 | 5,63506 | 6,06126 | 5,98778 | 6,03801 | map00500 |
| Dioal.18G019500.1 | 5,70981 | 6,11148 | 6,74409 | 5,8438 | 6,13481 | 5,61948 | map00500 |

| Dioal.18G032200.1 | 0 | 0 | 0 | 0 | 0 | 0 | map00500 |
| --- | --- | --- | --- | --- | --- | --- | --- |
| Dioal.18G053800.1 | 4,7299 | 4,17332 | 4,52688 | 4,20014 | 3,9755 | 4,50101 | map00500 |
| Dioal.18G063800.1 | -2,40697 | -0,970901 | -2,33442 | -1,57983 | -2,44363 | -3,48021 | map00500 |
| Dioal.18G067300.1 | 5,25675 | 5,36795 | 5,36869 | 5,46291 | 4,66897 | 5,10948 | map00500 |
| Dioal.18G070300.1 | 6,03318 | 6,18985 | 7,60899 | 6,30452 | 7,38801 | 7,71498 | map00500 |
| Dioal.18G093900.1 | 1,74998 | 1,36494 | 1,0919 | 1,35799 | 1,16289 | 1,54461 | map00500 |
| Dioal.18G106400.1 | 0 | 0 | 0 | 0 | 0 | -0,95623 | map00500 |
| Dioal.18G118300.1 | 2,87449 | 3,27282 | 2,91044 | 4,02566 | 3,03279 | 3,67968 | map00500 |
| Dioal.18G119600.1 | 2,72609 | 2,78016 | 2,75483 | 2,47915 | 1,81142 | 2,1719 | map00500 |
| Dioal.19G036500.1 | 0 | 0 | 0 | -1,9873 | -1,70182 | -2,77045 | map00500 |
| Dioal.19G065400.1 | -1,30078 | 0 | -2,88232 | 0 | 0 | -1,95192 | map00500 |
| Dioal.19G065500.1 | -4,31247 | -4,98986 | -2,85481 | -2,26414 | -2,84389 | -1,25374 | map00500 |
| Dioal.19G091000.1 | 4,71853 | 4,45896 | 4,50862 | 4,95245 | 4,32986 | 4,62709 | map00500 |
| Dioal.19G149200.1 | 4,46333 | 4,72601 | 2,6774 | 5,49452 | 4,07627 | 5,7335 | map00500 |
| Dioal.19G165600.1 | -2,57577 | -3,83325 | -2,55859 | 0 | -0,546302 | -2,50862 | map00500 |
| Dioal.20G036800.1 | 6,65998 | 6,97388 | 7,11211 | 6,87587 | 6,22907 | 5,80118 | map00500 |
| Dioal.20G037500.1 | 1,32356 | 1,23503 | 1,94878 | 1,07816 | 2,60551 | 2,08514 | map00500 |
| Dioal.20G041900.1 | 6,7153 | 6,39087 | 6,62342 | 6,71861 | 5,96481 | 6,35051 | map00500 |
| Dioal.20G057000.1 | 2,98957 | 2,06332 | 2,22329 | 3,39561 | 2,57132 | 2,6145 | map00500 |
| Dioal.20G083600.1 | 3,03831 | 2,99824 | 3,18013 | 3,96799 | 2,68413 | 3,31393 | map00500 |
| Dioal.20G083900.1 | -2,77171 -1,3014 -0,908516 | | | 0,142035 | -0,718117 | -0,0890005 | map00500 |

| Dioal.20G091400.1 | -0,525567 | -2,91574 | 0,2971 | -0,161438 | -0,307218 | -0,555493 | map00500 |
| --- | --- | --- | --- | --- | --- | --- | --- |
| Dioal.20G091900.1 | 6,11817 | 4,88394 | 5,2847 | 6,60025 | 5,86287 | 5,13515 | map00500 |
| Dioal.01G016100.1 | 0 | 0 | -1,4011 | -1,40894 | -0,907913 | -1,4499 | map00941 |
| Dioal.01G016200.1 | 1,63886 | 0,0661003 | -1,04352 | -2,49786 | -1,4471 | -1,03138 | map00941 |
| Dioal.02G010500.1 | 0 | 0 | 0 | 0 | 0 | 0 | map00941 |
| Dioal.02G010900.1 | 0 | -1,64535 | 0 | 0 | 0 | 0 | map00941 |

| Dioal.02G011000.1 | 0 -0,415747 | | 0,343219 -2,0809 -2,38982 | | | -1,69596 | map00941 |
| --- | --- | --- | --- | --- | --- | --- | --- |
| Dioal.02G026600.1 | -0,615987 -0,850937 | | 0,233839 -0,834963 0,699591 | | | -0,966182 | map00941 |
| Dioal.02G073700.1 | 6,04083 | 6,31205 | 7,11278 | 5,96116 | 6,96776 | 5,90139 | map00941 |
| Dioal.03G018700.1 | 0 | 0,418959 | 0 | 0,907865 | -1,45781 | -0,463658 | map00941 |
| Dioal.03G018800.1 | 1,68651 | 1,0176 | 1,99065 | -0,940157 | 0,583375 | -0,0650182 | map00941 |

Dioal.03G018900.1 Dioal.03G019000.1 Dioal.03G024200.1 Dioal.03G031200.1 Dioal.03G031300.1 Dioal.03G032100.1 Dioal.03G035900.1 Dioal.03G036000.1 Dioal.03G036100.1 Dioal.03G036200.1 Dioal.03G036300.1 Dioal.03G036400.1 Dioal.03G042900.1 Dioal.03G043300.1 Dioal.03G043400.1 Dioal.03G043600.1 Dioal.04G073400.1 Dioal.04G075100.1 Dioal.04G171800.1 Dioal.04G172600.1 Dioal.05G128000.1 Dioal.05G139300.1 Dioal.05G141100.1 Dioal.05G149100.1 Dioal.05G163700.1 Dioal.05G163800.1 Dioal.05G163900.1 Dioal.05G164000.1 Dioal.05G164100.1 Dioal.07G112500.1 Dioal.08G035700.1 Dioal.08G040100.1

| -1,75101 1,78545 -0,901992 -0,330505 -0,736628 0,880074 | | | | | |
| --- | --- | --- | --- | --- | --- |
| 0 -1,35046 0 | | | -2,9782 | 0 0 | |
| 1,95231 1,19872 1,18071 1,68696 | | | | 3,27554 | 4,4285 |
| 0 -4,15017 0 0 0 0 | | | | | |
| -0,7175 | 0  2,22921 | -0,928933  -0,7829 | 0 0 0 | | |
| -2,10372 |  |  | -2,43166 -2,38397 | | -3,53806 |
| -3,06901 | -1,29887 | -4,2847 | -1,45491 | -0,529964 | -1,86303 |
| 0,554167 1,41326 -0,152608 0 -1,57572 0 | | | | | |
| 0,955059  -2,24383 | -3,71827  -3,67466 | -1,5342  -2,0454 | -0,0935463 0 | | -1,79479 |
|  |  |  | 0 0 0 | | |
| 0,241613  1,08766 | 1,0609 | -1,6013 -1,39525 | | 1,62002 | -1,80189 |
|  | 1,89927 1,31274 0,932525 0,931302 0,184787 | | | | |
| 0 2,15506 0 0 0 0 | | | | | |
| 0 0 -1,40389 0 0  -2,86831 -1,71992 -0,878203 -1,08075 -1,87586 | | | | | 0  0,648383 |
| 0,291774 -1,15232 | | 1,34862 1,30128 | | 0,0278163 | 2,12064 |
| -1,9709 -1,89324 -1,53591  -3,88287 -1,57442 -3,00648 | | | -0,453135  -0,6606 | -3,53864 | -2,82184 |
|  |  |  |  | 0,0270347 | -4,44202 |
| 3,42715 2,87781 2,95623 3,65823 2,97101 3,21624 | | | | | |
| -1,89777 -1,79485 -2,59418 -1,91584 -2,25654 -1,75696 | | | | | |
| 1,11851 0,439853 | | -1,04542 | 0,183832 1,09345 | | -0,272195 |
| -0,264558 | -1,30186 -1,6515 -1,58122 | | | 1,98845 | -3,70865 |
| -0,168701 -0,230207 | | 0,565203 | 2,28168 1,45921 | | 0,403339 |
| 1,09348 | -1,9591 | -0,982073 | 0,628015 | -1,29085 -1,29861 | |
| 3,3071 2,72066 | | 2,92448 | 4,213 | 7,16428 | 4,07359 |
| 3,13729 | 0,5198 | 5,3004 | 3,70006 3,10629 | | 2,89049 |
| -0,848783 -0,909133 | | -1,97109 | -0,62158 0,620733 -0,522698 | | |
| 0 0 0 0 | | | | -1,92749 -1,49172 | |
| 0 0 | | -1,25074 -0,889859 | | 0 | -0,935483 |
| -2,95845 | -1,86213 -2,30815  -1,72224 -2,0455 | | -0,742535 | 1,60418 | -1,65333 |
| 0,900857 |  |  | 0,010397 | -1,52299 -1,31328 | |
| 5,9626 | 3,38173 3,56846 4,15891 | | | 0,289537 | 2,19287 |

map00941 map00941 map00941 map00941 map00941 map00941 map00941 map00941 map00941 map00941 map00941 map00941 map00941 map00941 map00941 map00941 map00941 map00941 map00941 map00941 map00941 map00941 map00941 map00941 map00941 map00941 map00941 map00941 map00941 map00941 map00941 map00941

| Dioal.08G085300.1 | 5,42971 | 3,58776 | 5,61374 | 5,38163 | 7,62495 | 3,93718 | map00941 |
| --- | --- | --- | --- | --- | --- | --- | --- |
| Dioal.08G106200.1 | 3,50306 | 2,85837 | 3,75075 | 2,88663 | -0,789583 | 1,72405 | map00941 |
| Dioal.08G106400.1 | -1,68398 | 0 | 0 | 0 | -2,90507 | -1,68207 | map00941 |
| Dioal.08G106500.1 | -0,330525 | 0,378883 | -0,491847 | 0,105448 | -0,85434 | -0,859193 | map00941 |
| Dioal.08G106600.1 | -1,60184 | -1,44616 | -2,64637 | -2,95801 | -2,07943 | -6,48015 | map00941 |
| Dioal.08G106800.1 | -2,1115 | -1,34817 | -1,33899 | -0,943797 | -1,37275 | -3,09256 | map00941 |
| Dioal.08G106900.1 | -4,42483 | -0,94995 | -2,97948 | -2,11891 | -0,95832 | -3,62616 | map00941 |
| Dioal.08G107000.1 | 5,24956 | 5,18579 | 4,98449 | 5,10045 | 3,95271 | 4,2951 | map00941 |
| Dioal.08G107100.1 | 5,00706 | 2,83806 | 5,11231 | 3,99698 | 3,90031 | 2,8085 | map00941 |
| Dioal.08G131200.1 | -1,48755 | -2,83025 | -1,34714 | -1,49128 | -1,22754 | -1,30254 | map00941 |
| Dioal.09G035500.1 | 0 | 0 | -1,78264 | 0 | 0 | 0 | map00941 |
| Dioal.09G035600.1 | -1,20419 | 0 | -1,57177 | -0,935487 | 0 | -1,39006 | map00941 |
| Dioal.09G071500.1 | 3,24489 | 2,30297 | 4,91796 | 4,2459 | 7,1791 | 5,87319 | map00941 |
| Dioal.10G004500.1 | 4,04127 | 3,53061 | 4,72567 | 5,73421 | 8,32549 | 6,02138 | map00941 |
| Dioal.10G018400.1 | -1,35842 | -0,697023 | -1,27889 | -2,34457 | 0 | 0 | map00941 |
| Dioal.10G018600.1 | 0,245649 | 0,294095 | 1,62039 0,162507 -0,106224 | | | -0,0040397 | map00941 |

| Dioal.10G018700.1 | 1,89662 | 2,56725 | -3,25024 | 4,18493 | 3,11395 | 4,32874 | map00941 |
| --- | --- | --- | --- | --- | --- | --- | --- |
| Dioal.10G018800.1 | 5,57829 | 5,94045 | 5,86652 | 6,73893 | 5,60587 | 6,81977 | map00941 |
| Dioal.10G037900.1 | -1,44647 | -1,89233 | -0,789055 | -0,480403 | -1,51007 | -2,18731 | map00941 |
| Dioal.11G005600.1 | 3,72632 | 3,27822 | 3,64804 | 4,2186 | 3,72698 | 4,41814 | map00941 |
| Dioal.11G008200.1 | -1,33645 | 0 | 0 | -2,52888 | 0,0744537 | -2,51065 | map00941 |
| Dioal.11G008300.1 | 4,78886 | 2,78931 | 4,46156 | 4,52805 | 5,89211 | 3,50767 | map00941 |
| Dioal.11G036600.1 | 0 | -1,56966 | 0 | -2,86638 | -1,29338 | 0 | map00941 |
| Dioal.11G053100.1 | 4,02315 | 3,29878 | 4,13694 | 5,01497 | 7,56519 | 6,30665 | map00941 |
| Dioal.11G084800.1 | 0,689848 | 0,85159 | 0,84559 | 3,2703 | 1,55639 | 2,51918 | map00941 |
| Dioal.11G089300.1 | -0,0711363 | 1,6273 | 2,46549 | 2,41593 | 7,01341 | 2,30669 | map00941 |
| Dioal.12G070300.1 | 0 | -1,44335 | 0 | 0 | -1,63938 | -1,51076 | map00941 |
| Dioal.13G078400.1 | 0 | 0 | 0 | 0 | 0 | -1,21314 | map00941 |
| Dioal.14G034200.1 | 0 | 0 | 0 | 0 | 0 | 0 | map00941 |
| Dioal.14G059300.1 | 0 | -3,52597 | 0 | 0,678023 | -2,03911 | 0,272596 | map00941 |
| Dioal.14G059400.1 | 3,72175 | 3,01861 | 0,965834 | 3,72613 | 3,95804 | 3,6276 | map00941 |
| Dioal.14G121900.1 | 1,46055 | 1,83373 | 0,825758 | 3,15178 | 1,3954 | -0,0336703 | map00941 |

| Dioal.15G024300.1 | 2,02901 | 3,41305 | 1,36323 | 3,63192 | 5,46212 | 4,65014 | map00941 |
| --- | --- | --- | --- | --- | --- | --- | --- |
| Dioal.15G024400.1 | 1,55976 | -0,09949 | 1,94297 | 2,6602 | 2,1817 | 2,487 | map00941 |
| Dioal.15G024500.1 | 4,44032 | 4,00679 | 4,79973 | 4,64835 | 5,0409 | 4,43289 | map00941 |
| Dioal.15G024600.1 | 2,73903 | 3,9222 | 2,02852 | 4,4672 | 2,94983 | 4,13915 | map00941 |
| Dioal.15G024700.1 | 6,9661 | 7,71086 | 6,85729 | 7,36459 | 8,04147 | 7,71694 | map00941 |
| Dioal.15G024800.1 | 2,49684 | 4,19817 | 3,52864 | 3,75891 | 4,47589 | 3,04095 | map00941 |
| Dioal.15G024900.1 | -0,22717 | -0,372042 | -2,27576 | -1,57898 | 0,63026 | -0,928663 | map00941 |
| Dioal.15G025000.1 | 2,62053 | 1,76523 | 1,69829 | 2,03859 | 1,43761 | 1,5368 | map00941 |
| Dioal.15G025100.1 | 0,368677 | 3,71179 | 3,05339 | 4,70784 | 4,60368 | 5,09776 | map00941 |
| Dioal.17G061600.1 | 0,179977 | 1,69901 | 1,10067 | 1,2126 | 2,6453 | -0,202932 | map00941 |
| Dioal.17G061800.1 | -0,618745 | 0,363717 | -2,83303 | 1,68783 | 1,43691 | 1,98963 | map00941 |
| Dioal.17G061900.1 | 2,89615 | 3,41865 | 0,917865 | 5,07913 | 6,36872 | 4,27714 | map00941 |
| Dioal.17G062100.1 | 2,66113 | 3,35378 | 0,356855 | 4,19104 | 4,13774 | 2,64651 | map00941 |
| Dioal.17G071000.1 | 0 | 0 | 0 | 0 | 0 | 0 | map00941 |
| Dioal.17G071100.1 | 0 | 0 | 0 | 0 | 0 | 0 | map00941 |
| Dioal.17G082800.1 | -3,01133 | 0 | -1,09972 | -2,59551 | -0,864574 | -0,780897 | map00941 |
| Dioal.17G104600.1 | -3,42283 | -1,6322 | 0 | -1,12158 | -2,80364 | -0,947287 | map00941 |
| Dioal.17G104700.1 | 0 0 0 | | | 0 | -1,78636 | 0 | map00941 |
| Dioal.17G104800.1 | -0,92805 | 0 | 0 | -2,00284 | -1,68576 | 0 | map00941 |
| Dioal.17G104900.1 | -1,70203 | -2,16366 | 0 | -2,69297 | -2,27532 | -2,94866 | map00941 |
| Dioal.17G105000.1 | -1,54124 | 0 | 0,125519 | -2,34607 | -0,494987 | -1,40071 | map00941 |
| Dioal.17G105100.1 | -3,39281 | -1,6322 | -2,31337 | -1,79814 | 1,30462 | -1,9275 | map00941 |
| Dioal.17G105200.1 | -1,99706 -4,19933 | | -2,2214 | -0,871439 | 3,83455 | -2,63342 | map00941 |

| Dioal.17G105300.1 | 0 | -1,69368 | -1,08875 | -1,47131 | -1,76048 | -0,944017 | map00941 |
| --- | --- | --- | --- | --- | --- | --- | --- |
| Dioal.17G105400.1 | 0 | 0 | 0 | 0 | 0 | 0 | map00941 |
| Dioal.17G105500.1 | 0 | 0 | 0 | 0 | 0 | 0 | map00941 |
| Dioal.17G105600.1 | 0 | -1,44703 | 0 | 0 | -1,68777 | -1,76339 | map00941 |
| Dioal.17G105700.1 | 0 | 0 | 0 | 0 | 0 | 0 | map00941 |
| Dioal.17G106300.1 | -2,56297 | 0 | -1,58462 | -1,64138 | 1,97077 | -2,30666 | map00941 |
| Dioal.17G106500.1 | -0,879903 | 0 | 0 | -1,73569 | 1,58816 | -0,629977 | map00941 |
| Dioal.17G106700.1 | -1,38194 | -2,88306 | -1,141 | -1,67329 | 1,70014 | -4,28773 | map00941 |
| Dioal.17G106800.1 | -2,5228 | -1,82929 | -1,33745 | -0,174837 | 1,12243 | 1,31578 | map00941 |

Dioal.17G106900.1 Dioal.17G107000.1 Dioal.17G107100.1 Dioal.17G107200.1 Dioal.17G109200.1 Dioal.17G109300.1 Dioal.17G109500.1 Dioal.18G009000.1 Dioal.18G031000.1 Dioal.18G044100.1 Dioal.18G048800.1 Dioal.18G096100.1 Dioal.18G096200.1 Dioal.18G096800.1 Dioal.19G048600.1 Dioal.19G053900.1 Dioal.19G083800.1 Dioal.19G084100.1 Dioal.19G084200.1 Dioal.19G084300.1 Dioal.19G120300.1 Dioal.20G036100.1 Dioal.20G036200.1 Dioal.20G036300.1

| 0 | -1,6587 | 0 0 | | -1,72448 | 0  0 |
| --- | --- | --- | --- | --- | --- |
| 0 | 0 | 0 | -1,3742 | -1,32685 |  |
| -0,471241 | -1,8479 | -1,9941 | 0,031188 | 5,82053 | -1,51713 |
| 0 | 0 | 0 | 0 | 0 | 0 |
| 0,035416 | -2,82395 | -2,69888 | 0,537853 | 1,73016 | -0,305954 |
| -3,33963 | 0 | -1,24312 | -2,56968 | -1,53221 | -2,76093 |
| 3,20335 | 3,50155 | 3,80424 | 2,72182 | 3,8448 | 3,19967 |
| 0,964661 | 1,14076 | 1,23093 | 1,09085 | 1,89186 | 1,14452 |
| 0 | 0 | 0 | 0 | 0 | 0 |
| -3,44433 | 0 | -1,27785 | 0 | 0 | 0 |
| -0,311003 | 0,82079 | -0,999094 | 1,66786 | -1,8186 | 0,649144 |
| 0 | -1,97409 | -1,21627 | -2,22373 | -1,74385 | -0,787703 |
| 0 | 0 | 0 | 0 | 0 | 0 |
| 0 | 0 | 0 | 0 | -3,01655 | 0 |
| 0,00484 | 0,338943 | 0,279593 | 0,598892 | 3,72934 | 4,0253 |
| 3,42424 | 2,12842 | 2,65424 | 3,68581 | 5,84487 | 5,47106 |
| -2,83477 | 0 | -1,56554 | -2,27239 | 1,31063 | 0 |
| 0 | 0 | -1,44797 | 0,390233 | 0 | -0,817207 |
| 0 | -1,12734 | 0 | -0,92307 | 0 | -2,1338 |
| -0,484542 | 2,16091 | -1,86202 | -1,71025 | -0,978502 |  |
|  |  |  |  |  | -1,91072 |
| 5,46267 | 4,25547 | 4,98872 | 5,47516 | 6,96312 | 6,63738 |
| -3,45941 | -3,89637 | -3,05453 | -1,3656 | -1,60684 | -0,70675 |
| 1,64435 | 0,8113 | 1,91964 | 3,2281 | 5,25817 | 2,52659 |
| 5,47674 | 3,76624 | 5,53789 | 5,92296 | 7,77658 | 7,15927 |

map00941 map00941 map00941 map00941 map00941 map00941 map00941 map00941 map00941 map00941 map00941 map00941 map00941 map00941 map00941 map00941 map00941 map00941 map00941 map00941 map00941 map00941 map00941 map00941
